# Supplementary material for: Home-based Intervention with Semaglutide Treatment of Neuroleptic-Related Prediabetes (HISTORI): protocol describing a prospective, randomised, placebo controlled and double-blinded multicentre trial
Source: BMJ Open. 2024 Mar 18;14(3):e077173. doi: 10.1136/bmjopen-2023-077173 (PMC10953037; doi:10.1136/bmjopen-2023-077173)
Supplement: Supplementary data [file bmjopen-2023-077173supp001.pdf]

Supplementary Table 1

HISTORI	Investigator inclusion	Patient acceptance to participate	Week	Week	Week	Week	Week	Week	Week	Week	Week
			1	5	9	15	19	23	28	30	36
	Physical appearance of patients at hospitals	X				X				X	
			Weekly injection of Semaglutide / placebo								
Consent approval	X										
Baseline number		X									
Evaluation of medical history	X	X									
In- and Exclusion criteria	X	X									
Randomization		X									
Physical examination		X									
Blood pressure	X		X			X				X	
Weight/BMI	X		X			X				X	
Full biochemical screening		X								X	
Safety blood test	X					X					
PET CT scan		Scheduled							Scheduled		
PANNS-6, SF-36v2, IWQOL-Lite		X				X				X	
MARS, SIMPAQ, and EQ-5D		X	X	X	X	X	X	X	X	X	
Cardiac autonomic neuropathy (VAGUS®)		X				X				X	

**Adjustment of Semaglutide/placebo doses**				X	X						
**Semaglutide 0.25 mg**			X								
**Semaglutide 0.50 mg**				X							
**Semaglutide 1.00 mg per week**					X	X	X	X	X	X	
**End of study evaluation**											X
