# Supplementary material for: Home-based Intervention with Semaglutide Treatment of Neuroleptic-Related Prediabetes (HISTORI): protocol describing a prospective, randomised, placebo controlled and double-blinded multicentre trial
Source: BMJ Open. 2024 Mar 18;14(3):e077173. doi: 10.1136/bmjopen-2023-077173 (PMC10953037; doi:10.1136/bmjopen-2023-077173)
Supplement: Author's manuscript [file bmjopen-2023-077173.draft_revisions.pdf]

BMJ Open

BMJ Open is committed to open peer review. As part of this commitment we make the peer review history of every article we publish publicly available.

When an article is published we post the peer reviewers' comments and the authors' responses online. We also post the versions of the paper that were used during peer review. These are the versions that the peer review comments apply to.

The versions of the paper that follow are the versions that were submitted during the peer review process. They are not the versions of record or the final published versions. They should not be cited or distributed as the published version of this manuscript.

BMJ Open is an open access journal and the full, final, typeset and author-corrected version of record of the manuscript is available on our site with no access controls, subscription charges or pay-per-view fees (http://bmjopen.bmj.com).

If you have any questions on BMJ Open's open peer review process please email info.bmjopen@bmj.com

BMJ Open

Home based Intervention with Semaglutide Treatment of Neuroleptic-Related Prediabetes (HISTORI): protocol describing a prospective, randomized, placebo controlled and double blinded multicenter trial

Journal:	*BMJ Open*
Manuscript ID	bmjopen-2023-077173
Article Type:	Protocol
Date Submitted by the Author:	19-Jul-2023

Complete List of Authors:	Ganeshalingam, Ashok; Odense Universitetshospital, Endocrine Research Unit, Department of Endocrinology, Odense University Hospital & Department of Clinical Research, Faculty of Health, ; Lillebaelt Hospital - University Hospital of Southern Denmark, Department of Internal Medicine<br>Uhrenholt, Nicolai; Psykiatrien Region Sjælland Psykiatrien Vest<br>Arnfred, Sidse; University of Copenhagen,<br>Gæde, Peter; Slagelse Hospital, Department of Cardiology and Endocrinology<br>Bilenberg, Niels; University of Southern Denmark, Department of Clinical Research, Faculty of Health Sciences, University of Southern Denmark, Odense, Denmark.; University of Southern Denmark, Department of Child and Adolescent Mental Health Odense, Mental Health Services in the Region of Southern Denmark, University of Southern Denmark, Odense, Denmark.<br>Frystyk, Jan; Odense University Hospital Department of Endocrinology

Keywords:	PREVENTIVE MEDICINE, Primary Prevention, Diabetes & endocrinology < INTERNAL MEDICINE, Schizophrenia & psychotic disorders < PSYCHIATRY, General diabetes < DIABETES & ENDOCRINOLOGY

SCHOLARONE™
Manuscripts

HISTORI

Home based Intervention with Semaglutide Treatment of Neuroleptic-Related Prediabetes (HISTORI): protocol describing a prospective, randomized, placebo controlled and double blinded multicenter trial

For peer review only

A. Ganeshalingam [1,2,3,6], N. Uhrenholt [4], S. Arnfred [4], P. Gæde [5], N. Bilenberg [3], J. Frystyk [1]

1. Endocrine Research Unit, Department of Endocrinology, Odense University Hospital & Department of Clinical Research, Faculty of Health, University of Southern Denmark, Odense, Denmark
2. Department of Internal Medicine, Lillebaelt Hospital, University Hospital of Southern Denmark, Kolding, Denmark
3. Mental Health Services in the Region of Southern Denmark, Child and Adolescent Psychiatry Odense, Odense C, Denmark
4. Psychiatry West, Region Zealand, Research Unit for Psychotherapy and Psychopathology, Slagelse, Denmark
5. Næstved, Slagelse og Ringsted Hospital, Department of cardiology and endocrinology, Slagelse, Denmark
6. Steno Diabetes Center Odense, Odense University Hospital, Denmark

Running title: Patients with prediabetes and schizophrenia on Semaglutide

Keywords

Words in main text: 4279
Number of Tables: 2
Number of Figures: 1
Number of References: 52

Corresponding author

Dr. Ashok Ganeshalingam
Department of Endocrinology
Odense University Hospital
Kløvervænget 6, entrance 93
DK-5000 Odense C, Denmark.
E-mail: Ashok.Ganeshalingam@rsyd.dk

For peer review only - http://bmjopen.bmj.com/site/about/guidelines.xhtml

ABSTRACT

Introduction: Subjects with schizophrenia have a 2 to 3 fold higher mortality rate than the general population, and a reduced life expectancy of 10 to 20 years. Approximately 1/3 of this excess mortality has been attributed to obesity-related type 2 diabetes (T2D) and to cardiovascular disease (CVD). Glucagon-like peptide-1 (GLP-1) analogs increase satiety and delay gastric emptying, thereby reducing food intake and weight. GLP-1 analogs also exert beneficial effects on cardiovascular outcomes in high-risk T2D patients.

Our aim is to investigate whether 30 weeks add-on treatment with the GLP-1 analog Semaglutide can reduce HbA1c sufficiently to reverse prediabetes and the metabolic syndrome (MetS) in overweight schizophrenic patients.

Methods and analysis: We will perform a 30 week, two-armed, multi-center, superiority, double-blinded, randomized trial investigating the effect of weekly injections of Semaglutide vs placebo in mental health facilities in Region of Southern Denmark and Region of Zealand, Denmark. In total 154 adults with schizophrenia spectrum disease, aged 18 - 60 years treated with second generation anti-psychotic (SGA) treatment, HbA1c 39-47 mmol/mol and BMI > 27 kg/m^2 will be randomized to injections of 1.0 mg Semaglutide or placebo. The primary outcome is changes in HbA1c. Secondary outcomes encompass metabolic measures, insulin sensitivity, cardiovascular risk profile, and questionnaires and interviews regarding psychotic symptoms, quality of life, medication adherence, general well-being and physical activity.

This study will be carried out in accordance with the Helsinki Declaration, Good Clinical Practice guidelines, the Danish Medicine Agency and the regional scientific ethic committee of Region of Southern Denmark.

For peer review only - http://bmjopen.bmj.com/site/about/guidelines.xhtml

Trial Registration number NCT05193578 European Clinical Trials Database Number (EudraCT)

2020-004374-22, Regional Ethical Committee number S-20200182

Based on protocol version 3.2, approved 19th April 2023.

For peer review only

For peer review only - http://bmjopen.bmj.com/site/about/guidelines.xhtml

BACKGROUND AND SIGNIFICANCE OF HISTORI

The life expectancy of subjects with schizophrenia spectrum disorders (hereafter schizophrenia) is reduced by 10 to 20 years when compared to the general population (Nordentoft et al., 2013; Lawrence et al., 2013; Laursen et al., 2019; Correll et al., 2022). The excess mortality is partly explained by a 2 to 3 fold increase in cardiovascular disease (CVD) (Nordentoft et al., 2013; Westman et al., 2018). A nationwide register study from Sweden, including more than 46,000 subjects with schizophrenia showed that CVD was the leading cause of death, causing more deaths than suicide, and that death from CVD occurred 10 years earlier in schizophrenic subjects than in the general population. Furthermore, the mortality rate ratio for CVD was 6.16 in schizophrenic patients aged 15-59 years, as compared to 2.80 in all age groups. Thus, in schizophrenic subjects the development of CVD is accelerated and affects younger adults (Westman et al., 2018).

Another factor predisposing to the reduced life expectancy is type 2 diabetes (T2D), which develops in 10 to 15 % of all patients with schizophrenia (M. De Hert et al., 2009). Compared to the general population, patients with schizophrenia are more likely to have diabetes, hypertension, dyslipidemia, to be overweight and to smoke (Marc De Hert et al., 2011). Indeed, a third of patients with schizophrenia have been reported to suffer from the metabolic syndrome (MetS) (Vancampfort et al., 2015).

Antipsychotic treatment is vital to reduce schizophrenia related symptoms (Leucht et al., 2013) and treatment with second generation anti-psychotic (SGA) drugs has demonstrated beneficial effects on mortality, suicide rates and hospitalizations (Tiihonen et al., 2006). However, SGA treatment comes with unfortunate side effects, which typically include increased risk of weight gain and obesity (Maayan & Correll, 2011), and accordingly, the development of MetS and prediabetes (Pillinger et al., 2020). Furthermore, SGA treatment *per se* leads to impaired glucose tolerance, diabetes, hyperlipidemia and CVD (Henderson et al., 2015; Marc De Hert et al., 2011).

For peer review only - http://bmjopen.bmj.com/site/about/guidelines.xhtml

Gaining weight, and becoming overweight or even obese is associated with a reduced quality of life (QoL) in people with schizophrenia (Allison et al., 2003; Faulkner et al., 2007; Fond et al., 2020; R.L. et al., 2008; Strassnig et al., 2003; Sugawara et al., 2013). In addition, weight gain and MetS are among the most frequently reported reasons for discontinuation of treatment in this group (Lieberman et al., 2005). Indeed, in a cohort of 304 subjects with schizophrenia, 94% reported distress over body weight, and those who were obese appeared to be more than twice as likely to be non-adherent compared to those with a BMI below 25 kg/m^2 (Weiden et al., 2004). Therefore, it may be speculated that weight-reducing treatments may lead to an increased adherence to SGA treatment. However, to the best of our knowledge the association between a Glucagon-like Peptide 1 (GLP-1) agonist-induced weight reduction, the severity of psychotic symptoms, and the ability to adhere to SGA treatment has never been investigated in a randomized clinical trial (RCT).

Lifestyle and environmental factors such as smoking, poor diet and an insufficient capacity to exercise contribute to the risk of CVD (Henderson et al., 2015). Behavioral lifestyle interventions aiming at improving diet and physical activity have indeed shown short-term beneficial effects on body weight. However, the long term course indicates that benefits diminish over time compared to standard care (Cooper et al., 2016; Holt et al., 2019). When lifestyle programs fail to reduce the increased risk of CVD and T2D, metformin is standard treatment according to NICE guidelines (NICE 2012). In a meta-analysis of 19 RCTs (N=1,279) the addition of metformin to ongoing anti-psychotic treatment for an average of 3 to 4 months in subjects with severe mental illness significantly reduced body weight but only by 0.61 kg (Zheng et al., 2015). Therefore, alternative weight reducing drugs are required, e.g. GLP-1 analogs.

The potential of GLP-1 analogs to improve the metabolic profile of patients with schizophrenia was recently successfully tested. In a randomized controlled trial, including 97 patients (placebo/active comparator: 50/47) with schizophrenia, prediabetes and a BMI ≥27 kg/m^2, 16 weeks of Liraglutide (1.8 mg Victoza® daily) reduced body weight, waist circumference and abdominal fat mass, LDL cholesterol, glucose levels and insulin resistance, without any worsening in the psychiatric disorder (Larsen et al., 2017). The beneficial effect

For peer review only - http://bmjopen.bmj.com/site/about/guidelines.xhtml

of GLP-1 analog treatment in SGA-treated patients was later re-confirmed in a 2018 meta-analysis which, however, only included two additional and smaller trials of Exanatide (Bydureon®), and only one of these studies was placebo-controlled (D Siskind et al., 2017). Nevertheless, the review concluded that GLP-1 analog treatment is effective and tolerable for SGA-related weight gain, but that more studies are required (Kouidrat & Amad, 2019; Dan Siskind et al., 2019).

Liraglutide and Semaglutide do not have prediabetes as an indication but it may be suitable for treatment of prediabetes in patients with obesity. However, Liraglutide requires daily injections. This might be an obstacle as patients with schizophrenia are known for reduced medication adherence (Fernández, 2016). Semaglutide, on the other hand, requires weekly injections and this opens for the possibility of administering medication by mobile study nurses performing house calls. This may improve adherence to treatment and study protocol, increase the data quality of the study (e.g. collection of blood samples, anthropometric and safety data, as well as questionnaires) and at the same time be less stressful to the enrolled patients as they remain at home during data collection.

Aim and hypothesis

The aim of this study is to perform a randomized placebo-controlled double-blinded trial of Semaglutide 1.0 mg in comparison to placebo in obese individuals with prediabetes and schizophrenia spectrum disorders (SZD), i.e., either schizophrenia, schizotypal disorder or schizoaffective disorder.

We hypothesize that administration of Semaglutide at a dose of 1.0 mg administered subcutaneously once a week for 30 weeks improves HbA1c (primary outcome) in SGA-treated young adults with SZD suffering from prediabetes (HbA1c between 37 and 47 mmol/mol) and a BMI ≥27 kg/m^2, when compared to placebo. Additionally, that Semaglutide improves the following secondary endpoints: weight, schizophrenic symptoms (PANSS-6), QoL-related measures, patient-related outcomes (PRO data) and the cardiometabolic risk factor profile.

For peer review only - http://bmjopen.bmj.com/site/about/guidelines.xhtml

METHOD

Design. The current trial is an investigator-initiated, double-blinded, parallel, superiority, multi-center RCT with a 1:1 randomization. In total, we will include 154 patients recruited from two Danish regional mental health services (MHS). A CONSORT diagram is provided in **Figure 1**. Data management is purely digital and managed by Open Patient data Explorative Network (OPEN), Odense University Hospital, Denmark, using REDCAP ©.

SETTING

The study will recruit patients from community mental health centers and private practitioners in the Region of Southern Denmark and the Region of Zealand, Denmark.

PARTICIPANTS

Patients with a clinical diagnosis of either schizophrenia, schizotypal disorder or schizoaffective disorder (ICD-10 diagnoses: F20.x; F21; F25.x), age between 18 and 60 years receiving treatment in the regional mental health care of participating centers, or by private practice, and fulfilling criteria as listed in **Table 1**.

PROCEDURES

Recruitment

Patients fulfilling inclusion criteria will be invited from community mental health center clinics and private practices in the Region of Southern Denmark and the Region of Zealand. According to Danish clinical guidelines, patients treated with neuroleptics (almost exclusively SGAs) require minimum one annual laboratory test and clinical evaluation to test for the presence of the MetS. Eligible subjects accepting to participate will be invited to screening visits and allocated to treatment with placebo or Semaglutide at a dose of 1.0 mg administered subcutaneously once a week by blinded randomization, if all inclusion criteria and none of the exclusion criteria are met. The PhD students will be in charge of recruitment participants,

For peer review only - http://bmjopen.bmj.com/site/about/guidelines.xhtml

For peer review only

obtaining consent to participation, initiation of medication and execution of the Trial including inclusion visits and follow-up visits.

Randomization and concealment

A total of 154 participants will be enrolled and randomized 1:1 to Semaglutide or placebo. Block randomization including blocks of either 4 or 6 randomization numbers will be used, and the randomizations list will be provided by the manufacturer of Semaglutide and placebo (Novo Nordisk A/S, Novo Allé, Bagsværd DK-2880 Denmark). Randomization will be performed using REDCAP ©.

Mobile project nurses

The presence of schizophrenia impacts adherence and accordingly, adherence to study medication may be a challenge (El Abdellati et al., 2020). Therefore, we perform data collection at home by mobile study nurses to ensure optimal adherence and to minimize discomfort for the participants.

The study nurse will visit the subjects in their homes to administer injection and teach eligible subjects to self-inject under supervision. **Thus, apart from the enrolment, we plan for a home-based study.** If some patients are able to adhere to treatment and to self-inject, weekly visits may not be necessary. Thus, the frequency of home visits may be downscaled to involve monthly collection of data only. All data collection is allocated to study nurses and the PhD-students, and the participants will maintain management-as-usual (MAU) in the respective clinics from where they are recruited.

Interventions

The intervention consists of 30 weeks of treatment with once-weekly subcutaneous injections of either Semaglutide (1.34 mg/ml; 1.0 mg) or placebo. The current dose adjustment guidelines for Semaglutide will

For peer review only - http://bmjopen.bmj.com/site/about/guidelines.xhtml

be followed (i.e., starting at 0.25 mg weekly for 4 weeks, then 0.5 mg weekly for 4 weeks, and finally 1 mg or highest tolerated weekly dose until the end of intervention) or placebo (corresponding volume). Subjects, who experience side effects that hinder a stepwise increase in study drug, will remain 1.0 mg or at the highest possible tolerated dose of Semaglutide or placebo for the rest of the study.

Relevant concomitant care and interventions are permitted during the trial.

OUTCOMES

Primary outcome

Absolute reduction in HbA1c from baseline to end of treatment (30 weeks).

Secondary outcomes

During the treatment and after 30 weeks of treatment:

a) **Metabolic measures**: BMI, blood pressure, triglyceride, cholesterol (HDL and LDL), waist circumference and HOMA-estimates based on fasting insulin and plasma glucose.
   Blood test will be taken at the nearest hospital prior to inclusion, at the inclusion, at 15 weeks and at end of study. BMI, waist circumference and blood pressure will be measured at week 1, 15 and 30.
b) **Cardiovascular risk markers:** cardiovascular autonomic neuropathy (CAN) (Vagus TM®), and circulating levels of cardiovascular risk markers. CAN is considered a serious complication of diabetes, being associated with a 5-fold increased risk of CVD (Serhiyenko & Serhiyenko, 2018). Of interest to this study, CAN may be present prior to the development of diabetes (Herder et al., 2019) and it can be improved by weight loss (Poirier et al., 2003). As CAN may be measured by a hand held apparatus (Vagus TM®), measurements are easily performed in the homes of the enrolled patients. CAN will be measured at inclusion, at 15 weeks and at end of study.

For peer review only - http://bmjopen.bmj.com/site/about/guidelines.xhtml

c) **Pro-atherosclerotic changes:** we want to perform a pilot study in a subsample of 20 participants from placebo vs. 20 participants from the Semaglutide group, focusing on pre-symptomatic atherosclerosis assessed by positron emission tomography/computed tomography (PET/CT) using 18F-sodium fluoride (NaF) as tracer. NaF-PET/CT is a novel way of detecting and measuring calcification in major arteries, potentially years or decades before manifest arterial wall calcification becomes detectable by cardiac CT (McKenney-Drake et al., 2018; Moghbel et al., 2018). We plan to perform two NaFPET/CT acquisitions, i.e. at baseline and after 30 weeks treatment. Based on our experience, inclusion of 20 placebo- and 20 Semaglutide-treated subjects is suitable to perform the pilot study. We hypothesize a ≥10% decrease in global disease score (Høilund-Carlsen et al., 2019) as measured by NaF uptake in the heart and/or thoracic aorta, will be present after 30 weeks treatment.

d) **Positive and Negative Symptom Scale 6 (PANSS-6):** PANSS is an operationalized rating of positive and negative symptoms, in people with schizophrenia, which is sensitive to change (Kay et al., 1987). PANSS-6 is a shorter version of the original 30-item version. It is validated and translated into Danish and has proven a good level of inter-rater reliability when rated based on the Simplified Negative and Positive Symptoms Interview (SNAPSI) (Kølbæk et al., 2018; Østergaard et al., 2018) .PANSS-6 interview will be performed at baseline, and after 15 and 30 weeks.

e) **Impact of Weight on Quality of Life-Lite (IWQOL-Lite):** IWQOL-Lite is a reliable and valid self-report measure for assessing weight-related QoL in people with schizophrenia (Kolotkin et al., 2006). It provides a total score plus scores on five domains (physical function, self-esteem, sexual life, public distress, and work). It is translated into 83 languages, including Danish. IWQOL-Lite will be measured at baseline, and after 15 and 30 weeks.

f) **Short Form Survey (SF-36)** is a well-researched, self-reported measure of health. It comprises 36 questions in 8 different domains. The survey will be applied in Danish, using the latest updated

For peer review only - http://bmjopen.bmj.com/site/about/guidelines.xhtml

version 2 (SF-36v2) (Ware & Sherbourne, 1992). SF-36v2 will be measured at baseline, and after 15 and 30 weeks.

g) **Medication Adherence Rating Scale (MARS):** MARS is a validated and reliable measure of adherence for psychoactive medication. MARS constitutes 10 items rating the medical adherence (Thompson et al., 2000; Fialko et al., 2008)). MARS will be measured at baseline, and at weeks 5, 9, 15, 19, 23, 28 and 30.

h) **The Simple Physical Activity Questionnaire (SIMPAQ):** SIMPAQ has shown good test-retest reliability and has been validated against measures from Actigraph accelerometers (Rosenbaum et al., 2020). SIMPAQ will be measured at baseline, and at weeks 5, 9, 15, 19, 23, 28 and 30.

i) **The EuroQol five-dimensional (EQ-5D)** questionnaire is a widely applied instrument for the measurement and cost-benefit evaluation of health interventions (Rabin et al., 2014). EQ-5D will be measured at baseline, and at weeks 5, 9, 15, 19, 23, 28 and 30.

SAFETY ASSESSMENTS

Adverse reactions will be evaluated weekly. At every 4-week visit, vital parameters will be recorded, including blood pressure, body weight and waist circumference. The safety parameters in the study include adverse reactions, vital parameters and liver and kidney function, which will be analyzed at baseline, week 15 and week 30. If any side effects or changes in biochemical measures are observed, the trial physician or the physician responsible for the test will be informed.

All safety parameters will be recorded in an electronic case report form (eCRF) as soon as they have either been measured or reported by the study participant. The study will be monitored by good clinical praxis (GCP) and follow current GCP guidelines. The study protocol has been reviewed by GCP prior to inclusion of first participant, and subsequently, the study is monitored by GCP throughout the study period.

For peer review only

For peer review only - http://bmjopen.bmj.com/site/about/guidelines.xhtml

After drug administration, all potential side effects and other adverse events (AEs) observed will be reported in the eCRF. Semaglutide half-life is one week and thus expected to be completely eliminated after six weeks). Consequently, collection of AEs for all patients will be completed six weeks after the final injection by telephone calls. The nature of each AE will be assessed according to the severity and relationship of the experimental medicine or trial procedure, and will be assessed by the primary investigator, who is a physician. If it is considered that the AE is related to the test drug or test procedure, it will be noted as a side effect in the eCRF. All severe adverse events (SAEs) will be reported to the sponsor as soon as possible, but always within 24 hours of it coming to attention. All severe adverse reactions (SARs) which, regardless of dose, result in death, are life-threatening, result in hospitalization or prolongation of hospital stay in medical department, result in significant or persistent disability or incapacity, or lead to congenital anomaly or malformation, will be considered in this trial to be unexpected (SUSAR).

Emergency un-blinding will occur if the participants develop adverse events that require knowledge of the treatment, if patient develop sign of overdose or if patient develop conditions that require treatment with the trial drug (Semaglutide) or drugs with potential interactions with trial drug.

STATISTICAL CONSIDERATIONS

Primary outcomes for the study will be absolute changes in HbA1c. Changes in HbA1c will be evaluated after 15 weeks and after 30 weeks.

Assuming that 30 weeks of Semaglutide reduces HbA1c by 0.2%, and that SD equals 0.35%, **65 subjects in each arm are required to obtain a power of 90%**, using a 2-sided significance level of 5%.

For peer review only - http://bmjopen.bmj.com/site/about/guidelines.xhtml

Dropout from trials concerning pharmacological treatment for obesity is non-linear; the dropout from the treatment group will occur earlier due to side effects, while in the placebo group dropouts occur later due to lack of efficacy.

Feasibility

In the SUSTAIN 1 trial (30 weeks of Semaglutide in patients with T2D), the discontinuation of study drug amounted to 13% in those receiving 0.5 mg Semaglutide, 12% in those receiving 1.0 mg Semaglutide, and 11% in placebo-treated subjects (Sorli et al., 2017). In the 16-week study with Liraglutide in patients with schizophrenia, 23 of 214 patients declined to participate, and 111 patients did not meet inclusion criteria, leaving 103 to be enrolled. Of these, only 7 patients dropped out of the study, leaving an overall success rate of 45 percent (Larsen et al., 2017b). We believe to experience similar rates. Thus, we need to approach approximately 350 patients to enroll 154 patients. Given the number of potentially eligible patients in the two regions (currently estimated to >300 excluding new cases in the age 18-60 years), we believe this is feasible. In the present study, we will follow the participants for 30 weeks. Hence, we predict a higher risk of dropout. Accordingly, we conservatively assume a dropout rate of 15 percent to ensure that the required number of subjects to obtain power are recruited.

Although this study is not designed as treatment for obesity, we believe the estimation of dropout is reliable as the study focus on the same patient population and the pharmacological profile of the investigated drug is similar to Liraglutide (Larsen et al., 2017b).

DATA ANALYSIS

All participants randomized in the study will be included in the assessment of endpoints. All data will be analyzed based on the intention-to-treat population. Clinical results will be presented as mean, least squares mean, least square mean change from baseline or least square difference between groups with SE,

For peer review only - http://bmjopen.bmj.com/site/about/guidelines.xhtml

SD or two-sided 95% CI as appropriate. Statistical tests will be conducted as two-sided tests with a 5% significance level.

A mixed effects linear regression analysis will be used to compare changes in glucose tolerance, HbA1c, from baseline to 30 weeks follow-up between the intervention and the placebo groups. Missing values will be imputed, primary analysis will be intention-to-treat. Supplementary per protocol analysis will accompany the intention to treat analysis.

All changes in secondary outcomes from baseline to various follow-up times are analyzed using mixed effects linear regression analyses for continuous outcomes and mixed effects logistic regression analyses for categorical outcomes. All analyses on continuous outcomes are adjusted for the baseline value of the outcome, and in case of significant imbalances in baseline patient characteristics between the two randomized groups, exploratory analyses including such variables are conducted. To correct for multiple testing of the secondary outcomes, Holm's sequentially rejective multiple test procedure will be applied. Statistical analyses are performed using Stata software Version 16 (Statacorp, Texas, USA). The hypothesis tests are 2-sided, and the 5% level of significance is considered.

ETHICAL APPROVAL AND CONSENT TO PARTICIPATE

The project is approved by the Regional Science Ethical Committee for the Region of Southern Denmark and the Danish Medicine Agency. Data processing in connection with the study is reported to the region's list of on-going research projects via the Executive Secretariat at Odense University Hospital (OUH). The study participants will not necessarily be able to benefit from participation in the study. Individual participants may be diagnosed with illness that may require treatment; in that case, they will be referred for relevant treatment. Participants will not have overt diabetes based on HbA1c. Therefore, following national guidelines, participants in this study are not candidates for standard treatment of diabetes and

For peer review only - http://bmjopen.bmj.com/site/about/guidelines.xhtml

under normal circumstances they would not receive antidiabetic treatment or treatment that would reduce their risk of diabetes. However, Metformin treatment is accepted.

Amendments to the study protocol will be made to the Regional Science Ethical Committee for the Region of Southern Denmark and the Danish Medicine Agency.

The study will be conducted in accordance with the Helsinki Declaration with amendments as well as in accordance with the General Data Protection Regulation (GDPR), and the Danish Data Protection. In the case that biological material will be shipped to collaborators abroad, the Danish Protection Agency will be applied and Chapter V of the Danish Protection Act will be obeyed. It is mandatory that the clinical staff involved in the clinical trial has completed an official course in Good Clinical Practice.

TRIAL STATUS

Recruitment opens January 2022 with first patient, first visit (FPFV), whereas last patient, last visit (LPLV) is scheduled for **September 2024.** Analysis of primary endpoints is planned to **March 2025**: Data will be published in EudraCT within 12 months from LPLV. Publication of the final results on primary endpoint is expected to be made public in **March 2025**. The results will be disseminated in peer review publications and conference presentations.

For peer review only

For peer review only - http://bmjopen.bmj.com/site/about/guidelines.xhtml

DISCUSSION

Metabolic disturbances, obesity and diabetes development in SGA treated patients are a major problem in the clinical setting, but the underlying mechanism is poorly understood, even though there are theories focusing on an interaction of medication, genes, life style, diet and physical activity (Henderson et al., 2015; Vancampfort et al., 2015; Reynolds & McGowan, 2017)

GLP-1 analogs have shown remarkable effect in the treatment of diabetes by contributing to weight loss, lowering blood glucose levels and improving mortality from CVD (Sorli et al., 2017). This is in contrast to life style interventions with physical activity and diet changes, as such initiatives have failed to induce robust long-term improvements of the metabolic disturbances (Speyer et al., 2016; Holt et al., 2019; Cooper et al., 2016)

The main focus of this clinical trial is mitigation of metabolic aberrations due to SGA treatment and reversal of prediabetes. Hence, the primary outcome is the absolute reduction in HbA1c rather than weight loss.

Patients with severe mental illness experience many challenges due to schizophrenic symptoms and side effects to SGA treatment (Maayan & Correll, 2011; Pillinger et al., 2020). Recruitment may be difficult in this patient population, and accordingly, we are collaborating with local psychiatric teams in both Regions and with local consultants to create a secure environment. The study is designed to focus on adherence to study protocol and minimize the patient's burden of study participation. The home visit by study nurses is likely to strengthen protocol adherence by assisting the participants and creating a secure and safe environment, eventually yielding higher reliability of data from interviews and questionnaires.

Semaglutide holds benefits compared to Liraglutide when dealing with patients with weight gain caused by SGAs. Semaglutide requires weekly treatment only, which makes it easier to administer with help from a study-nurse. In addition, the weight reducing effect of Semaglutide is greater than Liraglutide without

For peer review only - http://bmjopen.bmj.com/site/about/guidelines.xhtml

compromising the cardio-protective effect (Verma et al., 2022). Hence, if Semaglutide 1.34 mg/ml has a beneficial effect on weight, HbA1c, MetS and QoL, it could potentially be used in patients with schizophrenia in high risk of cardiovascular disease to prevent premature death due to diabetes.

Our study will attempt to mitigate the metabolic side effects of SGA induced overweight in individuals with schizophrenia and prediabetes. Treatment with Semaglutide improves glycemic control, weight and clinical cardiovascular risk factors by reducing numbers of fatal and non-fatal myocardial infarction and non-fatal stroke within 104 weeks (Marso et al., 2016). We hypothesize that beneficial pre-clinical events are detectable by NAF uptake using PET-CT scans after 30 weeks of treatment. If so, this may contribute to a better understanding of the nature behind the increased cardiovascular risk within these patients.

We hope that our trial HISTORI will provide insight in how to benefit the cardio-metabolic health and the QoL of schizophrenic patients – adding more and better years - and thereby change the HISTORY of this vulnerable group of patients. Additionally, if successful, HISTORI may pave the way for long-term, large-scale GLP-1 agonist interventions in schizophrenic patients with prediabetes, aiming to reduce the number of hardcore clinical cardiovascular endpoints, and thereby prolonging their life expectancy. Finally, a positive outcome may fuel initiatives to study the ability of GLP-1 agonists to prevent the development of the prediabetes, e.g. by starting treatment shortly after treatment with SGAs are initiated in high-risk patients.

FINANCIAL CONSIDERATIONS

Sources and types of financial, material, and other support funding for the study has been obtained from private foundations:

For peer review only - http://bmjopen.bmj.com/site/about/guidelines.xhtml

Novo Nordisk Foundation: 8,584,959DKK (salary and tuition fee PHD Students and study-nurses, running expenses, biochemical and laboratory procedures, TAP personal, mileage allowance)

Research Grant from Steno Diabetes Center Zealand, Denmark: 660,000 DKK.

Research Grant from Steno Diabetes Center Odense, Denmark: 578,000 DKK.

Annum (Region Zealand) to PhD-student Nicolai Uhrenholt: 20.000 DDK per year for three years (60,000 DKK in total).

Slagelse Puljen, 2020: The Region of Zealand, Denmark: 240,000 DKK

Slagelse Puljen 2022: 150,000 DKK

Region Sjællands Sundhedsvidenskabelige Forskningsfond 2022: 252,631 DKK. Furthermore, Novo Nordisk A/S will provide investigational drug and placebo free of charge for sponsor, but is otherwise not involved in the study. The scientists involved will conduct the experiment out of general scientific interest without personal financial gain. None of the investigators involved have financial interests (including shares, direct employment, members of advisory boards) in the drug company that produces the active drug used in the study. None of the participants in the study will receive any provision for participation. Participants will receive mileage allowances. Any harm as a result of study participation will be covered by the Danish Patient Compensation.

Roles and responsibilities

Authors' contributions:

For peer review only - http://bmjopen.bmj.com/site/about/guidelines.xhtml

AG, NU, SA, PG, NB, and JF initiated the study design, AG and NU helped with implementation and execution of the study. JF, NB, SA, and PGH are grant holders. JF and NB provided statistical expertise in clinical trial design, AG and NGU are conducting the primary statistical analysis.

AG made significant contributions to the study protocol in the design and the writing process in with contributions from NGU. All authors contributed to refinement of the study protocol and approved the final manuscript.

The Data from this study belongs to the research group behind Project HISTORI and can be accessed after application to the sponsor JF.

Sponsor:

Odense University Hospital

Steno Diabetes Centre Odense / Department of Endocrinology M. J.B. Winsløvvej 4. DK- 5000 Odense, Denmark.

Correspondence:

Jan Frystyk, Clinical Professor, DMsc, Ph.D. Steno Diabetes Centre Odense / Department of Endocrinology M. Odense University Hospital. J.B. Winsløvvej 4. DK- 5000 Odense, Denmark. Email: jan.frystyk@rsyd.dk

Primary Investigator at the Region of Southern Denmark:

Jan Frystyk, Clinical Professor, DMsc, Ph.D. Clinical management of diabetic patients at SDCO; implementation of biomarkers in clinical studies, and PI on clinical studies, hereunder GCP-monitored RCT. Department of Endocrinology M / Steno Diabetes Centre Odense (SDCO). Odense University Hospital. J.B. Winsløvvej 4. DK- 5000 Odense, Denmark.

Primary Investigator at the Region Zealand Community Psychiatry:

Sidse Arnfred, Clinical Professor.

Specialist in family medicine, psychiatry and board certified supervisor in psychotherapy (CBT). Research expertise in schizophrenia, psychopathology and psychophysiology. Experienced PI on RCT's, qualitative studies of user experiences and treatment evaluation. Psykiatrien Region Sjælland**,** Psykiatrien Vest, Fælledvej 6, bygning 3, 4. sal, DK- 4200 Slagelse, Denmark.

Primary Investigator at the Region of Southern Denmark community Psychiatry

For peer review only - http://bmjopen.bmj.com/site/about/guidelines.xhtml

For peer review only

Niels Bilenberg, Clinical Professor, Ph.D. OPUS clinics and community psychiatry centers of Region of Southern. Mental Health Hospital and University Clinic in the Region of Southern Denmark. J.B.Winsløws Vej 28 B, indg. 236, DK-5000 Odense, Denmark.

Co-investigators:

Peter Gæde, MD, DMSci, Chief Physician, Associate Professor. Department of Steno Diabetes Centre Sjælland. Steno Diabetes Center Sjælland, Akacievej 7, 1.sal. DK-4300 Holbæk, Denmark.

PhD students:

Ashok Ganeshalingam, Physician, Ph.D. Student. Department of Endocrinology M / Steno Diabetes Centre Odense (SDCO). Odense University Hospital. J.B. Winsløvvej 4. DK- 5000 Odense, Denmark.

Nicolai Gundtoft Uhrenholt, Physician, Ph.D. Student. Psykiatrien Region Sjælland**,** Psykiatrien Vest, Fælledvej 6, Bygning 3, 4. sal, DK- 4200 Slagelse, Denmark.

For peer review only - http://bmjopen.bmj.com/site/about/guidelines.xhtml

REFERENCES

Allison, D. B., Mackell, J. A., & McDonnell, D. D. (2003). The impact of weight gain on quality of life among persons with schizophrenia. *Psychiatric Services*, *54*(4), 565–567. https://doi.org/10.1176/appi.ps.54.4.565

Cooper, S. J., Reynolds, G. P., Barnes, T. R. E., England, E., Haddad, P. M., Heald, A., Holt, R. I. G., Lingford-Hughes, A., Osborn, D., McGowan, O., Patel, M. X., Paton, C., Reid, P., Shiers, D., & Smith, J. (2016). BAP guidelines on the management of weight gain, metabolic disturbances and cardiovascular risk associated with psychosis and antipsychotic drug treatment. *Journal of Psychopharmacology*. https://doi.org/10.1177/0269881116645254

Correll, C. U., Solmi, M., Croatto, G., Schneider, L. K., Rohani-Montez, S. C., Fairley, L., Smith, N., Bitter, I., Gorwood, P., Taipale, H., & Tiihonen, J. (2022). *World Psychiatry - 2022 - Correll - Mortality in people with schizophrenia a systematic review and meta-analysis of.pdf* (pp. 249–271).

De Hert, M., Dekker, J. M., Wood, D., Kahl, K. G., Holt, R. I. G., & Möller, H. J. (2009). Cardiovascular disease and diabetes in people with severe mental illness position statement from the European Psychiatric Association (EPA), supported by the European Association for the Study of Diabetes (EASD) and the European Society of Cardiology (ESC. In *European Psychiatry*. https://doi.org/10.1016/j.eurpsy.2009.01.005

De Hert, Marc, Correll, C. U., Bobes, J., Cetkovich-Bakmas, M., Cohen, D. A. N., Asai, I., Detraux, J., Gautam, S., Möller, H. J., Ndetei, D. M., Newcomer, J. W., Uwakwe, R., & Leucht, S. (2011). Physical illness in patients with severe mental disorders. I. Prevalence, impact of medications and disparities in health care. *World Psychiatry*, *10*(1), 52–77. https://doi.org/10.1002/j.2051-5545.2011.tb00014.x

El Abdellati, K., De Picker, L., & Morrens, M. (2020). Antipsychotic Treatment Failure: A Systematic Review on Risk Factors and Interventions for Treatment Adherence in Psychosis. *Frontiers in Neuroscience*,

For peer review only - http://bmjopen.bmj.com/site/about/guidelines.xhtml

14(October). https://doi.org/10.3389/fnins.2020.531763

Faulkner, G., Cohn, T., Remington, G., & Irving, H. (2007). Body mass index, waist circumference and quality of life in individuals with schizophrenia. *Schizophrenia Research*, *90*(1–3), 174–178. https://doi.org/10.1016/j.schres.2006.10.009

Fernández, S. G. (2016). Adherence to antipsychotic medication in bipolar disorder and schizophrenia patients: a systematic review. *European Neuropsychopharmacology*. https://doi.org/10.1016/s0924-977x(16)31412-2

Fialko, L., Garety, P. A., Kuipers, E., Dunn, G., Bebbington, P. E., Fowler, D., & Freeman, D. (2008). A large-scale validation study of the Medication Adherence Rating Scale (MARS). *Schizophrenia Research*, *100*(1–3), 53–59. https://doi.org/10.1016/j.schres.2007.10.029

Fond, G., Korchia, T., Sunhary de Verville, P. L., Godin, O., Schürhoff, F., Berna, F., André, M., Aouizerate, B., Capdevielle, D., Chereau, I., D'Amato, T., Dubertret, C., Dubreucq, J., Leignier, S., Mallet, J., Misdrahi, D., Passerieux, C., Pignon, B., Rey, R., … Boyer, L. (2020). Major depression, sleep, hostility and body mass index are associated with impaired quality of life in schizophrenia. Results from the FACE-SZ cohort. *Journal of Affective Disorders*, *274*(February 2020), 617–623. https://doi.org/10.1016/j.jad.2020.05.003

Henderson, D. C., Vincenzi, B., Andrea, N. V., Ulloa, M., & Copeland, P. M. (2015). Pathophysiological mechanisms of increased cardiometabolic risk in people with schizophrenia and other severe mental illnesses. *The Lancet Psychiatry*, *2*(5), 452–464. https://doi.org/10.1016/S2215-0366(15)00115-7

Herder, C., Roden, M., & Ziegler, D. (2019). Novel Insights into Sensorimotor and Cardiovascular Autonomic Neuropathy from Recent-Onset Diabetes and Population-Based Cohorts. In *Trends in Endocrinology and Metabolism*. https://doi.org/10.1016/j.tem.2019.02.007

Høilund-Carlsen, P. F., Moghbel, M. C., Gerke, O., & Alavi, A. (2019). Evolving Role of PET in Detecting and Characterizing Atherosclerosis. In *PET Clinics*. https://doi.org/10.1016/j.cpet.2018.12.001

For peer review only - http://bmjopen.bmj.com/site/about/guidelines.xhtml

Holt, R. I. G., Gossage-Worrall, R., Hind, D., Bradburn, M. J., McCrone, P., Morris, T., Edwardson, C., Barnard, K., Carey, M. E., Davies, M. J., Dickens, C. M., Doherty, Y., Etherington, A., French, P., Gaughran, F., Greenwood, K. E., Kalidindi, S., Khunti, K., Laugharne, R., … Wright, S. (2019). Structured lifestyle education for people with schizophrenia, schizoaffective disorder and first-episode psychosis (STEPWISE): Randomised controlled trial. *British Journal of Psychiatry*, *214*(2), 63–73. https://doi.org/10.1192/bjp.2018.167

Kay, S. R., Fiszbein, A., & Opler, L. A. (1987). The positive and negative syndrome scale (PANSS) for schizophrenia. *Schizophrenia Bulletin*. https://doi.org/10.1093/schbul/13.2.261

Kølbæk, P., Blicher, A. B., Buus, C. W., Feller, S. G., Holm, T., Dines, D., O'Leary, K. M., Sørensen, R. S., Opler, M., Correll, C. U., Mors, O., Bech, P., & Østergaard, S. D. (2018). Inter-rater reliability of ratings on the six-item Positive and Negative Syndrome Scale (PANSS-6) obtained using the Simplified Negative and Positive Symptoms Interview (SNAPSI). *Nordic Journal of Psychiatry*, *72*(6), 431–436. https://doi.org/10.1080/08039488.2018.1492014

Kolotkin, R. L., Crosby, R. D., Corey-Lisle, P. K., Li, H., & Swanson, J. M. (2006). Performance of a weight-related measure of Quality of Life in a psychiatric sample. *Quality of Life Research : An International Journal of Quality of Life Aspects of Treatment, Care and Rehabilitation*, *15*(4), 587–596. https://doi.org/10.1007/s11136-005-4627-4

Kouidrat, Y., & Amad, A. (2019). GLP-1 agonists for metabolic disorders in schizophrenia. In *Schizophrenia Research*. https://doi.org/10.1016/j.schres.2018.09.019

Larsen, J. R., Vedtofte, L., Jakobsen, M. S. L., Jespersen, H. R., Jakobsen, M. I., Svensson, C. K., Koyuncu, K., Schjerning, O., Oturai, P. S., Kjaer, A., Nielsen, J., Holst, J. J., Ekstrøm, C. T., Correll, C. U., Vilsbøll, T., & Fink-Jensen, A. (2017a). Effect of liraglutide treatment on prediabetes and overweight or obesity in clozapine- or olanzapine-treated patients with schizophrenia spectrum disorder: A randomized clinical trial. *JAMA Psychiatry*. https://doi.org/10.1001/jamapsychiatry.2017.1220

For peer review only - http://bmjopen.bmj.com/site/about/guidelines.xhtml

Larsen, J. R., Vedtofte, L., Jakobsen, M. S. L., Jespersen, H. R., Jakobsen, M. I., Svensson, C. K., Koyuncu, K., Schjerning, O., Oturai, P. S., Kjaer, A., Nielsen, J., Holst, J. J., Ekstrøm, C. T., Correll, C. U., Vilsbøll, T., & Fink-Jensen, A. (2017b). Effect of liraglutide treatment on prediabetes and overweight or obesity in clozapine- or olanzapine-treated patients with schizophrenia spectrum disorder: A randomized clinical trial. *JAMA Psychiatry*, *74*(7), 719–728. https://doi.org/10.1001/jamapsychiatry.2017.1220

Laursen, T. M., Plana-Ripoll, O., Andersen, P. K., McGrath, J. J., Toender, A., Nordentoft, M., Canudas-Romo, V., & Erlangsen, A. (2019). Cause-specific life years lost among persons diagnosed with schizophrenia: Is it getting better or worse? *Schizophrenia Research*, *206*, 284–290. https://doi.org/10.1016/j.schres.2018.11.003

Lawrence, D., Hancock, K. J., & Kisely, S. (2013). The gap in life expectancy from preventable physical illness in psychiatric patients in Western Australia: Retrospective analysis of population based registers. *BMJ (Online)*. https://doi.org/10.1136/bmj.f2539

Leucht, S., Cipriani, A., Spineli, L., Mavridis, D., Orey, D., Richter, F., Samara, M., Barbui, C., Engel, R. R., Geddes, J. R., Kissling, W., Stapf, M. P., Lässig, B., Salanti, G., & Davis, J. M. (2013). Comparative efficacy and tolerability of 15 antipsychotic drugs in schizophrenia: a multiple-treatments meta-analysis. *Lancet (London, England)*, *382*(9896), 951–962. https://doi.org/10.1016/S0140-6736(13)60733-3

Lieberman, J. A., Stroup, T. S., McEvoy, J. P., Swartz, M. S., Rosenheck, R. A., Perkins, D. O., Keefe, R. S. E., Davis, S. M., Davis, C. E., Lebowitz, B. D., Severe, J., & Hsiao, J. K. (2005). Effectiveness of antipsychotic drugs in patients with chronic schizophrenia. *The New England Journal of Medicine*, *353*(12), 1209–1223. https://doi.org/10.1056/NEJMoa051688

Maayan, L., & Correll, C. U. (2011). Weight gain and metabolic risks associated with antipsychotic medications in children and adolescents. *Journal of Child and Adolescent Psychopharmacology*, *21*(6), 517–535. https://doi.org/10.1089/cap.2011.0015

For peer review only

For peer review only - http://bmjopen.bmj.com/site/about/guidelines.xhtml

Marso, S. P., Bain, S. C., Consoli, A., Eliaschewitz, F. G., Jodar, E., Leiter, L. A., Lingvay, I., Rosenstock, J., Seufert, J., Warren, M. L., Woo, V., Hansen, O., Holst, A. G., Pettersson, J., & Vilsboll, T. (2016). Semaglutide and cardiovascular outcomes in patients with type 2 diabetes. *New England Journal of Medicine*. https://doi.org/10.1056/NEJMoa1607141

McKenney-Drake, M. L., Moghbel, M. C., Paydary, K., Alloosh, M., Houshmand, S., Moe, S., Salavati, A., Sturek, J. M., Territo, P. R., Weaver, C., Werner, T. J., Høilund-Carlsen, P. F., Sturek, M., & Alavi, A. (2018). 18F-NaF and 18F-FDG as molecular probes in the evaluation of atherosclerosis. In *European Journal of Nuclear Medicine and Molecular Imaging*. https://doi.org/10.1007/s00259-018-4078-0

Moghbel, M., Al-Zaghal, A., Werner, T. J., Constantinescu, C. M., Høilund-Carlsen, P. F., & Alavi, A. (2018). The Role of PET in Evaluating Atherosclerosis: A Critical Review. In *Seminars in Nuclear Medicine*. https://doi.org/10.1053/j.semnuclmed.2018.07.001

Nordentoft, M., Wahlbeck, K., Hällgren, J., Westman, J., Ösby, U., Alinaghizadeh, H., Gissler, M., & Laursen, T. M. (2013). Excess Mortality, Causes of Death and Life Expectancy in 270,770 Patients with Recent Onset of Mental Disorders in Denmark, Finland and Sweden. *PLoS ONE*, *8*(1). https://doi.org/10.1371/journal.pone.0055176

Østergaard, S. D., Foldager, L., Mors, O., Bech, P., & Correll, C. U. (2018). The Validity and Sensitivity of PANSS-6 in the Clinical Antipsychotic Trials of Intervention Effectiveness (CATIE) Study. *Schizophrenia Bulletin*, *44*(2), 453–462. https://doi.org/10.1093/schbul/sbx076

Pillinger, T., McCutcheon, R. A., Vano, L., Mizuno, Y., Arumuham, A., Hindley, G., Beck, K., Natesan, S., Efthimiou, O., Cipriani, A., & Howes, O. D. (2020). Comparative effects of 18 antipsychotics on metabolic function in patients with schizophrenia, predictors of metabolic dysregulation, and association with psychopathology: a systematic review and network meta-analysis. *The Lancet Psychiatry*, *7*(1), 64–77. https://doi.org/10.1016/S2215-0366(19)30416-X

Poirier, P., Hernandez, T. L., Weil, K. M., Shepard, T. J., & Eckel, R. H. (2003). Impact of diet-induced weight

For peer review only - http://bmjopen.bmj.com/site/about/guidelines.xhtml

loss on the cardiac autonomic nervous system in severe obesity. *Obesity Research*. https://doi.org/10.1038/oby.2003.143

R.L., K., P.K., C.-L., R.D., C., J.M., S., A.V., T., & G.J., L. (2008). Impact of obesity on health-related quality of life in schizophrenia and bipolar disorder. In *Obesity* (Vol. 16, Issue 4, pp. 749–754). http://ovidsp.ovid.com/ovidweb.cgi?T=JS&PAGE=reference&D=emed8&NEWS=N&AN=18239573

Rabin, R., Gudex, C., Selai, C., & Herdman, M. (2014). From translation to version management: a history and review of methods for the cultural adaptation of the EuroQol five-dimensional questionnaire. *Value in Health : The Journal of the International Society for Pharmacoeconomics and Outcomes Research*, *17*(1), 70–76. https://doi.org/10.1016/j.jval.2013.10.006

Reynolds, G. P., & McGowan, O. O. (2017). Mechanisms underlying metabolic disturbances associated with psychosis and antipsychotic drug treatment. *Journal of Psychopharmacology (Oxford, England)*, *31*(11), 1430–1436. https://doi.org/10.1177/0269881117722987

Rosenbaum, S., Morell, R., Abdel-Baki, A., Ahmadpanah, M., Anilkumar, T. V, Baie, L., Bauman, A., Bender, S., Boyan Han, J., Brand, S., Bratland-Sanda, S., Bueno-Antequera, J., Camaz Deslandes, A., Carneiro, L., Carraro, A., Castañeda, C. P., Castro Monteiro, F., Chapman, J., Chau, J. Y., … Ward, P. B. (2020). Assessing physical activity in people with mental illness: 23-country reliability and validity of the simple physical activity questionnaire (SIMPAQ). *BMC Psychiatry*, *20*(1), 108. https://doi.org/10.1186/s12888-020-2473-0

Serhiyenko, V. A., & Serhiyenko, A. A. (2018). Cardiac autonomic neuropathy: Risk factors, diagnosis and treatment. *World Journal of Diabetes*. https://doi.org/10.4239/wjd.v9.i1.1

Siskind, D, Russell, A., Gamble, C., Winckel, K., Hollingworth, S., & Kisely, S. (2017). RCT of exenatide for clozapine-associated obesity. *Schizophrenia Bulletin*.

Siskind, Dan, Hahn, M., Correll, C. U., Fink-Jensen, A., Russell, A. W., Bak, N., Broberg, B. V., Larsen, J., Ishøy, P. L., Vilsbøll, T., Knop, F. K., Kisely, S., & Ebdrup, B. H. (2019). Glucagon-like peptide-1 receptor

For peer review only - http://bmjopen.bmj.com/site/about/guidelines.xhtml

agonists for antipsychotic-associated cardio-metabolic risk factors: A systematic review and individual participant data meta-analysis. *Diabetes, Obesity and Metabolism*, *21*(2), 293–302. https://doi.org/10.1111/dom.13522

Sorli, C., Harashima, S. ichi, Tsoukas, G. M., Unger, J., Karsbøl, J. D., Hansen, T., & Bain, S. C. (2017). Efficacy and safety of once-weekly semaglutide monotherapy versus placebo in patients with type 2 diabetes (SUSTAIN 1): a double-blind, randomised, placebo-controlled, parallel-group, multinational, multicentre phase 3a trial. *The Lancet Diabetes and Endocrinology*. https://doi.org/10.1016/S2213-8587(17)30013-X

Speyer, H., Christian Brix Nørgaard, H., Birk, M., Karlsen, M., Storch Jakobsen, A., Pedersen, K., Hjorthøj, C., Pisinger, C., Gluud, C., Mors, O., Krogh, J., & Nordentoft, M. (2016). The CHANGE trial: No superiority of lifestyle coaching plus care coordination plus treatment as usual compared to treatment as usual alone in reducing risk of cardiovascular disease in adults with schizophrenia spectrum disorders and abdominal obesity. *World Psychiatry*, *15*(2), 155–165. https://doi.org/10.1002/wps.20318

Strassnig, M., Brar, J. S., & Ganguli, R. (2003). Body mass index and quality of life in community-dwelling patients with schizophrenia. *Schizophrenia Research*, *62*(1–2), 73–76. https://doi.org/10.1016/S0920-9964(02)00441-3

Sugawara, N., Yasui-Furukori, N., Sato, Y., Saito, M., Furukori, H., Nakagami, T., Kudo, S., & Kaneko, S. (2013). Body mass index and quality of life among outpatients with schizophrenia in Japan. *BMC Psychiatry*, *13*. https://doi.org/10.1186/1471-244X-13-108

Thompson, K., Kulkarni, J., & Sergejew, A. A. (2000). Reliability and validity of a new Medication Adherence Rating Scale (MARS) for the psychoses. *Schizophrenia Research*. https://doi.org/10.1016/S0920-9964(99)00130-9

Tiihonen, J., Wahlbeck, K., Lönnqvist, J., Klaukka, T., Ioannidis, J. P. A., Volavka, J., & Haukka, J. (2006). Effectiveness of antipsychotic treatments in a nationwide cohort of patients in community care after

For peer review only - http://bmjopen.bmj.com/site/about/guidelines.xhtml

first hospitalisation due to schizophrenia and schizoaffective disorder: Observational follow-up study. *British Medical Journal*. https://doi.org/10.1136/bmj.38881.382755.2F

Vancampfort, D., Stubbs, B., Mitchell, A. J., De Hert, M., Wampers, M., Ward, P. B., Rosenbaum, S., & Correll, C. U. (2015). Risk of metabolic syndrome and its components in people with schizophrenia and related psychotic disorders, bipolar disorder and major depressive disorder: A systematic review and meta-analysis. *World Psychiatry*, *14*(3), 339–347. https://doi.org/10.1002/wps.20252

Verma, S., Al-Omran, M., Leiter, L. A., Mazer, C. D., Rasmussen, S., Saevereid, H. A., Sejersten Ripa, M., & Bonaca, M. P. (2022). Cardiovascular efficacy of liraglutide and semaglutide in individuals with diabetes and peripheral artery disease. *Diabetes, Obesity and Metabolism*, *24*(7), 1288–1299. https://doi.org/10.1111/dom.14700

Ware, J. E. J., & Sherbourne, C. D. (1992). The MOS 36-item short-form health survey (SF-36). I. Conceptual framework and item selection. *Medical Care*, *30*(6), 473–483.

Weiden, P. J., Mackell, J. A., & McDonnell, D. D. (2004). Obesity as a risk factor for antipsychotic noncompliance. *Schizophrenia Research*, *66*(1), 51–57. https://doi.org/10.1016/S0920-9964(02)00498-X

Westman, J., Eriksson, S. V., Gissler, M., Hällgren, J., Prieto, M. L., Bobo, W. V., Frye, M. A., Erlinge, D., Alfredsson, L., & Ösby, U. (2018). Increased cardiovascular mortality in people with schizophrenia: A 24-year national register study. *Epidemiology and Psychiatric Sciences*. https://doi.org/10.1017/S2045796017000166

Zheng, W., Li, X.-B., Tang, Y.-L., Xiang, Y.-Q., Wang, C.-Y., & de Leon, J. (2015). Metformin for Weight Gain and Metabolic Abnormalities Associated With Antipsychotic Treatment: Meta-Analysis of Randomized Placebo-Controlled Trials. *Journal of Clinical Psychopharmacology*, *35*(5), 499–509. https://doi.org/10.1097/JCP.0000000000000392

Østergaard SD, Foldager L, Mors O, Bech P, Correll CU. The Validity and Sensitivity of PANSS-6 in the Clinical

For peer review only - http://bmjopen.bmj.com/site/about/guidelines.xhtml

Antipsychotic Trials of Intervention Effectiveness (CATIE) Study. Schizophr Bull. 2018 Feb 15;44(2):453-462.

For peer review only

For peer review only - http://bmjopen.bmj.com/site/about/guidelines.xhtml

**Table 1**
**Inclusion criteria**
• Diagnosed with schizophrenia spectrum disorder (ICD10 codes DF20, DF21 or DF25)
• Age between 18 and 60 years (both included)
• Approved contraception for female participants
• Treated by one of the community mental health centers and general practitioners in the Region of Southern Denmark or Zealand
• Antipsychotic SGA treatment for at least 6 months
• Stable co-medication for at least 1 month
• HbA1c between 39-47 mmol/mol (both included). Two measurements with ≥3 month interval are required to confirm prediabetes. The first measurement is identified and obtained from patient journals, the second prior to enrolment
• BMI ≥27 kg/m^2. Two weights with ≥3 month interval are required to confirm obesity
• Capable of providing informed oral and written consent
**Exclusion criteria**
• Diagnosis of diabetes (T1D or T2D) or a HbA1c >47 mmol/mol
• Active malignant disease within the last 5 years

For peer review only - http://bmjopen.bmj.com/site/about/guidelines.xhtml

• Pregnancy or breast feeding
• A reported high consumption of alcohol or severe substance abuse
• Unwillingness to allow home visits by a study nurse
• Significant somatic disease: 1) end-stage renal failure (eGFR <15 ml/min); 2) elevated liver function tests (liver transaminases >2 times upper normal limit); 3) history of acute or chronic pancreatitis; 4) heart failure (NYHA class IV) or unstable angina pectoris or myocardial infarction with the last 6 months; 5) uncontrolled hypertension (systolic blood pressure >180 mm Hg, diastolic blood pressure >100 mm Hg)
• Previous treatment with study drug or use of other weight reducing drugs within last 6 month
• Participation in other drug trials
• Treatment with drugs approved for overt diabetes type 2 except metformin.
• Circumstances that the investigator believes will interfere with the trial

For peer review only - http://bmjopen.bmj.com/site/about/guidelines.xhtml

CONSORT Flow Diagram

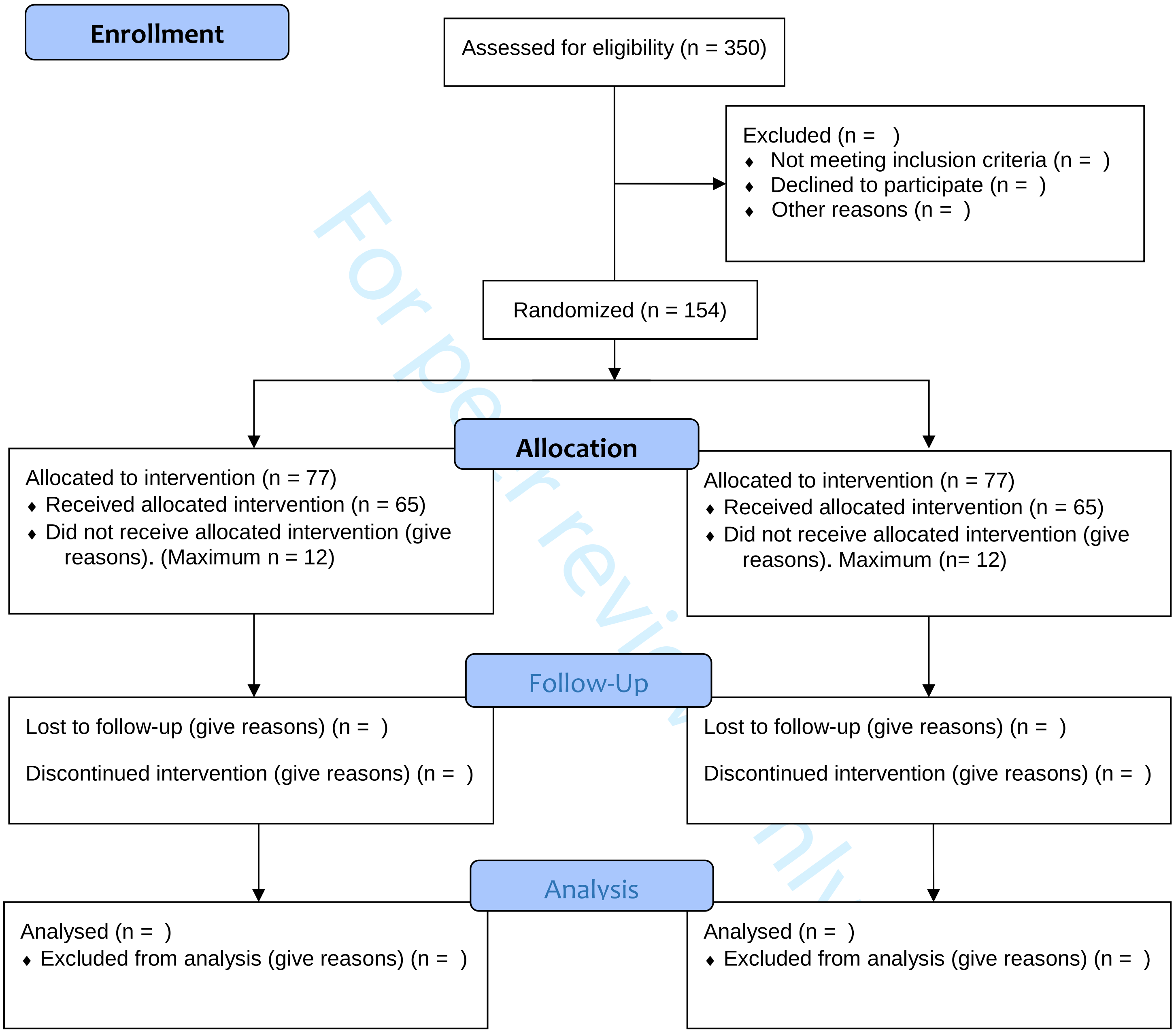

For peer review only - http://bmjopen.bmj.com/site/about/guidelines.xhtml

Table 1.2											
HISTORI	Investigator inclusion	Patient acceptance to participate	Week	Week	Week	Week	Week	Week	Week	Week	Week
			1	5	9	15	19	23	28	30	36
	Physical appearance of patients at hospitals	X				X				X	
			Weekly injection of Semaglutide / placebo								
Consent approval	X										
Baseline number		X									
Evaluation of medical history	X	X									
In- and Exclusion criteria	X	X									
Randomization		X									
Physical examination		X									
Blood pressure	X		X			X				X	
Weight/BMI	X		X			X				X	
Full biochemical screening		X								X	
Safety blood test	X					X					
PET CT scan		Scheduled							Scheduled		
PANNS-6, SF-36v2, IWQOL-Lite		X				X				X	
MARS, SIMPAQ, and EQ-5D		X	X	X	X	X	X	X	X	X	
Cardiac autonomic neuropathy (VAGUS®)		X				X				X	

For peer review only - http://bmjopen.bmj.com/site/about/guidelines.xhtml

**Adjustment of Semaglutide/placebo doses**				**X**	**X**						
**Semaglutide 0.25 mg**			**X**								
**Semaglutide 0.50 mg**				**X**							
**Semaglutide 1.00 mg per week**					**X**	**X**	**X**	**X**	**X**	**X**	
**End of study evaluation**											**X**

For peer review only

For peer review only - http://bmjopen.bmj.com/site/about/guidelines.xhtml

Reporting checklist for protocol of a clinical trial.

Based on the SPIRIT guidelines.

Instructions to authors

Complete this checklist by entering the page numbers from your manuscript where readers will find each of the items listed below.

Your article may not currently address all the items on the checklist. Please modify your text to include the missing information. If you are certain that an item does not apply, please write "n/a" and provide a short explanation.

Upload your completed checklist as an extra file when you submit to a journal.

In your methods section, say that you used the SPIRITreporting guidelines, and cite them as:

Chan A-W, Tetzlaff JM, Gøtzsche PC, Altman DG, Mann H, Berlin J, Dickersin K, Hróbjartsson A, Schulz KF, Parulekar WR, Krleža-Jerić K, Laupacis A, Moher D. SPIRIT 2013 Explanation and Elaboration: Guidance for protocols of clinical trials. BMJ. 2013;346:e7586

		Reporting Item	Page Number
**Administrative information**			
Title	#1	Descriptive title identifying the study design, population, interventions, and, if applicable, trial acronym	1

For peer review only

Trial registration	#2a	Trial identifier and registry name. If not yet registered, name of intended registry	3
Trial registration: data set	#2b	All items from the World Health Organization Trial Registration Data Set	3
Protocol version	#3	Date and version identifier	3
Funding	#4	Sources and types of financial, material, and other support	17-18
Roles and responsibilities: contributorship	#5a	Names, affiliations, and roles of protocol contributors	18
Roles and responsibilities: sponsor contact information	#5b	Name and contact information for the trial sponsor	18
Roles and responsibilities: sponsor and funder	#5c	Role of study sponsor and funders, if any, in study design; collection, management, analysis, and interpretation of data; writing of the report; and the decision to submit the report for publication, including whether they will have ultimate authority over any of these activities	18
Roles and responsibilities: committees	#5d	Composition, roles, and responsibilities of the coordinating centre, steering committee, endpoint adjudication committee, data management team, and	18-20

For peer review only

		other individuals or groups overseeing the trial, if applicable (see Item 21a for data monitoring committee)	
**Introduction**			
Background and rationale	#6a	Description of research question and justification for undertaking the trial, including summary of relevant studies (published and unpublished) examining benefits and harms for each intervention	4
Background and rationale: choice of comparators	#6b	Explanation for choice of comparators	4-6
Objectives	#7	Specific objectives or hypotheses	6
Trial design	#8	Description of trial design including type of trial (eg, parallel group, crossover, factorial, single group), allocation ratio, and framework (eg, superiority, equivalence, non-inferiority, exploratory)	7
**Methods: Participants, interventions, and outcomes**			
Study setting	#9	Description of study settings (eg, community clinic, academic hospital) and list of countries where data will be collected. Reference to where list of study sites can be obtained	7

For peer review only

Eligibility criteria	#10	Inclusion and exclusion criteria for participants. If applicable, eligibility criteria for study centres and individuals who will perform the interventions (eg, surgeons, psychotherapists)	7
Interventions: description	#11a	Interventions for each group with sufficient detail to allow replication, including how and when they will be administered	7-9
Interventions: modifications	#11b	Criteria for discontinuing or modifying allocated interventions for a given trial participant (eg, drug dose change in response to harms, participant request, or improving / worsening disease)	11-12
Interventions: adherance	#11c	Strategies to improve adherence to intervention protocols, and any procedures for monitoring adherence (eg, drug tablet return; laboratory tests)	8
Interventions: concomitant care	#11d	Relevant concomitant care and interventions that are permitted or prohibited during the trial	9
Outcomes	#12	Primary, secondary, and other outcomes, including the specific measurement variable (eg, systolic blood pressure), analysis metric (eg, change from baseline, final value, time to event), method of aggregation (eg, median, proportion), and time point for each outcome. Explanation of the clinical relevance of chosen efficacy and harm outcomes is strongly recommended	9-11

For peer review only

Participant timeline	#13	Time schedule of enrolment, interventions (including any run-ins and washouts), assessments, and visits for participants. A schematic diagram is highly recommended (see Figure)	Table 1.2
Sample size	#14	Estimated number of participants needed to achieve study objectives and how it was determined, including clinical and statistical assumptions supporting any sample size calculations	12
Recruitment	#15	Strategies for achieving adequate participant enrolment to reach target sample size	7
**Methods: Assignment of interventions (for controlled trials)**			
Allocation: sequence generation	#16a	Method of generating the allocation sequence (eg, computer-generated random numbers), and list of any factors for stratification. To reduce predictability of a random sequence, details of any planned restriction (eg, blocking) should be provided in a separate document that is unavailable to those who enrol participants or assign interventions	7
Allocation concealment mechanism	#16b	Mechanism of implementing the allocation sequence (eg, central telephone; sequentially numbered, opaque,	8

For peer review only

		sealed envelopes), describing any steps to conceal the sequence until interventions are assigned	
Allocation: implementation	#16c	Who will generate the allocation sequence, who will enrol participants, and who will assign participants to interventions	8
Blinding (masking)	#17a	Who will be blinded after assignment to interventions (eg, trial participants, care providers, outcome assessors, data analysts), and how	7
Blinding (masking): emergency unblinding	#17b	If blinded, circumstances under which unblinding is permissible, and procedure for revealing a participant's allocated intervention during the trial	12
**Methods: Data collection, management, and analysis**			
Data collection plan	#18a	Plans for assessment and collection of outcome, baseline, and other trial data, including any related processes to promote data quality (eg, duplicate measurements, training of assessors) and a description of study instruments (eg, questionnaires, laboratory tests) along with their reliability and validity, if known. Reference to where data collection forms can be found, if not in the protocol	9

For peer review only

Data collection plan: retention	#18b	Plans to promote participant retention and complete follow-up, including list of any outcome data to be collected for participants who discontinue or deviate from intervention protocols	table 1.2
Data management	#19	Plans for data entry, coding, security, and storage, including any related processes to promote data quality (eg, double data entry; range checks for data values). Reference to where details of data management procedures can be found, if not in the protocol	7
Statistics: outcomes	#20a	Statistical methods for analysing primary and secondary outcomes. Reference to where other details of the statistical analysis plan can be found, if not in the protocol	12
Statistics: additional analyses	#20b	Methods for any additional analyses (eg, subgroup and adjusted analyses)	12
Statistics: analysis population and missing data	#20c	Definition of analysis population relating to protocol non-adherence (eg, as randomised analysis), and any statistical methods to handle missing data (eg, multiple imputation)	12-14

Methods: Monitoring

Data monitoring: formal committee	#21a	Composition of data monitoring committee (DMC); summary of its role and reporting structure; statement of whether it is independent from the sponsor and competing interests; and reference to where further	14

For peer review only

		details about its charter can be found, if not in the protocol. Alternatively, an explanation of why a DMC is not needed	
Data monitoring: interim analysis	#21b	Description of any interim analyses and stopping guidelines, including who will have access to these interim results and make the final decision to terminate the trial	13-14
Harms	#22	Plans for collecting, assessing, reporting, and managing solicited and spontaneously reported adverse events and other unintended effects of trial interventions or trial conduct	11
Auditing	#23	Frequency and procedures for auditing trial conduct, if any, and whether the process will be independent from investigators and the sponsor	11
**Ethics and dissemination**			
Research ethics approval	#24	Plans for seeking research ethics committee / institutional review board (REC / IRB) approval	14
Protocol amendments	#25	Plans for communicating important protocol modifications (eg, changes to eligibility criteria, outcomes, analyses) to relevant parties (eg, investigators, REC / IRBs, trial participants, trial registries, journals, regulators)	14

For peer review only

Consent or assent	#26a	Who will obtain informed consent or assent from potential trial participants or authorised surrogates, and how (see Item 32)	7
Consent or assent: ancillary studies	#26b	Additional consent provisions for collection and use of participant data and biological specimens in ancillary studies, if applicable	7
Confidentiality	#27	How personal information about potential and enrolled participants will be collected, shared, and maintained in order to protect confidentiality before, during, and after the trial	15
Declaration of interests	#28	Financial and other competing interests for principal investigators for the overall trial and each study site	17
Data access	#29	Statement of who will have access to the final trial dataset, and disclosure of contractual agreements that limit such access for investigators	19
Ancillary and post trial care	#30	Provisions, if any, for ancillary and post-trial care, and for compensation to those who suffer harm from trial participation	19
Dissemination policy: trial results	#31a	Plans for investigators and sponsor to communicate trial results to participants, healthcare professionals, the public, and other relevant groups (eg, via publication, reporting in results databases, or other data sharing arrangements), including any publication restrictions	18

For peer review only

Dissemination policy: authorship	#31b	Authorship eligibility guidelines and any intended use of professional writers	18
Dissemination policy: reproducible research	#31c	Plans, if any, for granting public access to the full protocol, participant-level dataset, and statistical code	15

Appendices

Informed consent materials	#32	Model consent form and other related documentation given to participants and authorised surrogates	1
Biological specimens	#33	Plans for collection, laboratory evaluation, and storage of biological specimens for genetic or molecular analysis in the current trial and for future use in ancillary studies, if applicable	n/a

The SPIRIT Explanation and Elaboration paper is distributed under the terms of the Creative Commons Attribution License CC-BY-NC. This checklist was completed on 07. June 2023 using https://www.goodreports.org/, a tool made by the EQUATOR Network in collaboration with Penelope.ai

For peer review only

BMJ Open

Home based Intervention with Semaglutide Treatment of Neuroleptic-Related Prediabetes (HISTORI): protocol describing a prospective, randomized, placebo controlled and double blinded multicenter trial

Journal:	*BMJ Open*
Manuscript ID	bmjopen-2023-077173.R1
Article Type:	Protocol
Date Submitted by the Author:	16-Feb-2024
Complete List of Authors:	Ganeshalingam, Ashok; Odense Universitetshospital, Endocrine Research Unit, Department of Endocrinology, Odense University Hospital & Department of Clinical Research, Faculty of Health, ; Lillebaelt Hospital - University Hospital of Southern Denmark, Department of Internal Medicine<br>Uhrenholt, Nicolai; Psykiatrien Region Sjælland Psykiatrien Vest<br>Arnfred, Sidse; University of Copenhagen,<br>Gæde, Peter; Slagelse Hospital, Department of Cardiology and Endocrinology<br>Bilenberg, Niels; University of Southern Denmark, Department of Clinical Research, Faculty of Health Sciences, University of Southern Denmark, Odense, Denmark.; University of Southern Denmark, Department of Child and Adolescent Mental Health Odense, Mental Health Services in the Region of Southern Denmark, University of Southern Denmark, Odense, Denmark.<br>Frystyk, Jan; Odense University Hospital Department of Endocrinology
**Primary Subject Heading**:	Mental health
Secondary Subject Heading:	Diabetes and endocrinology, Mental health, Cardiovascular medicine, Medical management
Keywords:	PREVENTIVE MEDICINE, General diabetes < DIABETES & ENDOCRINOLOGY, Schizophrenia & psychotic disorders < PSYCHIATRY, Primary Prevention, Obesity

SCHOLARONE™
Manuscripts

HISTORI

Home based Intervention with Semaglutide Treatment of Neuroleptic-Related Prediabetes (HISTORI): protocol describing a prospective, randomized, placebo controlled and double blinded multicenter trial

A. Ganeshalingam [1,2,3,6], N. Uhrenholt [4], S. Arnfred [4], P. Gæde [5], N. Bilenberg [3], J. Frystyk [1]

1. Endocrine Research Unit, Department of Endocrinology, Odense University Hospital & Department of Clinical Research, Faculty of Health, University of Southern Denmark, Odense, Denmark
2. Department of Internal Medicine, Lillebaelt Hospital, University Hospital of Southern Denmark, Kolding, Denmark
3. Mental Health Services in the Region of Southern Denmark, Child and Adolescent Psychiatry Odense, Odense C, Denmark
4. Psychiatry West, Region Zealand, Research Unit for Psychotherapy and Psychopathology, Slagelse, Denmark
5. Næstved, Slagelse og Ringsted Hospital, Department of cardiology and endocrinology, Slagelse, Denmark
6. Steno Diabetes Center Odense, Odense University Hospital, Denmark

For peer review only

Running title: Patients with prediabetes and schizophrenia on Semaglutide

Keywords

Words in main text: 4670
Number of Tables: 2
Number of Figures: 1
Number of References: 55

Corresponding author

Dr. Ashok Ganeshalingam

27 Department of Endocrinology
28 Odense University Hospital
29 Kløvervænget 6, entrance 93
30 DK-5000 Odense C, Denmark.
31 E-mail: Ashok.Ganeshalingam@rsyd.dk

32

33 ABSTRACT

34 Introduction: Subjects with schizophrenia have a 2 to 3 fold higher mortality rate than the general 35 population, and a reduced life expectancy of 10 to 20 years. Approximately 1/3 of this excess mortality has 36 been attributed to obesity-related type 2 diabetes (T2D) and to cardiovascular disease (CVD). Glucagon-like 37 peptide-1 (GLP-1) analogs increase satiety and delay gastric emptying, thereby reducing food intake and 38 weight. GLP-1 analogs also exert beneficial effects on cardiovascular outcomes in high-risk T2D patients.

39 Our aim is to investigate whether 30 weeks add-on treatment with the GLP-1 analog Semaglutide can 40 reduce HbA1c sufficiently to reverse prediabetes and the metabolic syndrome (MetS) in overweight 41 schizophrenic patients.

42 Methods and analysis: We will perform a 30 week, two-armed, multi-center, superiority, double-blinded, 43 randomized trial investigating the effect of weekly injections of Semaglutide vs placebo in mental health 44 facilities in Region of Southern Denmark and Region of Zealand, Denmark. In total 154 adults with 45 schizophrenia spectrum disease, aged 18 - 60 years treated with second generation anti-psychotic (SGA) 46 treatment, HbA1c 39-47 mmol/mol and BMI > 27 kg/m^2 will be randomized to injections of 1.0 mg 47 Semaglutide or placebo. The primary outcome is changes in HbA1c. Secondary outcomes encompass 48 metabolic measures, psychotic symptoms and QOL. Exploratory outcomes encompass insulin sensitivity, 49 cardiovascular risk profile, , medication adherence, general well-being and physical activity.

For peer review only - http://bmjopen.bmj.com/site/about/guidelines.xhtml

Ethics and dissemination: This study will be carried out in accordance with the Helsinki Declaration and Good Clinical Practice guidelines. This research has obtained approval from both the Danish Medicines Agency and The Regional Committees on Health Research Ethics for Southern Denmark.

Trial Registration number NCT05193578 European Clinical Trials Database Number (EudraCT) 2020-004374-22, Regional Ethical Committee number S-20200182

Based on protocol version 3.2, approved 19th April 2023.

Strengths and limitations of this study

- A 30-week trial designed with feedback from patients and relatives, conducted as a double-blinded, randomized, parallel-group, and placebo-controlled study, adhering to good clinical practice standards and monitored across multiple centers.

- The integration of endocrinology and psychiatry in our study holds significant value as it provides a holistic understanding of the intricate interplay between hormonal regulation and mental health, offering potential insights into innovative approaches for comprehensive patient care.

- This study employs a unique home-based intervention approach, facilitating data collection within participants' natural environments, enabling individuals experiencing severe symptoms of schizophrenia to engage in the study.

- Despite the significance of the study, it is important to note that the 30-week trial duration may impose limitations on fully exploring the comprehensive potential of GLP-1 receptor agonists.

For peer review only - http://bmjopen.bmj.com/site/about/guidelines.xhtml

80 **BACKGROUND AND SIGNIFICANCE OF HISTORI**

81 The life expectancy of subjects with schizophrenia spectrum disorders (hereafter schizophrenia) is reduced
82 by 10 to 20 years when compared to the general population[1][2][3][4]. The excess mortality is partly
83 explained by a 2 to 3 fold increase in cardiovascular disease (CVD) [5] [6]. A nationwide register study from
84 Sweden, including more than 46,000 subjects with schizophrenia showed that CVD was the leading cause of
85 death, causing more deaths than suicide, and that death from CVD occurred 10 years earlier in schizophrenic
86 subjects than in the general population [6].

87 Another factor predisposing to the reduced life expectancy is type 2 diabetes (T2D), which develops in 10 to
88 15 % of all patients with schizophrenia [7]. Compared to the general population, patients with schizophrenia
89 are more likely to have diabetes, hypertension, dyslipidemia, to be overweight and to smoke [8]. Indeed, a
90 third of patients with schizophrenia have been reported to suffer from the metabolic syndrome (MetS) [9].

91 Antipsychotic treatment is vital to reduce schizophrenia related symptoms [10] and treatment with second
92 generation anti-psychotic (SGA) drugs has demonstrated beneficial effects on mortality, suicide rates and
93 hospitalizations [11]. However, SGA treatment comes with unfortunate side effects, which typically include
94 increased risks of developing obesity, MetS and prediabetes [12] [13]. Furthermore, SGA treatment *per se*
95 leads to impaired glucose tolerance, diabetes, hyperlipidemia and CVD [14] [15].

96 Gaining weight is associated with a reduced quality of life (QoL) in people with schizophrenia [16–21]. In
97 addition, weight gain and MetS are among the most frequently reported reasons for discontinuation of
98 treatment in this group[22]. Indeed, in a cohort of 304 subjects with schizophrenia, 94% reported distress
99 over body weight, and those who were obese appeared to be more than twice as likely to be non-adherent
100 compared to those with a BMI below 25 kg/m^2 [23]. Therefore, it may be speculated that weight-reducing
101 treatments may lead to an increased adherence to SGA treatment. However, to the best of our knowledge

For peer review only - http://bmjopen.bmj.com/site/about/guidelines.xhtml

102 the association between a Glucagon-like Peptide 1 (GLP-1) agonist-induced weight reduction, the severity of
103 psychotic symptoms, and the ability to adhere to SGA treatment has never been investigated in a randomized
104 clinical trial (RCT).

105 Lifestyle and environmental factors such as smoking, poor diet and an insufficient capacity to exercise
106 contribute to the risk of CVD [14]. Behavioral lifestyle interventions aiming at improving diet and physical
107 activity have indeed shown short-term beneficial effects, but long term effects diminish over time compared
108 to standard care[24] [25]. When lifestyle programs fail to reduce the increased risk of CVD and T2D,
109 metformin is standard treatment according to NICE guidelines (NICE 2012). In a meta-analysis of 19 RCTs
110 (N=1,279) the addition of metformin to ongoing anti-psychotic treatment for an average of 3 to 4 months in
111 subjects with severe mental illness reduced body weight but only by 0.61 kg [26]. Therefore, alternative
112 weight reducing drugs are required, e.g. GLP-1 analogs.

113 The potential of GLP-1 analogs to improve the metabolic profile of patients with schizophrenia was recently
114 successfully tested. In a randomized controlled trial, including 97 patients (placebo/active comparator:
115 50/47) with schizophrenia, prediabetes and a BMI ≥27 kg/m^2, 16 weeks of Liraglutide (1.8 mg Victoza® daily)
116 reduced body weight, waist circumference and abdominal fat mass, LDL cholesterol, glucose levels and
117 insulin resistance, without any worsening in the psychiatric disorder [27]. The beneficial effect of GLP-1
118 analog treatment in SGA-treated patients was later re-confirmed in a 2018 meta-analysis which, however,
119 only included two additional and smaller trials of Exanatide (Bydureon®), and only one of these studies was
120 placebo-controlled [28]. Nevertheless, the review concluded that GLP-1 analog treatment is effective and
121 tolerable for SGA-related weight gain, but that more studies are required[29] [30].

122 Today, liraglutide has been replaced by semaglutide, which is more efficient as a weight losing agent in obese
123 subjects [31]. Furthermore, semaglutide is easier to administer than liraglutide as semaglutide requires
124 weekly and not daily injections. This may be important as it opens for the possibility of administering

For peer review only - http://bmjopen.bmj.com/site/about/guidelines.xhtml

semaglutide by mobile nurses performing weekly house calls, thereby improving adherence to treatment, which is known to be reduced in patients with schizophrenia [32]. Importantly, in non-diabetic obese subjects, semaglutide is able to improve various cardio metabolic risk factors [33] and to prevent new cardiovascular events (death from cardiovascular causes as well as nonfatal myocardial infarction and stroke) in subjects with preexisting cardiovascular disease [34]. Therefore, semaglutide appears as a suitable treatment for obese schizophrenic patients.

Aim and hypothesis

The aim of this study is to perform a randomized placebo-controlled double-blinded trial of Semaglutide 1.0 mg in comparison to placebo in patients with schizophrenia spectrum disorders (SZD), i.e., either schizophrenia, schizotypal disorder or schizoaffective disorder, and comorbid obesity and prediabetes. (The study was planned and initiated prior to the release of Semaglutide 2.4 mg; Wegovy®).

We hypothesize that administration of Semaglutide at a dose of 1.0 mg administered subcutaneously once a week for 30 weeks improves HbA1c (primary outcome) in SGA-treated adults with SZD suffering from prediabetes (HbA1c between 37 and 47 mmol/mol) and a BMI ≥27 kg/m^2, when compared to placebo. Additionally, that Semaglutide improves the following secondary endpoints: weight, schizophrenic symptoms (PANSS-6), QoL-related measures, patient-related outcomes (PRO data) and the cardiometabolic risk factor profile.

METHOD

Design. The current trial is an investigator-initiated, double-blinded, parallel, superiority, multi-center RCT with a 1:1 randomization. In total, we will include 154 patients recruited from two Danish regional mental health services (MHS). A CONSORT diagram is provided in **Figure 1**. Data management is purely digital and

For peer review only - http://bmjopen.bmj.com/site/about/guidelines.xhtml

managed by Open Patient data Explorative Network (OPEN), Odense University Hospital, Denmark, using REDCAP ©.

SETTING

The study will recruit patients from community mental health centers and private practitioners in the Region of Southern Denmark and the Region of Zealand, Denmark.

PARTICIPANTS

Patients with a clinical diagnosis of either schizophrenia, schizotypal disorder or schizoaffective disorder (ICD-10 diagnoses: F20.x; F21; F25.x), age between 18 and 60 years receiving treatment in the regional mental health care of participating centers, or by private practice, and fulfilling criteria as listed in **Table 1**.

PROCEDURES

Recruitment

Patients fulfilling inclusion criteria will be invited from community mental health center clinics and private practices in the Region of Southern Denmark and the Region of Zealand. According to Danish clinical guidelines, patients treated with neuroleptics (almost exclusively SGAs) require minimum one annual laboratory test and clinical evaluation to test for the presence of the MetS. Eligible subjects accepting to participate will be invited to screening visits and allocated to treatment with placebo or Semaglutide at a dose of 1.0 mg administered subcutaneously once a week by blinded randomization, if all inclusion criteria and none of the exclusion criteria are met. The PhD students will be in charge of recruitment participants, obtaining consent to participation, initiation of medication and execution of the trial including inclusion visits and follow-up visits.

For peer review only - http://bmjopen.bmj.com/site/about/guidelines.xhtml

167 ## Randomization and concealment

168 A total of 154 participants will be enrolled and randomized 1:1 to Semaglutide or placebo. Block
169 randomization including blocks of either 4 or 6 randomization numbers will be used, and the randomizations
170 list will be provided by the manufacturer of Semaglutide and placebo (Novo Nordisk A/S, Novo Allé, Bagsværd
171 DK-2880 Denmark). Randomization will be performed using REDCAP ©.

172 ### *Mobile project nurses*

173 The presence of schizophrenia impacts adherence and accordingly, adherence to study medication may be
174 a challenge [35]. Therefore, we perform data collection at home by mobile study nurses to ensure optimal
175 adherence and to minimize discomfort for the participants.

176 The study nurse will visit the subjects in their homes to administer injection and teach eligible subjects to
177 self-inject under supervision. **Thus, apart from the enrolment, we plan for a home-based study.** If some
178 patients are able to adhere to treatment and to self-inject, weekly visits may not be necessary. Thus, the
179 frequency of home visits may be downscaled to involve monthly collection of data only. All data collection
180 is allocated to study nurses and the PhD-students, and the participants will maintain management-as-usual
181 (MAU) in the respective clinics from where they are recruited.

182 ## Interventions

183 The intervention consists of 30 weeks of treatment with once-weekly subcutaneous injections of either
184 Semaglutide (1.34 mg/ml; 1.0 mg) or placebo. The current dose adjustment guidelines for Semaglutide will
185 be followed (i.e., starting at 0.25 mg weekly for 4 weeks, then 0.5 mg weekly for 4 weeks, and finally 1 mg
186 or highest tolerated weekly dose until the end of intervention) or placebo (corresponding volume).
187 Subjects, who develops side effects when reaching maximum dose, will be reduced to the highest possible
188 tolerated dose of Semaglutide or placebo for the rest of the study.

For peer review only - http://bmjopen.bmj.com/site/about/guidelines.xhtml

For peer review only

Relevant concomitant care and interventions are permitted during the trial.

Patient and Public Involvement

The project's design was developed in collaboration with individuals diagnosed with schizophrenia and their family members. Their valuable contributions have provided insights into the possibilities of conducting examinations within a home-based setting.

Ethics and dissemination

This research has obtained approval from both the Danish Medicines Agency and The Regional Committees on Health Research Ethics for Southern Denmark

OUTCOMES

Primary outcome

Absolute reduction in HbA1c from baseline to end of treatment (30 weeks).

Secondary outcomes

During the treatment and after 30 weeks of treatment:

a) **Metabolic measures**: BMI, blood pressure, triglyceride, cholesterol (HDL and LDL), waist circumference and HOMA-estimates based on fasting insulin and plasma glucose.
Blood test will be taken at the nearest hospital prior to inclusion, at the inclusion, at 15 weeks and at end of study. BMI, waist circumference and blood pressure will be measured at week 1, 15 and 30.

b) **Positive and Negative Symptom Scale 6 (PANSS-6):** PANSS is an operationalized rating of positive and negative symptoms, in people with schizophrenia, which is sensitive to change [36]. PANSS-6 is a shorter version of the original 30-item version. It is validated and translated into Danish and has

For peer review only - http://bmjopen.bmj.com/site/about/guidelines.xhtml

proven a good level of inter-rater reliability when rated based on the Simplified Negative and Positive Symptoms Interview (SNAPSI) [37,38] .PANSS-6 interview will be performed at baseline, and after 15 and 30 weeks.

c) **Short Form Survey (SF-36)** is a well-researched, self-reported measure of health. It comprises 36 questions in 8 different domains. The survey will be applied in Danish, using the latest updated version 2 (SF-36v2) [39]. SF-36v2 will be measured at baseline, and after 15 and 30 weeks.

Exploratory outcomes:

a) **Cardiovascular risk markers:** cardiovascular autonomic neuropathy (CAN) (Vagus TM®), and circulating levels of cardiovascular risk markers. CAN is considered a serious complication of diabetes, being associated with a 5-fold increased risk of CVD [40]. Of interest to this study, CAN may be present prior to the development of diabetes [41] and it can be improved by weight loss [42]. As CAN may be measured by a hand held apparatus (Vagus TM®), measurements are easily performed in the homes of the enrolled patients. CAN will be measured at inclusion, at 15 weeks and at end of study.

Moreover, the CAN results will be juxtaposed with various cardio-metabolic risk markers, including proteins belonging to the GDF-family, activin, follistatin, the insulin-like growth factor (IGF)-system and its regulating proteins and enzymes, and the adipokines leptin and adiponectin. All these data are explorative tertiary outcomes.

b) **Pro-atherosclerotic changes:** we want to perform a pilot study in a subsample of 20 participants from placebo vs. 20 participants from the Semaglutide group, focusing on pre-symptomatic atherosclerosis assessed by positron emission tomography/computed tomography (PET/CT) using 18F-sodium fluoride (NaF) as tracer. NaF-PET/CT is a novel way of detecting and measuring calcification in major arteries, potentially years or decades before manifest arterial wall calcification

For peer review only - http://bmjopen.bmj.com/site/about/guidelines.xhtml

becomes detectable by cardiac CT [43,44]. We plan to perform two NaFPET/CT acquisitions, i.e. at baseline and after 30 weeks treatment. Based on our experience, inclusion of 20 placebo- and 20 Semaglutide-treated subjects is suitable to perform the pilot study. We hypothesize a ≥10% decrease in global disease score [45] as measured by NaF uptake in the heart and/or thoracic aorta, will be present after 30 weeks treatment**.**

c) **Impact of Weight on Quality of Life-Lite (IWQOL-Lite):** IWQOL-Lite is a reliable and valid self-report measure for assessing weight-related QoL in people with schizophrenia [46]. It provides a total score plus scores on five domains (physical function, self-esteem, sexual life, public distress, and work). It is translated into 83 languages, including Danish. IWQOL-Lite will be measured at baseline, and after 15 and 30 weeks.

d) **Medication Adherence Rating Scale (MARS):** MARS is a validated and reliable measure of adherence for psychoactive medication. MARS constitutes 10 items rating the medical adherence [47] [48]. MARS will be measured at baseline, and at weeks 5, 9, 15, 19, 23, 28 and 30.

e) **The Simple Physical Activity Questionnaire (SIMPAQ):** SIMPAQ has shown good test-retest reliability and has been validated against measures from Actigraph accelerometers [49]. SIMPAQ will be measured at baseline, and at weeks 5, 9, 15, 19, 23, 28 and 30.

f) **The EuroQol five-dimensional (EQ-5D)** questionnaire is a widely applied instrument for the measurement and cost-benefit evaluation of health interventions [50]. EQ-5D will be measured at baseline, and at weeks 5, 9, 15, 19, 23, 28 and 30.

SAFETY ASSESSMENTS

Adverse reactions will be evaluated weekly. At every 4-week visit, vital parameters will be recorded, including blood pressure, body weight and waist circumference. The safety parameters in the study include

For peer review only

For peer review only - http://bmjopen.bmj.com/site/about/guidelines.xhtml

256 adverse reactions, vital parameters and liver and kidney function, which will be analyzed at baseline, week
257 15 and week 30. If any side effects or changes in biochemical measures are observed, the trial physician or
258 the physician responsible for the test will be informed.

259 All safety parameters will be recorded in an electronic case report form (eCRF) as soon as they have either
260 been measured or reported by the study participant. The study will be monitored by good clinical practice
261 (GCP) and follow current GCP guidelines. The study protocol has been reviewed by GCP prior to inclusion of
262 first participant, and subsequently, the study is monitored by GCP throughout the study period.

263 After drug administration, all potential side effects and other adverse events (AEs) observed will be
264 reported in the eCRF. Semaglutide half-life is one week and thus expected to be completely eliminated
265 after six weeks). Consequently, collection of AEs for all patients will be completed six weeks after the final
266 injection by telephone calls. The nature of each AE will be assessed according to the severity and
267 relationship of the experimental medicine or trial procedure, and will be assessed by the primary
268 investigator, who is a physician. If it is considered that the AE is related to the test drug or test procedure, it
269 will be noted as a side effect in the eCRF. All severe adverse events (SAEs) will be reported to the sponsor.
270 Hospital admissions related to schizophrenia as soon as possible, other hospital admissions always within
271 24 hours of it coming to our attention. All severe adverse reactions (SARs) which, regardless of dose, result
272 in death, are life-threatening, result in hospitalization or prolongation of hospital stay in medical or surgical
273 departments, result in significant or persistent disability or incapacity, or lead to congenital anomaly or
274 malformation, will be considered in this trial to be unexpected (SUSAR).

275 Emergency un-blinding will occur if the participants develop adverse events that require knowledge of the
276 treatment, if patient develop sign of overdose or if patient develop conditions that require treatment with
277 the trial drug (Semaglutide) or drugs with potential interactions with trial drug.

For peer review only - http://bmjopen.bmj.com/site/about/guidelines.xhtml

STATISTICAL CONSIDERATIONS

Primary outcomes for the study will be absolute changes in HbA1c. Changes in HbA1c will be evaluated after 15 weeks and after 30 weeks.

Assuming that 30 weeks of Semaglutide reduces HbA1c by 0.2%, and that SD equals 0.35%, **65 subjects in each arm are required to obtain a power of 90%**, using a 2-sided significance level of 5%.

Dropout from trials concerning pharmacological treatment for obesity is non-linear; the dropout from the treatment group will occur earlier due to side effects, while in the placebo group dropouts occur later due to lack of efficacy.

Feasibility

In the SUSTAIN 1 trial (30 weeks of Semaglutide in patients with T2D), the discontinuation of study drug amounted to 13% in those receiving 0.5 mg Semaglutide, 12% in those receiving 1.0 mg Semaglutide, and 11% in placebo-treated subjects [51]. In the 16-week study with Liraglutide in patients with schizophrenia, 23 of 214 patients declined to participate, and 111 patients did not meet inclusion criteria, leaving 103 to be enrolled. Of these, only 7 patients dropped out of the study, leaving an overall success rate of 45 percent [52]. We believe to experience similar rates. Thus, we need to approach approximately 350 patients to enroll 154 patients. Given the number of potentially eligible patients in the two regions (currently estimated to >300 excluding new cases in the age 18-60 years), we believe this is feasible. In the present study, we will follow the participants for 30 weeks. Hence, we predict a higher risk of dropout. Accordingly, we conservatively assume a dropout rate of 15 percent to ensure that the required number of subjects to obtain power are recruited.

For peer review only - http://bmjopen.bmj.com/site/about/guidelines.xhtml

For peer review only

Although this study is not designed as treatment for obesity, we believe the estimation of dropout is reliable as the study focus on the same patient population and the pharmacological profile of the investigated drug is similar to Liraglutide [52].

DATA ANALYSIS

All participants randomized in the study will be included in the assessment of endpoints. All data will be analyzed based on the intention-to-treat population. Clinical results will be presented as mean, least squares mean, least square mean change from baseline or least square difference between groups with SE, SD or two-sided 95% CI as appropriate. Statistical tests will be conducted as two-sided tests with a 5% significance level.

For peer review only

A mixed effects linear regression analysis will be used to compare changes in glucose tolerance, HbA1c, from baseline to 30 weeks follow-up between the intervention and the placebo groups. Missing values will be imputed, primary analysis will be intention-to-treat. Supplementary per protocol analysis will accompany the intention to treat analysis.

All changes in secondary outcomes from baseline to various follow-up times are analyzed using mixed effects linear regression analyses for continuous outcomes and mixed effects logistic regression analyses for categorical outcomes. All analyses on continuous outcomes are adjusted for the baseline value of the outcome, and in case of significant imbalances in baseline patient characteristics between the two randomized groups, exploratory analyses including such variables are conducted. To correct for multiple testing of the secondary outcomes, Holm's sequentially rejective multiple test procedure will be applied. Statistical analyses are performed using Stata software Version 16 (Statacorp, Texas, USA). The hypothesis tests are 2-sided, and the 5% level of significance is considered.

For peer review only - http://bmjopen.bmj.com/site/about/guidelines.xhtml

ETHICAL APPROVAL AND CONSENT TO PARTICIPATE

The project is approved by the Regional Science Ethical Committee for the Region of Southern Denmark and the Danish Medicine Agency. Data processing in connection with the study is reported to the region's list of on-going research projects via the Executive Secretariat at Odense University Hospital (OUH). The study participants will not necessarily be able to benefit from participation in the study. Individual participants may be diagnosed with illness that may require treatment; in that case, they will be referred for relevant treatment. Participants will not have overt diabetes based on HbA1c. Therefore, following national guidelines, participants in this study are not candidates for standard treatment of diabetes and under normal circumstances they would not receive antidiabetic treatment or treatment that would reduce their risk of diabetes. However, Metformin treatment of the MetS is accepted.

Amendments to the study protocol will be made to the Regional Science Ethical Committee for the Region of Southern Denmark and the Danish Medicine Agency.

The study will be conducted in accordance with the Helsinki Declaration with amendments as well as in accordance with the General Data Protection Regulation (GDPR), and the Danish Data Protection. In the case that biological material will be shipped to collaborators abroad, the Danish Protection Agency will be applied and Chapter V of the Danish Protection Act will be obeyed. It is mandatory that the clinical staff involved in the clinical trial has completed an official course in Good Clinical Practice.

TRIAL STATUS

Recruitment opens January 2022 with first patient, first visit (FPFV), whereas last patient, last visit (LPLV) is scheduled for **September 2024.** Analysis of primary endpoints is planned to **March 2025**: Data will be published in EudraCT within 12 months from LPLV. Publication of the final results on primary endpoint is

For peer review only - http://bmjopen.bmj.com/site/about/guidelines.xhtml

For peer review only

expected to be made public in **March 2025**. The results will be disseminated in peer review publications and conference presentations.

For peer review only

For peer review only - http://bmjopen.bmj.com/site/about/guidelines.xhtml

DISCUSSION

Metabolic disturbances, obesity and diabetes development in SGA treated patients are a major problem in the clinical setting, but the underlying mechanism is poorly understood, even though there are theories focusing on an interaction of medication, genes, life style, diet and physical activity [14] [9] [1] .

GLP-1 analogs have shown remarkable effect in the treatment of diabetes by contributing to weight loss, lowering blood glucose levels and improving mortality from CVD [51] [33,34]. This is in contrast to life style interventions with physical activity and diet changes, as such initiatives have failed to induce robust long-term improvements of the metabolic disturbances [53][25][24].

The main focus of this clinical trial is mitigation of metabolic aberrations due to SGA treatment and reversal of prediabetes. Hence, the primary outcome is the absolute reduction in HbA1c rather than weight loss.

Patients with severe mental illness experience many challenges due to schizophrenic symptoms and side effects to SGA treatment [12][13]. Recruitment may be difficult in this patient population, and accordingly, we are collaborating with local psychiatric teams in both Regions and with local consultants to create a secure environment. The study is designed to focus on adherence to study protocol and minimize the patient's burden of study participation. The home visit by study nurses is likely to strengthen protocol adherence by assisting the participants and creating a secure and safe environment, eventually yielding higher reliability of data from interviews and questionnaires.

Semaglutide holds benefits compared to Liraglutide when dealing with patients with weight gain caused by SGAs. Semaglutide requires weekly treatment only, which makes it easier to administer with help from a study-nurse. In addition, the weight reducing effect of Semaglutide is greater than Liraglutide without compromising the cardio-protective effect [54]. Hence, if Semaglutide 1.34 mg/ml has a beneficial effect on

For peer review only - http://bmjopen.bmj.com/site/about/guidelines.xhtml

weight, HbA1c, MetS and QoL, it could potentially be used in patients with schizophrenia in high risk of cardiovascular disease to prevent premature death due to diabetes.

Our study will attempt to mitigate the metabolic side effects of SGA induced overweight in individuals with schizophrenia and prediabetes. Treatment with Semaglutide improves glycemic control, weight and clinical cardiovascular risk factors by reducing numbers of fatal and non-fatal myocardial infarction and non-fatal stroke within 104 weeks [55]. We hypothesize that beneficial pre-clinical events are detectable by NAF uptake using PET-CT scans after 30 weeks of treatment. If so, this may contribute to a better understanding of the nature behind the increased cardiovascular risk within these patients.

We hope that our trial HISTORI will provide insight in how to benefit the cardio-metabolic health and the QoL of schizophrenic patients – adding more and better years - and thereby change the HISTORY of this vulnerable group of patients. Additionally, if successful, HISTORI may pave the way for long-term, large-scale GLP-1 agonist interventions in schizophrenic patients with prediabetes, aiming to reduce the number of hardcore clinical cardiovascular endpoints, and thereby prolonging their life expectancy. Finally, a positive outcome may fuel initiatives to study the ability of GLP-1 agonists to prevent the development of the prediabetes, e.g. by starting treatment shortly after treatment with SGAs are initiated in high-risk patients.

Funding Statement

Sources and types of financial, material, and other support funding for the study has been obtained from private foundations:

Novo Nordisk Foundation: 8,584,959DKK (salary and tuition fee PHD Students and study-nurses, running expenses, biochemical and laboratory procedures, TAP personal, mileage allowance)

For peer review only

Research Grant from Steno Diabetes Center Zealand, Denmark: 660,000 DKK.

Research Grant from Steno Diabetes Center Odense, Denmark: 578,000 DKK.

Annum (Region Zealand) to PhD-student Nicolai Uhrenholt: 20.000 DDK per year for three years (60,000 DKK in total).

Slagelse Puljen, 2020: The Region of Zealand, Denmark: 240,000 DKK

Slagelse Puljen 2022: 150,000 DKK

Region Sjællands Sundhedsvidenskabelige Forskningsfond 2022: 252,631 DKK. Furthermore, Novo Nordisk A/S will provide investigational drug and placebo free of charge for sponsor, but is otherwise not involved in the study. The scientists involved will conduct the experiment out of general scientific interest without personal financial gain. None of the investigators involved have financial interests (including shares, direct employment, members of advisory boards) in the drug company that produces the active drug used in the study. None of the participants in the study will receive any provision for participation. Participants will receive mileage allowances. Any harm as a result of study participation will be covered by the Danish Patient Compensation.

Roles and responsibilities

a. Contributorship statement:

AG, NU, SA, PG, NB, and JF initiated the study design, AG and NU helped with implementation and execution of the study. JF, NB, SA, and PGH are grant holders. JF and NB provided statistical expertise in clinical trial design, AG and NGU are conducting the primary statistical analysis.

For peer review only - http://bmjopen.bmj.com/site/about/guidelines.xhtml

For peer review only

AG made significant contributions to the study protocol in the design and the writing process in with contributions from NGU. All authors contributed to refinement of the study protocol and approved the final manuscript.

Data from this study belongs to the research group behind Project HISTORI and can be accessed after application to the sponsor JF.

Sponsor:

Odense University Hospital

Steno Diabetes Centre Odense / Department of Endocrinology M. J.B. Winsløvvej 4. DK- 5000 Odense, Denmark.

Correspondence:

Jan Frystyk, Clinical Professor, DMsc, Ph.D. Steno Diabetes Centre Odense / Department of Endocrinology M. Odense University Hospital. J.B. Winsløvvej 4. DK- 5000 Odense, Denmark. Email: jan.frystyk@rsyd.dk

Primary Investigator at the Region of Southern Denmark:

Jan Frystyk, Clinical Professor, DMsc, Ph.D. Clinical management of diabetic patients at SDCO; implementation of biomarkers in clinical studies, and PI on clinical studies, hereunder GCP-monitored RCT. Department of Endocrinology M / Steno Diabetes Centre Odense (SDCO). Odense University Hospital. J.B. Winsløvvej 4. DK- 5000 Odense, Denmark.

Primary Investigator at the Region Zealand Community Psychiatry:

Sidse Arnfred, Clinical Professor.

Specialist in family medicine, psychiatry and board certified supervisor in psychotherapy (CBT). Research expertise in schizophrenia, psychopathology and psychophysiology. Experienced PI on RCT's, qualitative studies of user experiences and treatment evaluation. Psykiatrien Region Sjælland, Psykiatrien Vest, Fælledvej 6, bygning 3, 4. sal, DK- 4200 Slagelse, Denmark.

Primary Investigator at the Region of Southern Denmark community Psychiatry

Niels Bilenberg, Clinical Professor, Ph.D. OPUS clinics and community psychiatry centers of Region of Southern. Mental Health Hospital and University Clinic in the Region of Southern Denmark. J.B.Winsløws Vej 28 B, indg. 236, DK-5000 Odense, Denmark.

For peer review only - http://bmjopen.bmj.com/site/about/guidelines.xhtml

For peer review only

Co-investigators:

Peter Gæde, MD, DMSci, Chief Physician, Associate Professor. Department of Steno Diabetes Centre Sjælland. Steno Diabetes Center Sjælland, Akacievej 7, 1.sal. DK-4300 Holbæk, Denmark.

PhD students:

Ashok Ganeshalingam, Physician, Ph.D. Student. Department of Endocrinology M / Steno Diabetes Centre Odense (SDCO). Odense University Hospital. J.B. Winsløvvej 4. DK- 5000 Odense, Denmark.

Nicolai Gundtoft Uhrenholt, Physician, Ph.D. Student. Psykiatrien Region Sjælland**,** Psykiatrien Vest, Fælledvej 6, Bygning 3, 4. sal, DK- 4200 Slagelse, Denmark.

b. <u>Competing interests:</u>

<u>The authors declare that there is no conflict of interest that would prejudice the impartiality of this scientific work.</u>

c. <u>Funding:</u>

Novo Nordisk Foundation: 8,584,959DKK (salary and tuition fee PHD Students and study-nurses, running expenses, biochemical and laboratory procedures, TAP personal, mileage allowance)

Research Grant from Steno Diabetes Center Zealand, Denmark: 660,000 DKK.

Research Grant from Steno Diabetes Center Odense, Denmark: 578,000 DKK.

Annum (Region Zealand) to PhD-student Nicolai Uhrenholt: 20.000 DDK per year for three years (60,000 DKK in total).

For peer review only - http://bmjopen.bmj.com/site/about/guidelines.xhtml

For peer review only

460 Slagelse Puljen, 2020: The Region of Zealand, Denmark: 240,000 DKK

461 Slagelse Puljen 2022: 150,000 DKK

462 Region Sjællands Sundhedsvidenskabelige Forskningsfond 2022: 252,631 DKK.

463 Furthermore, Novo Nordisk A/S will provide investigational drug and placebo free of charge for sponsor,
464 but is otherwise not involved in the study.

465

466

467

For peer review only

REFERENCES

1 D. S, M. H, C.U. C, *et al.* The prevalence of food addiction as assessed by the yale food addiction scale: A systematic review. *Eur J Nucl Med Mol Imaging*. 2017;74:719–28.

2 Lawrence D, Hancock KJ, Kisely S. The gap in life expectancy from preventable physical illness in psychiatric patients in Western Australia: Retrospective analysis of population based registers. *BMJ*. Published Online First: 2013. doi: 10.1136/bmj.f2539

3 Laursen TM, Plana-Ripoll O, Andersen PK, *et al.* Cause-specific life years lost among persons diagnosed with schizophrenia: Is it getting better or worse? *Schizophr Res*. 2019;206:284–90.

4 Correll CU, Solmi M, Croatto G, *et al.* World Psychiatry - 2022 - Correll - Mortality in people with schizophrenia a systematic review and meta-analysis of.pdf. 2022;249–71.

5 Nordentoft M, Wahlbeck K, Hällgren J, *et al.* Excess mortality, causes of death and life expectancy in 270,770 patients with recent onset of mental disorders in Denmark, Finland and Sweden. *PLoS One*. 2013;8:e55176.

6 Westman J, Eriksson S V., Gissler M, *et al.* Increased cardiovascular mortality in people with schizophrenia: A 24-year national register study. *Epidemiol Psychiatr Sci*. Published Online First: 2018. doi: 10.1017/S2045796017000166

7 De Hert M, Dekker JM, Wood D, *et al.* Cardiovascular disease and diabetes in people with severe mental illness position statement from the European Psychiatric Association (EPA), supported by the European Association for the Study of Diabetes (EASD) and the European Society of Cardiology (ESC. Eur. Psychiatry. 2009. https://doi.org/10.1016/j.eurpsy.2009.01.005

8 De Hert M, Correll CU, Bobes J, *et al.* Physical illness in patients with severe mental disorders. I. Prevalence, impact of medications and disparities in health care. *World Psychiatry*. 2011;10:52–77.

9 Vancampfort D, Stubbs B, Mitchell AJ, *et al.* Risk of metabolic syndrome and its components in

For peer review only - http://bmjopen.bmj.com/site/about/guidelines.xhtml

people with schizophrenia and related psychotic disorders, bipolar disorder and major depressive disorder: A systematic review and meta-analysis. *World Psychiatry*. 2015;14:339–47.

10 Leucht S, Cipriani A, Spineli L, *et al.* Comparative efficacy and tolerability of 15 antipsychotic drugs in schizophrenia: a multiple-treatments meta-analysis. *Lancet (London, England)*. 2013;382:951–62.

11 Tiihonen J, Wahlbeck K, Lönnqvist J, *et al.* Effectiveness of antipsychotic treatments in a nationwide cohort of patients in community care after first hospitalisation due to schizophrenia and schizoaffective disorder: Observational follow-up study. *Br Med J*. Published Online First: 2006. doi: 10.1136/bmj.38881.382755.2F

12 Maayan L, Correll CU. Weight gain and metabolic risks associated with antipsychotic medications in children and adolescents. *J Child Adolesc Psychopharmacol*. 2011;21:517–35.

13 Pillinger T, McCutcheon RA, Vano L, *et al.* Comparative effects of 18 antipsychotics on metabolic function in patients with schizophrenia, predictors of metabolic dysregulation, and association with psychopathology: a systematic review and network meta-analysis. *The lancet Psychiatry*. 2020;7:64–77.

14 Henderson DC, Vincenzi B, Andrea N V., *et al.* Pathophysiological mechanisms of increased cardiometabolic risk in people with schizophrenia and other severe mental illnesses. *The Lancet Psychiatry*. 2015;2:452–64.

15 DE Hert M, Correll CU, Bobes J, *et al.* Physical illness in patients with severe mental disorders. I. Prevalence, impact of medications and disparities in health care. *World Psychiatry*. 2011;10:52–77.

16 Allison DB, Mackell JA, McDonnell DD. The impact of weight gain on quality of life among persons with schizophrenia. *Psychiatr Serv*. 2003;54:565–7.

17 Strassnig M, Brar JS, Ganguli R. Body mass index and quality of life in community-dwelling patients with schizophrenia. *Schizophr Res*. 2003;62:73–6.

For peer review only

For peer review only - http://bmjopen.bmj.com/site/about/guidelines.xhtml

18 Faulkner G, Cohn T, Remington G, *et al.* Body mass index, waist circumference and quality of life in individuals with schizophrenia. *Schizophr Res*. 2007;90:174–8.

19 R.L. K, P.K. C-L, R.D. C, *et al.* Impact of obesity on health-related quality of life in schizophrenia and bipolar disorder. Obesity. 2008;16:749–54. http://ovidsp.ovid.com/ovidweb.cgi?T=JS&PAGE=reference&D=emed8&NEWS=N&AN=18239573

20 Sugawara N, Yasui-Furukori N, Sato Y, *et al.* Body mass index and quality of life among outpatients with schizophrenia in Japan. *BMC Psychiatry*. 2013;13. doi: 10.1186/1471-244X-13-108

21 Fond G, Korchia T, Sunhary de Verville PL, *et al.* Major depression, sleep, hostility and body mass index are associated with impaired quality of life in schizophrenia. Results from the FACE-SZ cohort. *J Affect Disord*. 2020;274:617–23.

22 Lieberman JA, Stroup TS, McEvoy JP, *et al.* Effectiveness of antipsychotic drugs in patients with chronic schizophrenia. *N Engl J Med*. 2005;353:1209–23.

23 Weiden PJ, Mackell JA, McDonnell DD. Obesity as a risk factor for antipsychotic noncompliance. *Schizophr Res*. 2004;66:51–7.

24 Cooper SJ, Reynolds GP, Barnes TRE, *et al.* BAP guidelines on the management of weight gain, metabolic disturbances and cardiovascular risk associated with psychosis and antipsychotic drug treatment. *J Psychopharmacol*. Published Online First: 2016. doi: 10.1177/0269881116645254

25 Holt RIG, Gossage-Worrall R, Hind D, *et al.* Structured lifestyle education for people with schizophrenia, schizoaffective disorder and first-episode psychosis (STEPWISE): Randomised controlled trial. *Br J Psychiatry*. 2019;214:63–73.

26 Zheng W, Li X-B, Tang Y-L, *et al.* Metformin for Weight Gain and Metabolic Abnormalities Associated With Antipsychotic Treatment: Meta-Analysis of Randomized Placebo-Controlled Trials. *J Clin Psychopharmacol*. 2015;35:499–509.

For peer review only

27 Larsen JR, Vedtofte L, Jakobsen MSL, *et al.* Effect of liraglutide treatment on prediabetes and overweight or obesity in clozapine- or olanzapine-treated patients with schizophrenia spectrum disorder: A randomized clinical trial. *JAMA Psychiatry*. Published Online First: 2017. doi: 10.1001/jamapsychiatry.2017.1220

28 Siskind D, Russell A, Gamble C, *et al.* RCT of exenatide for clozapine-associated obesity. *Schizophr Bull*. 2017.

29 Kouidrat Y, Amad A. GLP-1 agonists for metabolic disorders in schizophrenia. Schizophr. Res. 2019. https://doi.org/10.1016/j.schres.2018.09.019

30 D. S, M. H, C.U. C, *et al.* Glucagon-like peptide-1 receptor agonists for antipsychotic-associated cardio-metabolic risk factors: A systematic review and individual participant data meta-analysis. *Diabetes, Obes Metab*. 2019.

31 Rubino D, Abrahamsson N, Davies M, *et al.* Effect of Continued Weekly Subcutaneous Semaglutide vs Placebo on Weight Loss Maintenance in Adults with Overweight or Obesity: The STEP 4 Randomized Clinical Trial. *JAMA - J Am Med Assoc*. 2021;325:1414–25.

32 Fernández SG. Adherence to antipsychotic medication in bipolar disorder and schizophrenia patients: a systematic review. *Eur Neuropsychopharmacol*. Published Online First: 2016. doi: 10.1016/s0924-977x(16)31412-2

33 Muzurović E, Yumuk VD, Rizzo M. GLP-1 and dual GIP/GLP-1 receptor agonists in overweight/obese patients for atherosclerotic cardiovascular disease prevention: Where are we now? *J Diabetes Complications*. 2023;37:2023–5.

34 Lincoff AM, Brown-Frandsen K, Colhoun HM, *et al.* Semaglutide and Cardiovascular Outcomes in Obesity without Diabetes. *N Engl J Med*. 2023;389:2221–32.

35 El Abdellati K, De Picker L, Morrens M. Antipsychotic Treatment Failure: A Systematic Review on Risk Factors and Interventions for Treatment Adherence in Psychosis. *Front Neurosci*. 2020;14. doi:

For peer review only

10.3389/fnins.2020.531763

36 Kay SR, Fiszbein A, Opler LA. The positive and negative syndrome scale (PANSS) for schizophrenia. *Schizophr Bull*. Published Online First: 1987. doi: 10.1093/schbul/13.2.261

37 Østergaard SD, Foldager L, Mors O, *et al.* The Validity and Sensitivity of PANSS-6 in the Clinical Antipsychotic Trials of Intervention Effectiveness (CATIE) Study. *Schizophr Bull*. 2018;44:453–62.

38 Kølbæk P, Blicher AB, Buus CW, *et al.* Inter-rater reliability of ratings on the six-item Positive and Negative Syndrome Scale (PANSS-6) obtained using the Simplified Negative and Positive Symptoms Interview (SNAPSI). *Nord J Psychiatry*. 2018;72:431–6.

39 Ware JEJ, Sherbourne CD. The MOS 36-item short-form health survey (SF-36). I. Conceptual framework and item selection. *Med Care*. 1992;30:473–83.

40 Serhiyenko VA, Serhiyenko AA. Cardiac autonomic neuropathy: Risk factors, diagnosis and treatment. *World J Diabetes*. Published Online First: 2018. doi: 10.4239/wjd.v9.i1.1

41 Herder C, Roden M, Ziegler D. Novel Insights into Sensorimotor and Cardiovascular Autonomic Neuropathy from Recent-Onset Diabetes and Population-Based Cohorts. Trends Endocrinol. Metab. 2019. https://doi.org/10.1016/j.tem.2019.02.007

42 Poirier P, Hernandez TL, Weil KM, *et al.* Impact of diet-induced weight loss on the cardiac autonomic nervous system in severe obesity. *Obes Res*. Published Online First: 2003. doi: 10.1038/oby.2003.143

43 Moghbel M, Al-Zaghal A, Werner TJ, *et al.* The Role of PET in Evaluating Atherosclerosis: A Critical Review. Semin. Nucl. Med. 2018. https://doi.org/10.1053/j.semnuclmed.2018.07.001

44 McKenney-Drake ML, Moghbel MC, Paydary K, *et al.* 18F-NaF and 18F-FDG as molecular probes in the evaluation of atherosclerosis. Eur. J. Nucl. Med. Mol. Imaging. 2018. https://doi.org/10.1007/s00259-018-4078-0

For peer review only

45 Høilund-Carlsen PF, Moghbel MC, Gerke O, *et al.* Evolving Role of PET in Detecting and Characterizing Atherosclerosis. PET Clin. 2019. https://doi.org/10.1016/j.cpet.2018.12.001

46 Kolotkin RL, Crosby RD, Corey-Lisle PK, *et al.* Performance of a weight-related measure of Quality of Life in a psychiatric sample. *Qual life Res an Int J Qual life Asp Treat care Rehabil*. 2006;15:587–96.

47 Thompson K, Kulkarni J, Sergejew AA. Reliability and validity of a new Medication Adherence Rating Scale (MARS) for the psychoses. *Schizophr Res*. Published Online First: 2000. doi: 10.1016/S0920-9964(99)00130-9

48 Fialko L, Garety PA, Kuipers E, *et al.* A large-scale validation study of the Medication Adherence Rating Scale (MARS). *Schizophr Res*. 2008;100:53–9.

49 Rosenbaum S, Morell R, Abdel-Baki A, *et al.* Assessing physical activity in people with mental illness: 23-country reliability and validity of the simple physical activity questionnaire (SIMPAQ). *BMC Psychiatry*. 2020;20:108.

50 Rabin R, Gudex C, Selai C, *et al.* From translation to version management: a history and review of methods for the cultural adaptation of the EuroQol five-dimensional questionnaire. *Value Heal J Int Soc Pharmacoeconomics Outcomes Res*. 2014;17:70–6.

51 Sorli C, Harashima S ichi, Tsoukas GM, *et al.* Efficacy and safety of once-weekly semaglutide monotherapy versus placebo in patients with type 2 diabetes (SUSTAIN 1): a double-blind, randomised, placebo-controlled, parallel-group, multinational, multicentre phase 3a trial. *Lancet Diabetes Endocrinol*. Published Online First: 2017. doi: 10.1016/S2213-8587(17)30013-X

52 Larsen JR, Vedtofte L, Jakobsen MSL, *et al.* Effect of liraglutide treatment on prediabetes and overweight or obesity in clozapine- or olanzapine-treated patients with schizophrenia spectrum disorder: A randomized clinical trial. *JAMA Psychiatry*. 2017;74:719–28.

53 Speyer H, Christian Brix Nørgaard H, Birk M, *et al.* The CHANGE trial: No superiority of lifestyle coaching plus care coordination plus treatment as usual compared to treatment as usual alone in

For peer review only

For peer review only - http://bmjopen.bmj.com/site/about/guidelines.xhtml

reducing risk of cardiovascular disease in adults with schizophrenia spectrum disorders and abdominal obesity. *World Psychiatry*. 2016;15:155–65.

54 Verma S, Al-Omran M, Leiter LA, *et al.* Cardiovascular efficacy of liraglutide and semaglutide in individuals with diabetes and peripheral artery disease. *Diabetes, Obes Metab*. 2022;24:1288–99.

55 Marso SP, Bain SC, Consoli A, *et al.* Semaglutide and cardiovascular outcomes in patients with type 2 diabetes. *N Engl J Med*. Published Online First: 2016. doi: 10.1056/NEJMoa1607141

**Table 1**
**Inclusion criteria**
• Diagnosed with schizophrenia spectrum disorder (ICD10 codes DF20, DF21 or DF25)
• Age between 18 and 60 years (both included)
• Approved contraception for female participants
• Treated by one of the community mental health centers and general practitioners in the Region of Southern Denmark or Zealand
• Antipsychotic SGA treatment for at least 6 months
• Stable co-medication for at least 1 month
• HbA1c between 39-47 mmol/mol (both included). Two measurements with ≥3 month interval are required to confirm prediabetes. The first measurement is identified and obtained from patient journals, the second prior to enrolment

For peer review only

For peer review only - http://bmjopen.bmj.com/site/about/guidelines.xhtml

• BMI ≥27 kg/m^2. Two weights with ≥3 month interval are required to confirm obesity
• Capable of providing informed oral and written consent
**Exclusion criteria**
• Diagnosis of diabetes (T1D or T2D) or a HbA1c >47 mmol/mol
• Active malignant disease within the last 5 years
• Pregnancy or breast feeding
• A reported high consumption of alcohol or severe substance abuse
• Unwillingness to allow home visits by a study nurse
• Significant somatic disease: 1) end-stage renal failure (eGFR <15 ml/min); 2) elevated liver function tests (liver transaminases >2 times upper normal limit); 3) history of acute or chronic pancreatitis; 4) heart failure (NYHA class IV) or unstable angina pectoris or myocardial infarction with the last 6 months; 5) uncontrolled hypertension (systolic blood pressure >180 mm Hg, diastolic blood pressure >100 mm Hg)
• Previous treatment with study drug or use of other weight reducing drugs within last 6 month
• Participation in other drug trials
• Treatment with drugs approved for overt diabetes type 2 except metformin.
• Circumstances that the investigator believes will interfere with the trial

For peer review only - http://bmjopen.bmj.com/site/about/guidelines.xhtml

615

For peer review only - http://bmjopen.bmj.com/site/about/guidelines.xhtml

Figure Legend:

Figure 1 – Flow chart

For peer review only

For peer review only - http://bmjopen.bmj.com/site/about/guidelines.xhtml

For peer review only

For peer review only - http://bmjopen.bmj.com/site/about/guidelines.xhtml

CONSORT Flow Diagram

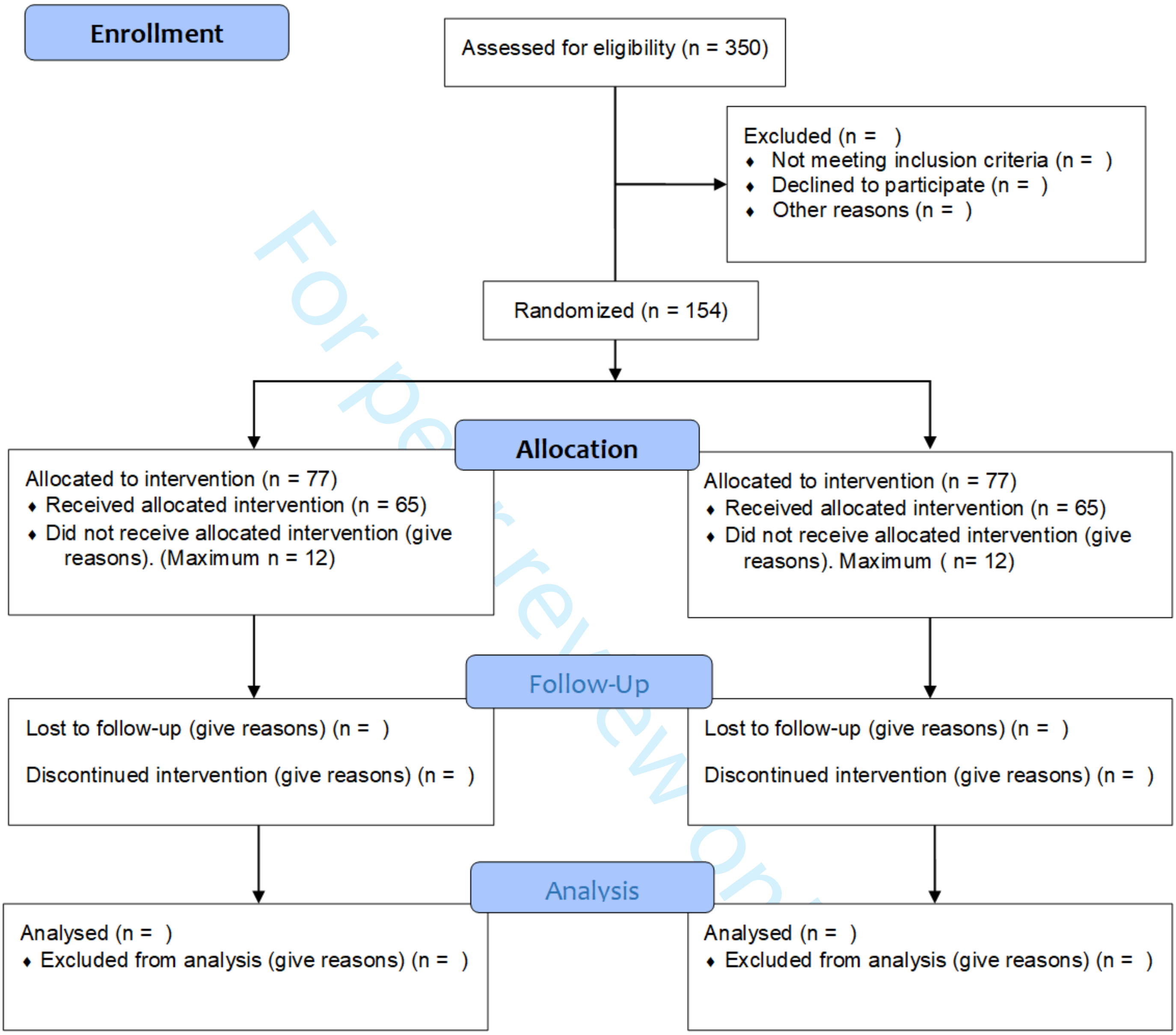

For peer review only - http://bmjopen.bmj.com/site/about/guidelines.xhtml

Supplementary Table 1

HISTORI	Investigator inclusion	Patient acceptance to participate	Week	Week	Week	Week	Week	Week	Week	Week	Week
			1	5	9	15	19	23	28	30	36
	Physical appearance of patients at hospitals	X				X				X	
			Weekly injection of Semaglutide / placebo								
Consent approval	X										
Baseline number		X									
Evaluation of medical history	X	X									
In- and Exclusion criteria	X	X									
Randomization		X									
Physical examination		X									
Blood pressure	X		X			X				X	
Weight/BMI	X		X			X				X	
Full biochemical screening		X								X	
Safety blood test	X					X					
PET CT scan		Scheduled							Scheduled		
PANNS-6, SF-36v2, IWQOL-Lite		X				X				X	
MARS, SIMPAQ, and EQ-5D		X	X	X	X	X	X	X	X	X	
Cardiac autonomic neuropathy (VAGUS®)		X				X				X	

**Adjustment of Semaglutide/placebo doses**				X	X						
**Semaglutide 0.25 mg**			X								
**Semaglutide 0.50 mg**				X							
**Semaglutide 1.00 mg per week**					X	X	X	X	X	X	
**End of study evaluation**											X

For peer review only

Reporting checklist for protocol of a clinical trial.

Based on the SPIRIT guidelines.

Instructions to authors

Complete this checklist by entering the page numbers from your manuscript where readers will find each of the items listed below.

Your article may not currently address all the items on the checklist. Please modify your text to include the missing information. If you are certain that an item does not apply, please write "n/a" and provide a short explanation.

Upload your completed checklist as an extra file when you submit to a journal.

In your methods section, say that you used the SPIRITreporting guidelines, and cite them as:

Chan A-W, Tetzlaff JM, Gøtzsche PC, Altman DG, Mann H, Berlin J, Dickersin K, Hróbjartsson A, Schulz KF, Parulekar WR, Krleža-Jerić K, Laupacis A, Moher D. SPIRIT 2013 Explanation and Elaboration: Guidance for protocols of clinical trials. BMJ. 2013;346:e7586

		Reporting Item	Page Number
**Administrative information**			
Title	#1	Descriptive title identifying the study design, population, interventions, and, if applicable, trial acronym	1

For peer review only

Trial registration	#2a	Trial identifier and registry name. If not yet registered, name of intended registry	3
Trial registration: data set	#2b	All items from the World Health Organization Trial Registration Data Set	3
Protocol version	#3	Date and version identifier	3
Funding	#4	Sources and types of financial, material, and other support	17-18
Roles and responsibilities: contributorship	#5a	Names, affiliations, and roles of protocol contributors	18
Roles and responsibilities: sponsor contact information	#5b	Name and contact information for the trial sponsor	18
Roles and responsibilities: sponsor and funder	#5c	Role of study sponsor and funders, if any, in study design; collection, management, analysis, and interpretation of data; writing of the report; and the decision to submit the report for publication, including whether they will have ultimate authority over any of these activities	18
Roles and responsibilities: committees	#5d	Composition, roles, and responsibilities of the coordinating centre, steering committee, endpoint adjudication committee, data management team, and	18-20

For peer review only

		other individuals or groups overseeing the trial, if applicable (see Item 21a for data monitoring committee)	
**Introduction**			
Background and rationale	#6a	Description of research question and justification for undertaking the trial, including summary of relevant studies (published and unpublished) examining benefits and harms for each intervention	4
Background and rationale: choice of comparators	#6b	Explanation for choice of comparators	4-6
Objectives	#7	Specific objectives or hypotheses	6
Trial design	#8	Description of trial design including type of trial (eg, parallel group, crossover, factorial, single group), allocation ratio, and framework (eg, superiority, equivalence, non-inferiority, exploratory)	7
**Methods: Participants, interventions, and outcomes**			
Study setting	#9	Description of study settings (eg, community clinic, academic hospital) and list of countries where data will be collected. Reference to where list of study sites can be obtained	7

For peer review only

Eligibility criteria	#10	Inclusion and exclusion criteria for participants. If applicable, eligibility criteria for study centres and individuals who will perform the interventions (eg, surgeons, psychotherapists)	7
Interventions: description	#11a	Interventions for each group with sufficient detail to allow replication, including how and when they will be administered	7-9
Interventions: modifications	#11b	Criteria for discontinuing or modifying allocated interventions for a given trial participant (eg, drug dose change in response to harms, participant request, or improving / worsening disease)	11-12
Interventions: adherance	#11c	Strategies to improve adherence to intervention protocols, and any procedures for monitoring adherence (eg, drug tablet return; laboratory tests)	8
Interventions: concomitant care	#11d	Relevant concomitant care and interventions that are permitted or prohibited during the trial	9
Outcomes	#12	Primary, secondary, and other outcomes, including the specific measurement variable (eg, systolic blood pressure), analysis metric (eg, change from baseline, final value, time to event), method of aggregation (eg, median, proportion), and time point for each outcome. Explanation of the clinical relevance of chosen efficacy and harm outcomes is strongly recommended	9-11

For peer review only

Participant timeline	#13	Time schedule of enrolment, interventions (including any run-ins and washouts), assessments, and visits for participants. A schematic diagram is highly recommended (see Figure)	Table 1.2
Sample size	#14	Estimated number of participants needed to achieve study objectives and how it was determined, including clinical and statistical assumptions supporting any sample size calculations	12
Recruitment	#15	Strategies for achieving adequate participant enrolment to reach target sample size	7
**Methods: Assignment of interventions (for controlled trials)**			
Allocation: sequence generation	#16a	Method of generating the allocation sequence (eg, computer-generated random numbers), and list of any factors for stratification. To reduce predictability of a random sequence, details of any planned restriction (eg, blocking) should be provided in a separate document that is unavailable to those who enrol participants or assign interventions	7
Allocation concealment mechanism	#16b	Mechanism of implementing the allocation sequence (eg, central telephone; sequentially numbered, opaque,	8

For peer review only

		sealed envelopes), describing any steps to conceal the sequence until interventions are assigned	
Allocation: implementation	#16c	Who will generate the allocation sequence, who will enrol participants, and who will assign participants to interventions	8
Blinding (masking)	#17a	Who will be blinded after assignment to interventions (eg, trial participants, care providers, outcome assessors, data analysts), and how	7
Blinding (masking): emergency unblinding	#17b	If blinded, circumstances under which unblinding is permissible, and procedure for revealing a participant’s allocated intervention during the trial	12
**Methods: Data collection, management, and analysis**			
Data collection plan	#18a	Plans for assessment and collection of outcome, baseline, and other trial data, including any related processes to promote data quality (eg, duplicate measurements, training of assessors) and a description of study instruments (eg, questionnaires, laboratory tests) along with their reliability and validity, if known. Reference to where data collection forms can be found, if not in the protocol	9

For peer review only

Data collection plan: retention	#18b	Plans to promote participant retention and complete follow-up, including list of any outcome data to be collected for participants who discontinue or deviate from intervention protocols	table 1.2
Data management	#19	Plans for data entry, coding, security, and storage, including any related processes to promote data quality (eg, double data entry; range checks for data values). Reference to where details of data management procedures can be found, if not in the protocol	7
Statistics: outcomes	#20a	Statistical methods for analysing primary and secondary outcomes. Reference to where other details of the statistical analysis plan can be found, if not in the protocol	12
Statistics: additional analyses	#20b	Methods for any additional analyses (eg, subgroup and adjusted analyses)	12
Statistics: analysis population and missing data	#20c	Definition of analysis population relating to protocol non-adherence (eg, as randomised analysis), and any statistical methods to handle missing data (eg, multiple imputation)	12-14

Methods: Monitoring

Data monitoring: formal committee	#21a	Composition of data monitoring committee (DMC); summary of its role and reporting structure; statement of whether it is independent from the sponsor and competing interests; and reference to where further	14

For peer review only

		details about its charter can be found, if not in the protocol. Alternatively, an explanation of why a DMC is not needed	
Data monitoring: interim analysis	#21b	Description of any interim analyses and stopping guidelines, including who will have access to these interim results and make the final decision to terminate the trial	13-14
Harms	#22	Plans for collecting, assessing, reporting, and managing solicited and spontaneously reported adverse events and other unintended effects of trial interventions or trial conduct	11
Auditing	#23	Frequency and procedures for auditing trial conduct, if any, and whether the process will be independent from investigators and the sponsor	11
**Ethics and dissemination**			
Research ethics approval	#24	Plans for seeking research ethics committee / institutional review board (REC / IRB) approval	14
Protocol amendments	#25	Plans for communicating important protocol modifications (eg, changes to eligibility criteria, outcomes, analyses) to relevant parties (eg, investigators, REC / IRBs, trial participants, trial registries, journals, regulators)	14

For peer review only

Consent or assent	#26a	Who will obtain informed consent or assent from potential trial participants or authorised surrogates, and how (see Item 32)	7
Consent or assent: ancillary studies	#26b	Additional consent provisions for collection and use of participant data and biological specimens in ancillary studies, if applicable	7
Confidentiality	#27	How personal information about potential and enrolled participants will be collected, shared, and maintained in order to protect confidentiality before, during, and after the trial	15
Declaration of interests	#28	Financial and other competing interests for principal investigators for the overall trial and each study site	17
Data access	#29	Statement of who will have access to the final trial dataset, and disclosure of contractual agreements that limit such access for investigators	19
Ancillary and post trial care	#30	Provisions, if any, for ancillary and post-trial care, and for compensation to those who suffer harm from trial participation	19
Dissemination policy: trial results	#31a	Plans for investigators and sponsor to communicate trial results to participants, healthcare professionals, the public, and other relevant groups (eg, via publication, reporting in results databases, or other data sharing arrangements), including any publication restrictions	18

For peer review only

Dissemination policy: authorship	#31b	Authorship eligibility guidelines and any intended use of professional writers	18
Dissemination policy: reproducible research	#31c	Plans, if any, for granting public access to the full protocol, participant-level dataset, and statistical code	15

Appendices

Informed consent materials	#32	Model consent form and other related documentation given to participants and authorised surrogates	1
Biological specimens	#33	Plans for collection, laboratory evaluation, and storage of biological specimens for genetic or molecular analysis in the current trial and for future use in ancillary studies, if applicable	n/a

The SPIRIT Explanation and Elaboration paper is distributed under the terms of the Creative Commons Attribution License CC-BY-NC. This checklist was completed on 07. June 2023 using https://www.goodreports.org/, a tool made by the EQUATOR Network in collaboration with Penelope.ai

For peer review only

BMJ Open

Home based Intervention with Semaglutide Treatment of Neuroleptic-Related Prediabetes (HISTORI): protocol describing a prospective, randomized, placebo controlled and double blinded multicenter trial

Journal:	*BMJ Open*
Manuscript ID	bmjopen-2023-077173.R2
Article Type:	Protocol
Date Submitted by the Author:	01-Mar-2024
Complete List of Authors:	Ganeshalingam, Ashok; Odense Universitetshospital, Endocrine Research Unit, Department of Endocrinology, Odense University Hospital & Department of Clinical Research, Faculty of Health, ; Lillebaelt Hospital - University Hospital of Southern Denmark, Department of Internal Medicine<br>Uhrenholt, Nicolai; Psykiatrien Region Sjælland Psykiatrien Vest<br>Arnfred, Sidse; University of Copenhagen,<br>Gæde, Peter; Slagelse Hospital, Department of Cardiology and Endocrinology<br>Bilenberg, Niels; University of Southern Denmark, Department of Clinical Research, Faculty of Health Sciences, University of Southern Denmark, Odense, Denmark.; University of Southern Denmark, Department of Child and Adolescent Mental Health Odense, Mental Health Services in the Region of Southern Denmark, University of Southern Denmark, Odense, Denmark.<br>Frystyk, Jan; Odense University Hospital Department of Endocrinology
**Primary Subject Heading**:	Mental health
Secondary Subject Heading:	Diabetes and endocrinology, Mental health, Cardiovascular medicine, Medical management
Keywords:	PREVENTIVE MEDICINE, General diabetes < DIABETES & ENDOCRINOLOGY, Schizophrenia & psychotic disorders < PSYCHIATRY, Primary Prevention, Obesity

SCHOLARONE™
Manuscripts

HISTORI

Home based Intervention with Semaglutide Treatment of Neuroleptic-Related Prediabetes (HISTORI): protocol describing a prospective, randomized, placebo controlled and double blinded multicenter trial

A. Ganeshalingam [1,2,3,6], N. Uhrenholt [4], S. Arnfred [4], P. Gæde [5], N. Bilenberg [3], J. Frystyk [1]

1. Endocrine Research Unit, Department of Endocrinology, Odense University Hospital & Department of Clinical Research, Faculty of Health, University of Southern Denmark, Odense, Denmark
2. Department of Internal Medicine, Lillebaelt Hospital, University Hospital of Southern Denmark, Kolding, Denmark
3. Mental Health Services in the Region of Southern Denmark, Child and Adolescent Psychiatry Odense, Odense C, Denmark
4. Psychiatry West, Region Zealand, Research Unit for Psychotherapy and Psychopathology, Slagelse, Denmark
5. Næstved, Slagelse og Ringsted Hospital, Department of cardiology and endocrinology, Slagelse, Denmark
6. Steno Diabetes Center Odense, Odense University Hospital, Denmark

For peer review only

Running title: Patients with prediabetes and schizophrenia on Semaglutide

Keywords:

Words in main text: 4772
Number of Tables: 2
Number of Figures: 1
Number of References: 55

Corresponding author

Dr. Ashok Ganeshalingam

For peer review only - http://bmjopen.bmj.com/site/about/guidelines.xhtml

Department of Endocrinology
Odense University Hospital
Kløverværnget 6, entrance 93
DK-5000 Odense C, Denmark.
E-mail: Ashok.Ganeshalingam@rsyd.dk

ABSTRACT

Introduction: Subjects with schizophrenia have a 2 to 3 fold higher mortality rate than the general population, and a reduced life expectancy of 10 to 20 years. Approximately 1/3 of this excess mortality has been attributed to obesity-related type 2 diabetes (T2D) and to cardiovascular disease (CVD). Glucagon-like peptide-1 (GLP-1) analogs increase satiety and delay gastric emptying, thereby reducing food intake and weight. GLP-1 analogs also exert beneficial effects on cardiovascular outcomes in high-risk T2D patients.

Our aim is to investigate whether 30 weeks add-on treatment with the GLP-1 analog Semaglutide can reduce HbA1c sufficiently to reverse prediabetes and the metabolic syndrome (MetS) in overweight schizophrenic patients.

Methods and analysis: We will perform a 30 week, two-armed, multi-center, superiority, double-blinded, randomized trial investigating the effect of weekly injections of Semaglutide vs placebo in mental health facilities in Region of Southern Denmark and Region of Zealand, Denmark. In total 154 adults with schizophrenia spectrum disease, aged 18 - 60 years treated with second generation anti-psychotic (SGA) treatment, HbA1c 39-47 mmol/mol and BMI > 27 kg/m^2 will be randomized to injections of 1.0 mg Semaglutide or placebo. The primary outcome is changes in HbA1c. Secondary outcomes encompass metabolic measures, psychotic symptoms and QOL. Exploratory outcomes encompass insulin sensitivity, cardiovascular risk profile, , medication adherence, general well-being and physical activity.

For peer review only

For peer review only - http://bmjopen.bmj.com/site/about/guidelines.xhtml

Ethics and dissemination: This study will be carried out in accordance with the Helsinki Declaration and Good Clinical Practice guidelines. This research has obtained approval from both the Danish Medicines Agency and The Regional Committees on Health Research Ethics for Southern Denmark.

Trial Registration number NCT05193578 European Clinical Trials Database Number (EudraCT) 2020-004374-22, Regional Ethical Committee number S-20200182

Based on protocol version 3.2, approved 19th April 2023.

Strengths and limitations of this study

- A 30-week trial designed with feedback from patients and relatives, conducted as a double-blinded, randomized, parallel-group, and placebo-controlled study, adhering to good clinical practice standards and monitored across multiple centers.

- The integration of endocrinology and psychiatry in our study holds significant value as it provides a holistic understanding of the intricate interplay between hormonal regulation and mental health, offering potential insights into innovative approaches for comprehensive patient care.

- This study employs a unique home-based intervention approach, facilitating data collection within participants' natural environments, enabling individuals experiencing severe symptoms of schizophrenia to engage in the study.

- Despite the significance of the study, it is important to note that the 30-week trial duration may impose limitations on fully exploring the comprehensive potential of GLP-1 receptor agonists.

For peer review only - http://bmjopen.bmj.com/site/about/guidelines.xhtml

BACKGROUND AND SIGNIFICANCE OF HISTORI

The life expectancy of subjects with schizophrenia spectrum disorders (hereafter schizophrenia) is reduced by 10 to 20 years when compared to the general population[1][2][3][4]. The excess mortality is partly explained by a 2 to 3 fold increase in cardiovascular disease (CVD) [5] [6]. A nationwide register study from Sweden, including more than 46,000 subjects with schizophrenia showed that CVD was the leading cause of death, causing more deaths than suicide, and that death from CVD occurred 10 years earlier in schizophrenic subjects than in the general population [6].

Another factor predisposing to the reduced life expectancy is type 2 diabetes (T2D), which develops in 10 to 15 % of all patients with schizophrenia [7]. Compared to the general population, patients with schizophrenia are more likely to have diabetes, hypertension, dyslipidemia, to be overweight and to smoke [8]. Indeed, a third of patients with schizophrenia have been reported to suffer from the metabolic syndrome (MetS) [9].

Antipsychotic treatment is vital to reduce schizophrenia related symptoms [10] and treatment with second generation anti-psychotic (SGA) drugs has demonstrated beneficial effects on mortality, suicide rates and hospitalizations [11]. However, SGA treatment comes with unfortunate side effects, which typically include increased risks of developing obesity, MetS and prediabetes [12] [13]. Furthermore, SGA treatment *per se* leads to impaired glucose tolerance, diabetes, hyperlipidemia and CVD [14] [15].

Gaining weight is associated with a reduced quality of life (QoL) in people with schizophrenia [16–21]. In addition, weight gain and MetS are among the most frequently reported reasons for discontinuation of treatment in this group[22]. Indeed, in a cohort of 304 subjects with schizophrenia, 94% reported distress over body weight, and those who were obese appeared to be more than twice as likely to be non-adherent compared to those with a BMI below 25 kg/m^2 [23]. Therefore, it may be speculated that weight-reducing treatments may lead to an increased adherence to SGA treatment. However, to the best of our knowledge

For peer review only - http://bmjopen.bmj.com/site/about/guidelines.xhtml

the association between a Glucagon-like Peptide 1 (GLP-1) agonist-induced weight reduction, the severity of psychotic symptoms, and the ability to adhere to SGA treatment has never been investigated in a randomized clinical trial (RCT).

Lifestyle and environmental factors such as smoking, poor diet and an insufficient capacity to exercise contribute to the risk of CVD [14]. Behavioral lifestyle interventions aiming at improving diet and physical activity have indeed shown short-term beneficial effects, but long term effects diminish over time compared to standard care[24] [25]. When lifestyle programs fail to reduce the increased risk of CVD and T2D, metformin is standard treatment according to NICE guidelines (NICE 2012). In a meta-analysis of 19 RCTs (N=1,279) the addition of metformin to ongoing anti-psychotic treatment for an average of 3 to 4 months in subjects with severe mental illness reduced body weight but only by 0.61 kg [26]. Therefore, alternative weight reducing drugs are required, e.g. GLP-1 analogs.

The potential of GLP-1 analogs to improve the metabolic profile of patients with schizophrenia was recently successfully tested. In a randomized controlled trial, including 97 patients (placebo/active comparator: 50/47) with schizophrenia, prediabetes and a BMI ≥27 kg/m^2, 16 weeks of Liraglutide (1.8 mg Victoza® daily) reduced body weight, waist circumference and abdominal fat mass, LDL cholesterol, glucose levels and insulin resistance, without any worsening in the psychiatric disorder [27]. The beneficial effect of GLP-1 analog treatment in SGA-treated patients was later re-confirmed in a 2018 meta-analysis which, however, only included two additional and smaller trials of Exanatide (Bydureon®), and only one of these studies was placebo-controlled [28]. Nevertheless, the review concluded that GLP-1 analog treatment is effective and tolerable for SGA-related weight gain, but that more studies are required[29] [30].

Today, liraglutide has been replaced by semaglutide, which is more efficient as a weight losing agent in obese subjects [31]. Furthermore, semaglutide is easier to administer than liraglutide as semaglutide requires weekly and not daily injections. This may be important as it opens for the possibility of administering

For peer review only - http://bmjopen.bmj.com/site/about/guidelines.xhtml

125 semaglutide by mobile nurses performing weekly house calls, thereby improving adherence to treatment, 126 which is known to be reduced in patients with schizophrenia [32]. Importantly, in non-diabetic obese 127 subjects, semaglutide is able to improve various cardio metabolic risk factors [33] and to prevent new 128 cardiovascular events (death from cardiovascular causes as well as nonfatal myocardial infarction and stroke) 129 in subjects with preexisting cardiovascular disease [34]. Therefore, semaglutide appears as a suitable 130 treatment for obese schizophrenic patients.

131 Aim and hypothesis

132 The aim of this study is to perform a randomized placebo-controlled double-blinded trial of Semaglutide 1.0 133 mg in comparison to placebo in patients with schizophrenia spectrum disorders (SZD), i.e., either 134 schizophrenia, schizotypal disorder or schizoaffective disorder, and comorbid obesity and prediabetes. (The 135 study was planned and initiated prior to the release of Semaglutide 2.4 mg; Wegovy®).

136 We hypothesize that administration of Semaglutide at a dose of 1.0 mg administered subcutaneously once a 137 week for 30 weeks improves HbA1c (primary outcome) in SGA-treated adults with SZD suffering from 138 prediabetes (HbA1c between 37 and 47 mmol/mol) and a BMI ≥27 kg/m^2, when compared to placebo. 139 Additionally, that Semaglutide improves the following secondary endpoints: weight, schizophrenic symptoms 140 (PANSS-6), QoL-related measures, patient-related outcomes (PRO data) and the cardiometabolic risk factor 141 profile.

142 METHOD

143 Design. The current trial is an investigator-initiated, double-blinded, parallel, superiority, multi-center RCT 144 with a 1:1 randomization. In total, we will include 154 patients recruited from two Danish regional mental 145 health services (MHS). A CONSORT diagram is provided in **Figure 1**. Data management is purely digital and

For peer review only - http://bmjopen.bmj.com/site/about/guidelines.xhtml

managed by Open Patient data Explorative Network (OPEN), Odense University Hospital, Denmark, using REDCAP ©.

SETTING

The study will recruit patients from community mental health centers and private practitioners in the Region of Southern Denmark and the Region of Zealand, Denmark.

PARTICIPANTS

Patients with a clinical diagnosis of either schizophrenia, schizotypal disorder or schizoaffective disorder (ICD-10 diagnoses: F20.x; F21; F25.x), age between 18 and 60 years receiving treatment in the regional mental health care of participating centers, or by private practice, and fulfilling criteria as listed in **Table 1**.

PROCEDURES

Recruitment

Patients fulfilling inclusion criteria will be invited from community mental health center clinics and private practices in the Region of Southern Denmark and the Region of Zealand. According to Danish clinical guidelines, patients treated with neuroleptics (almost exclusively SGAs) require minimum one annual laboratory test and clinical evaluation to test for the presence of the MetS. Eligible subjects accepting to participate will be invited to screening visits and allocated to treatment with placebo or Semaglutide at a dose of 1.0 mg administered subcutaneously once a week by blinded randomization, if all inclusion criteria and none of the exclusion criteria are met. The PhD students will be in charge of recruitment participants, obtaining consent to participation, initiation of medication and execution of the trial including inclusion visits and follow-up visits.

For peer review only - http://bmjopen.bmj.com/site/about/guidelines.xhtml

167 ## Randomization and concealment

168 A total of 154 participants will be enrolled and randomized 1:1 to Semaglutide or placebo. Block
169 randomization including blocks of either 4 or 6 randomization numbers will be used, and the randomizations
170 list will be provided by the manufacturer of Semaglutide and placebo (Novo Nordisk A/S, Novo Allé, Bagsværd
171 DK-2880 Denmark). Randomization will be performed using REDCAP ©.

172 ### *Mobile project nurses*

173 The presence of schizophrenia impacts adherence and accordingly, adherence to study medication may be
174 a challenge [35]. Therefore, we perform data collection at home by mobile study nurses to ensure optimal
175 adherence and to minimize discomfort for the participants.

176 The study nurse will visit the subjects in their homes to administer injection and teach eligible subjects to
177 self-inject under supervision. **Thus, apart from the enrolment, we plan for a home-based study.** If some
178 patients are able to adhere to treatment and to self-inject, weekly visits may not be necessary. Thus, the
179 frequency of home visits may be downscaled to involve monthly collection of data only. All data collection
180 is allocated to study nurses and the PhD-students, and the participants will maintain management-as-usual
181 (MAU) in the respective clinics from where they are recruited.

182 ## Interventions

183 The intervention consists of 30 weeks of treatment with once-weekly subcutaneous injections of either
184 Semaglutide (1.34 mg/ml; 1.0 mg) or placebo. The current dose adjustment guidelines for Semaglutide will
185 be followed (i.e., starting at 0.25 mg weekly for 4 weeks, then 0.5 mg weekly for 4 weeks, and finally 1 mg
186 or highest tolerated weekly dose until the end of intervention) or placebo (corresponding volume).
187 Subjects, who develops side effects when reaching maximum dose, will be reduced to the highest possible
188 tolerated dose of Semaglutide or placebo for the rest of the study.

For peer review only - http://bmjopen.bmj.com/site/about/guidelines.xhtml

Relevant concomitant care and interventions are permitted during the trial.

Patient and Public Involvement

The project's design was developed in collaboration with individuals diagnosed with schizophrenia and their family members. Their valuable contributions have provided insights into the possibilities of conducting examinations within a home-based setting.

Ethics and dissemination

This research has obtained approval from both the Danish Medicines Agency and The Regional Committees on Health Research Ethics for Southern Denmark

OUTCOMES

Primary outcome

Absolute reduction in HbA1c from baseline to end of treatment (30 weeks).

Secondary outcomes

During the treatment and after 30 weeks of treatment:

a) **Metabolic measures**: BMI, blood pressure, triglyceride, cholesterol (HDL and LDL), waist circumference and HOMA-estimates based on fasting insulin and plasma glucose.
Blood test will be taken at the nearest hospital prior to inclusion, at the inclusion, at 15 weeks and at end of study. BMI, waist circumference and blood pressure will be measured at week 1, 15 and 30.

b) **Positive and Negative Symptom Scale 6 (PANSS-6):** PANSS is an operationalized rating of positive and negative symptoms, in people with schizophrenia, which is sensitive to change [36]. PANSS-6 is a shorter version of the original 30-item version. It is validated and translated into Danish and has

For peer review only

For peer review only - http://bmjopen.bmj.com/site/about/guidelines.xhtml

proven a good level of inter-rater reliability when rated based on the Simplified Negative and Positive Symptoms Interview (SNAPSI) [37,38] .PANSS-6 interview will be performed at baseline, and after 15 and 30 weeks.

c) **Short Form Survey (SF-36)** is a well-researched, self-reported measure of health. It comprises 36 questions in 8 different domains. The survey will be applied in Danish, using the latest updated version 2 (SF-36v2) [39]. SF-36v2 will be measured at baseline, and after 15 and 30 weeks.

Exploratory outcomes:

a) **Cardiovascular risk markers:** cardiovascular autonomic neuropathy (CAN) (Vagus TM®), and circulating levels of cardiovascular risk markers. CAN is considered a serious complication of diabetes, being associated with a 5-fold increased risk of CVD [40]. Of interest to this study, CAN may be present prior to the development of diabetes [41] and it can be improved by weight loss [42]. As CAN may be measured by a hand held apparatus (Vagus TM®), measurements are easily performed in the homes of the enrolled patients. CAN will be measured at inclusion, at 15 weeks and at end of study.

Moreover, the CAN results will be juxtaposed with various cardio-metabolic risk markers, including proteins belonging to the GDF-family, activin, follistatin, the insulin-like growth factor (IGF)-system and its regulating proteins and enzymes, and the adipokines leptin and adiponectin. All these data are explorative tertiary outcomes.

b) **Pro-atherosclerotic changes:** we want to perform a pilot study in a subsample of 20 participants from placebo vs. 20 participants from the Semaglutide group, focusing on pre-symptomatic atherosclerosis assessed by positron emission tomography/computed tomography (PET/CT) using 18F-sodium fluoride (NaF) as tracer. NaF-PET/CT is a novel way of detecting and measuring calcification in major arteries, potentially years or decades before manifest arterial wall calcification

For peer review only - http://bmjopen.bmj.com/site/about/guidelines.xhtml

becomes detectable by cardiac CT [43,44]. We plan to perform two NaFPET/CT acquisitions, i.e. at baseline and after 30 weeks treatment. Based on our experience, inclusion of 20 placebo- and 20 Semaglutide-treated subjects is suitable to perform the pilot study. We hypothesize a ≥10% decrease in global disease score [45] as measured by NaF uptake in the heart and/or thoracic aorta, will be present after 30 weeks treatment.

c) **Impact of Weight on Quality of Life-Lite (IWQOL-Lite):** IWQOL-Lite is a reliable and valid self-report measure for assessing weight-related QoL in people with schizophrenia [46]. It provides a total score plus scores on five domains (physical function, self-esteem, sexual life, public distress, and work). It is translated into 83 languages, including Danish. IWQOL-Lite will be measured at baseline, and after 15 and 30 weeks.

d) **Medication Adherence Rating Scale (MARS):** MARS is a validated and reliable measure of adherence for psychoactive medication. MARS constitutes 10 items rating the medical adherence [47] [48]. MARS will be measured at baseline, and at weeks 5, 9, 15, 19, 23, 28 and 30.

e) **The Simple Physical Activity Questionnaire (SIMPAQ):** SIMPAQ has shown good test-retest reliability and has been validated against measures from Actigraph accelerometers [49]. SIMPAQ will be measured at baseline, and at weeks 5, 9, 15, 19, 23, 28 and 30.

f) **The EuroQol five-dimensional (EQ-5D)** questionnaire is a widely applied instrument for the measurement and cost-benefit evaluation of health interventions [50]. EQ-5D will be measured at baseline, and at weeks 5, 9, 15, 19, 23, 28 and 30.

A schedule of follow-up activities is shown in supplementary table 1.

For peer review only

For peer review only - http://bmjopen.bmj.com/site/about/guidelines.xhtml

SAFETY ASSESSMENTS

Adverse reactions will be evaluated weekly. At every 4-week visit, vital parameters will be recorded, including blood pressure, body weight and waist circumference. The safety parameters in the study include adverse reactions, vital parameters and liver and kidney function, which will be analyzed at baseline, week 15 and week 30. If any side effects or changes in biochemical measures are observed, the trial physician or the physician responsible for the test will be informed.

All safety parameters will be recorded in an electronic case report form (eCRF) as soon as they have either been measured or reported by the study participant. The study will be monitored by good clinical practice (GCP) and follow current GCP guidelines. The study protocol has been reviewed by GCP prior to inclusion of first participant, and subsequently, the study is monitored by GCP throughout the study period.

After drug administration, all potential side effects and other adverse events (AEs) observed will be reported in the eCRF. Semaglutide half-life is one week and thus expected to be completely eliminated after six weeks). Consequently, collection of AEs for all patients will be completed six weeks after the final injection by telephone calls. The nature of each AE will be assessed according to the severity and relationship of the experimental medicine or trial procedure, and will be assessed by the primary investigator, who is a physician. If it is considered that the AE is related to the test drug or test procedure, it will be noted as a side effect in the eCRF. All severe adverse events (SAEs) will be reported to the sponsor. Hospital admissions related to schizophrenia as soon as possible, other hospital admissions always within 24 hours of it coming to our attention. All severe adverse reactions (SARs) which, regardless of dose, result in death, are life-threatening, result in hospitalization or prolongation of hospital stay in medical or surgical departments, result in significant or persistent disability or incapacity, or lead to congenital anomaly or malformation, will be considered in this trial to be unexpected (SUSAR).

For peer review only

For peer review only - http://bmjopen.bmj.com/site/about/guidelines.xhtml

276 Emergency un-blinding will occur if the participants develop adverse events that require knowledge of the
277 treatment, if patient develop sign of overdose or if patient develop conditions that require treatment with
278 the trial drug (Semaglutide) or drugs with potential interactions with trial drug.

279 STATISTICAL CONSIDERATIONS

280 Primary outcomes for the study will be absolute changes in HbA1c. Changes in HbA1c will be evaluated
281 after 15 weeks and after 30 weeks.

282 Assuming that 30 weeks of Semaglutide reduces HbA1c by 0.2%, and that SD equals 0.35%, **65 subjects in**
283 **each arm are required to obtain a power of 90%**, using a 2-sided significance level of 5%.

284 Dropout from trials concerning pharmacological treatment for obesity is non-linear; the dropout from the
285 treatment group will occur earlier due to side effects, while in the placebo group dropouts occur later due
286 to lack of efficacy.

287 Feasibility

288 In the SUSTAIN 1 trial (30 weeks of Semaglutide in patients with T2D), the discontinuation of study drug
289 amounted to 13% in those receiving 0.5 mg Semaglutide, 12% in those receiving 1.0 mg Semaglutide, and
290 11% in placebo-treated subjects [51]. In the 16-week study with Liraglutide in patients with schizophrenia,
291 23 of 214 patients declined to participate, and 111 patients did not meet inclusion criteria, leaving 103 to
292 be enrolled. Of these, only 7 patients dropped out of the study, leaving an overall success rate of 45
293 percent [52]. We believe to experience similar rates. Thus, we need to approach approximately 350
294 patients to enroll 154 patients. Given the number of potentially eligible patients in the two regions
295 (currently estimated to >300 excluding new cases in the age 18-60 years), we believe this is feasible. In the
296 present study, we will follow the participants for 30 weeks. Hence, we predict a higher risk of dropout.

For peer review only - http://bmjopen.bmj.com/site/about/guidelines.xhtml

Accordingly, we conservatively assume a dropout rate of 15 percent to ensure that the required number of subjects to obtain power are recruited.

In summary, our study was powered to examine a 0.2% decline in HbA1c, despite a drop-out rate of 15%, resulting in 77 patients in each arm. Initially, we considered implementation of an oral glucose tolerance test. However, after careful consideration, we concluded that such a test would not be feasible in a home-based setting. In seeking guidance, our user panel recommended against the oral glucose tolerance test, citing concerns about its practicality for patients. Taking this valuable input into account, we decided to refrain from incorporating the test, recognizing the potential challenges it may pose for patients in performing such a procedure.

Although this study is not designed as treatment for obesity, we believe the estimation of dropout is reliable as the study focus on the same patient population and the pharmacological profile of the investigated drug is similar to Liraglutide [52].

DATA ANALYSIS

All participants randomized in the study will be included in the assessment of endpoints. All data will be analyzed based on the intention-to-treat population. Clinical results will be presented as mean, least squares mean, least square mean change from baseline or least square difference between groups with SE, SD or two-sided 95% CI as appropriate. Statistical tests will be conducted as two-sided tests with a 5% significance level.

A mixed effects linear regression analysis will be used to compare changes in glucose tolerance, HbA1c, from baseline to 30 weeks follow-up between the intervention and the placebo groups. Missing values will be imputed, primary analysis will be intention-to-treat. Supplementary per protocol analysis will accompany the intention to treat analysis.

For peer review only

For peer review only - http://bmjopen.bmj.com/site/about/guidelines.xhtml

All changes in secondary outcomes from baseline to various follow-up times are analyzed using mixed effects linear regression analyses for continuous outcomes and mixed effects logistic regression analyses for categorical outcomes. All analyses on continuous outcomes are adjusted for the baseline value of the outcome, and in case of significant imbalances in baseline patient characteristics between the two randomized groups, exploratory analyses including such variables are conducted. To correct for multiple testing of the secondary outcomes, Holm's sequentially rejective multiple test procedure will be applied. Statistical analyses are performed using Stata software Version 16 (Statacorp, Texas, USA). The hypothesis tests are 2-sided, and the 5% level of significance is considered.

ETHICAL APPROVAL AND CONSENT TO PARTICIPATE

The project is approved by the Regional Science Ethical Committee for the Region of Southern Denmark and the Danish Medicine Agency. Data processing in connection with the study is reported to the region's list of on-going research projects via the Executive Secretariat at Odense University Hospital (OUH). The study participants will not necessarily be able to benefit from participation in the study. Individual participants may be diagnosed with illness that may require treatment; in that case, they will be referred for relevant treatment. Participants will not have overt diabetes based on HbA1c. Therefore, following national guidelines, participants in this study are not candidates for standard treatment of diabetes and under normal circumstances they would not receive antidiabetic treatment or treatment that would reduce their risk of diabetes. However, Metformin treatment of the MetS is accepted.

Amendments to the study protocol will be made to the Regional Science Ethical Committee for the Region of Southern Denmark and the Danish Medicine Agency.

The study will be conducted in accordance with the Helsinki Declaration with amendments as well as in accordance with the General Data Protection Regulation (GDPR), and the Danish Data Protection. In the case that biological material will be shipped to collaborators abroad, the Danish Protection Agency will be

For peer review only - http://bmjopen.bmj.com/site/about/guidelines.xhtml

applied and Chapter V of the Danish Protection Act will be obeyed. It is mandatory that the clinical staff involved in the clinical trial has completed an official course in Good Clinical Practice.

TRIAL STATUS

Recruitment opens January 2022 with first patient, first visit (FPFV), whereas last patient, last visit (LPLV) is scheduled for **September 2024.** Analysis of primary endpoints is planned to **March 2025**: Data will be published in EudraCT within 12 months from LPLV. Publication of the final results on primary endpoint is expected to be made public in **March 2025**. The results will be disseminated in peer review publications and conference presentations.

For peer review only - http://bmjopen.bmj.com/site/about/guidelines.xhtml

For peer review only

DISCUSSION

Metabolic disturbances, obesity and diabetes development in SGA treated patients are a major problem in the clinical setting, but the underlying mechanism is poorly understood, even though there are theories focusing on an interaction of medication, genes, life style, diet and physical activity [14] [9] [1] .

GLP-1 analogs have shown remarkable effect in the treatment of diabetes by contributing to weight loss, lowering blood glucose levels and improving mortality from CVD [51] [33,34]. This is in contrast to life style interventions with physical activity and diet changes, as such initiatives have failed to induce robust long-term improvements of the metabolic disturbances [53][25][24].

The main focus of this clinical trial is mitigation of metabolic aberrations due to SGA treatment and reversal of prediabetes. Hence, the primary outcome is the absolute reduction in HbA1c rather than weight loss.

Patients with severe mental illness experience many challenges due to schizophrenic symptoms and side effects to SGA treatment [12][13]. Recruitment may be difficult in this patient population, and accordingly, we are collaborating with local psychiatric teams in both Regions and with local consultants to create a secure environment. The study is designed to focus on adherence to study protocol and minimize the patient's burden of study participation. The home visit by study nurses is likely to strengthen protocol adherence by assisting the participants and creating a secure and safe environment, eventually yielding higher reliability of data from interviews and questionnaires.

Semaglutide holds benefits compared to Liraglutide when dealing with patients with weight gain caused by SGAs. Semaglutide requires weekly treatment only, which makes it easier to administer with help from a study-nurse. In addition, the weight reducing effect of Semaglutide is greater than Liraglutide without compromising the cardio-protective effect [54]. Hence, if Semaglutide 1.34 mg/ml has a beneficial effect on

For peer review only - http://bmjopen.bmj.com/site/about/guidelines.xhtml

weight, HbA1c, MetS and QoL, it could potentially be used in patients with schizophrenia in high risk of cardiovascular disease to prevent premature death due to diabetes.

Our study will attempt to mitigate the metabolic side effects of SGA induced overweight in individuals with schizophrenia and prediabetes. Treatment with Semaglutide improves glycemic control, weight and clinical cardiovascular risk factors by reducing numbers of fatal and non-fatal myocardial infarction and non-fatal stroke within 104 weeks [55]. We hypothesize that beneficial pre-clinical events are detectable by NAF uptake using PET-CT scans after 30 weeks of treatment. If so, this may contribute to a better understanding of the nature behind the increased cardiovascular risk within these patients.

We hope that our trial HISTORI will provide insight in how to benefit the cardio-metabolic health and the QoL of schizophrenic patients – adding more and better years - and thereby change the HISTORY of this vulnerable group of patients. Additionally, if successful, HISTORI may pave the way for long-term, large-scale GLP-1 agonist interventions in schizophrenic patients with prediabetes, aiming to reduce the number of hardcore clinical cardiovascular endpoints, and thereby prolonging their life expectancy. Finally, a positive outcome may fuel initiatives to study the ability of GLP-1 agonists to prevent the development of the prediabetes, e.g. by starting treatment shortly after treatment with SGAs are initiated in high-risk patients.

Funding Statement

Sources and types of financial, material, and other support funding for the study has been obtained from private foundations:

Novo Nordisk Foundation: 8,584,959DKK (salary and tuition fee PHD Students and study-nurses, running expenses, biochemical and laboratory procedures, TAP personal, mileage allowance)

For peer review only - http://bmjopen.bmj.com/site/about/guidelines.xhtml

Research Grant from Steno Diabetes Center Zealand, Denmark: 660,000 DKK.

Research Grant from Steno Diabetes Center Odense, Denmark: 578,000 DKK.

Annum (Region Zealand) to PhD-student Nicolai Uhrenholt: 20.000 DDK per year for three years (60,000 DKK in total).

Slagelse Puljen, 2020: The Region of Zealand, Denmark: 240,000 DKK

Slagelse Puljen 2022: 150,000 DKK

Region Sjællands Sundhedsvidenskabelige Forskningsfond 2022: 252,631 DKK. Furthermore, Novo Nordisk A/S will provide investigational drug and placebo free of charge for sponsor, but is otherwise not involved in the study. The scientists involved will conduct the experiment out of general scientific interest without personal financial gain. None of the investigators involved have financial interests (including shares, direct employment, members of advisory boards) in the drug company that produces the active drug used in the study. None of the participants in the study will receive any provision for participation. Participants will receive mileage allowances. Any harm as a result of study participation will be covered by the Danish Patient Compensation.

Roles and responsibilities

a. Contributorship statement:

AG, NU, SA, PG, NB, and JF initiated the study design, AG and NU helped with implementation and execution of the study. JF, NB, SA, and PGH are grant holders. JF and NB provided statistical expertise in clinical trial design, AG and NGU are conducting the primary statistical analysis.

For peer review only

For peer review only - http://bmjopen.bmj.com/site/about/guidelines.xhtml

414 AG made significant contributions to the study protocol in the design and the writing process in with
415 contributions from NGU. All authors contributed to refinement of the study protocol and approved the final
416 manuscript.

417 Data from this study belongs to the research group behind Project HISTORI and can be accessed after
418 application to the sponsor JF.

419 *Sponsor:*

420 Odense University Hospital

421 Steno Diabetes Centre Odense / Department of Endocrinology M. J.B. Winsløvvej 4. DK- 5000 Odense,
422 Denmark.

423 *Correspondence:*

424 Jan Frystyk, Clinical Professor, DMsc, Ph.D. Steno Diabetes Centre Odense / Department of Endocrinology
425 M. Odense University Hospital. J.B. Winsløvvej 4. DK- 5000 Odense, Denmark. Email: jan.frystyk@rsyd.dk

426

427 *Primary Investigator at the Region of Southern Denmark:*
428 Jan Frystyk, Clinical Professor, DMsc, Ph.D. Clinical management of diabetic patients at SDCO;
429 implementation of biomarkers in clinical studies, and PI on clinical studies, hereunder GCP-monitored RCT.
430 Department of Endocrinology M / Steno Diabetes Centre Odense (SDCO). Odense University Hospital. J.B.
431 Winsløvvej 4. DK- 5000 Odense, Denmark.

432 *Primary Investigator at the Region Zealand Community Psychiatry:*

433 Sidse Arnfred, Clinical Professor.
434 Specialist in family medicine, psychiatry and board certified supervisor in psychotherapy (CBT). Research
435 expertise in schizophrenia, psychopathology and psychophysiology. Experienced PI on RCT's, qualitative
436 studies of user experiences and treatment evaluation. Psykiatrien Region Sjælland**,** Psykiatrien Vest,
437 Fælledvej 6, bygning 3, 4. sal, DK- 4200 Slagelse, Denmark.

438

439 *Primary Investigator at the Region of Southern Denmark community Psychiatry*
440 Niels Bilenberg, Clinical Professor, Ph.D. OPUS clinics and community psychiatry centers of Region of
441 Southern. Mental Health Hospital and University Clinic in the Region of Southern Denmark. J.B.Winsløws
442 Vej 28 B, indg. 236, DK-5000 Odense, Denmark.

For peer review only - http://bmjopen.bmj.com/site/about/guidelines.xhtml

For peer review only

Co-investigators:

Peter Gæde, MD, DMSci, Chief Physician, Associate Professor. Department of Steno Diabetes Centre Sjælland. Steno Diabetes Center Sjælland, Akacievej 7, 1.sal. DK-4300 Holbæk, Denmark.

PhD students:

Ashok Ganeshalingam, Physician, Ph.D. Student. Department of Endocrinology M / Steno Diabetes Centre Odense (SDCO). Odense University Hospital. J.B. Winsløvvej 4. DK- 5000 Odense, Denmark.

Nicolai Gundtoft Uhrenholt, Physician, Ph.D. Student. Psykiatrien Region Sjælland**,** Psykiatrien Vest, Fælledvej 6, Bygning 3, 4. sal, DK- 4200 Slagelse, Denmark.

b. Competing interests:

The authors declare that there is no conflict of interest that would prejudice the impartiality of this scientific work.

c. Funding:

Novo Nordisk Foundation: 8,584,959DKK (salary and tuition fee PHD Students and study-nurses, running expenses, biochemical and laboratory procedures, TAP personal, mileage allowance)

Research Grant from Steno Diabetes Center Zealand, Denmark: 660,000 DKK.

Research Grant from Steno Diabetes Center Odense, Denmark: 578,000 DKK.

Annum (Region Zealand) to PhD-student Nicolai Uhrenholt: 20.000 DDK per year for three years (60,000 DKK in total).

For peer review only - http://bmjopen.bmj.com/site/about/guidelines.xhtml

Slagelse Puljen, 2020: The Region of Zealand, Denmark: 240,000 DKK

Slagelse Puljen 2022: 150,000 DKK

Region Sjællands Sundhedsvidenskabelige Forskningsfond 2022: 252,631 DKK.

Furthermore, Novo Nordisk A/S will provide investigational drug and placebo free of charge for sponsor, but is otherwise not involved in the study.

For peer review only

REFERENCES

1 D. S, M. H, C.U. C, *et al.* The prevalence of food addiction as assessed by the yale food addiction scale: A systematic review. *Eur J Nucl Med Mol Imaging*. 2017;74:719–28.

2 Lawrence D, Hancock KJ, Kisely S. The gap in life expectancy from preventable physical illness in psychiatric patients in Western Australia: Retrospective analysis of population based registers. *BMJ*. Published Online First: 2013. doi: 10.1136/bmj.f2539

3 Laursen TM, Plana-Ripoll O, Andersen PK, *et al.* Cause-specific life years lost among persons diagnosed with schizophrenia: Is it getting better or worse? *Schizophr Res*. 2019;206:284–90.

4 Correll CU, Solmi M, Croatto G, *et al.* World Psychiatry - 2022 - Correll - Mortality in people with schizophrenia a systematic review and meta-analysis of.pdf. 2022;249–71.

5 Nordentoft M, Wahlbeck K, Hällgren J, *et al.* Excess mortality, causes of death and life expectancy in 270,770 patients with recent onset of mental disorders in Denmark, Finland and Sweden. *PLoS One*. 2013;8:e55176.

6 Westman J, Eriksson S V., Gissler M, *et al.* Increased cardiovascular mortality in people with schizophrenia: A 24-year national register study. *Epidemiol Psychiatr Sci*. Published Online First: 2018. doi: 10.1017/S2045796017000166

7 De Hert M, Dekker JM, Wood D, *et al.* Cardiovascular disease and diabetes in people with severe mental illness position statement from the European Psychiatric Association (EPA), supported by the European Association for the Study of Diabetes (EASD) and the European Society of Cardiology (ESC. Eur. Psychiatry. 2009. https://doi.org/10.1016/j.eurpsy.2009.01.005

8 De Hert M, Correll CU, Bobes J, *et al.* Physical illness in patients with severe mental disorders. I. Prevalence, impact of medications and disparities in health care. *World Psychiatry*. 2011;10:52–77.

9 Vancampfort D, Stubbs B, Mitchell AJ, *et al.* Risk of metabolic syndrome and its components in

For peer review only - http://bmjopen.bmj.com/site/about/guidelines.xhtml

people with schizophrenia and related psychotic disorders, bipolar disorder and major depressive disorder: A systematic review and meta-analysis. *World Psychiatry*. 2015;14:339–47.

10 Leucht S, Cipriani A, Spineli L, *et al.* Comparative efficacy and tolerability of 15 antipsychotic drugs in schizophrenia: a multiple-treatments meta-analysis. *Lancet (London, England)*. 2013;382:951–62.

11 Tiihonen J, Wahlbeck K, Lönnqvist J, *et al.* Effectiveness of antipsychotic treatments in a nationwide cohort of patients in community care after first hospitalisation due to schizophrenia and schizoaffective disorder: Observational follow-up study. *Br Med J*. Published Online First: 2006. doi: 10.1136/bmj.38881.382755.2F

12 Maayan L, Correll CU. Weight gain and metabolic risks associated with antipsychotic medications in children and adolescents. *J Child Adolesc Psychopharmacol*. 2011;21:517–35.

13 Pillinger T, McCutcheon RA, Vano L, *et al.* Comparative effects of 18 antipsychotics on metabolic function in patients with schizophrenia, predictors of metabolic dysregulation, and association with psychopathology: a systematic review and network meta-analysis. *The lancet Psychiatry*. 2020;7:64–77.

14 Henderson DC, Vincenzi B, Andrea N V., *et al.* Pathophysiological mechanisms of increased cardiometabolic risk in people with schizophrenia and other severe mental illnesses. *The Lancet Psychiatry*. 2015;2:452–64.

15 DE Hert M, Correll CU, Bobes J, *et al.* Physical illness in patients with severe mental disorders. I. Prevalence, impact of medications and disparities in health care. *World Psychiatry*. 2011;10:52–77.

16 Allison DB, Mackell JA, McDonnell DD. The impact of weight gain on quality of life among persons with schizophrenia. *Psychiatr Serv*. 2003;54:565–7.

17 Strassnig M, Brar JS, Ganguli R. Body mass index and quality of life in community-dwelling patients with schizophrenia. *Schizophr Res*. 2003;62:73–6.

For peer review only

18 Faulkner G, Cohn T, Remington G, *et al.* Body mass index, waist circumference and quality of life in individuals with schizophrenia. *Schizophr Res*. 2007;90:174–8.

19 R.L. K, P.K. C-L, R.D. C, *et al.* Impact of obesity on health-related quality of life in schizophrenia and bipolar disorder. Obesity. 2008;16:749–54. http://ovidsp.ovid.com/ovidweb.cgi?T=JS&PAGE=reference&D=emed8&NEWS=N&AN=18239573

20 Sugawara N, Yasui-Furukori N, Sato Y, *et al.* Body mass index and quality of life among outpatients with schizophrenia in Japan. *BMC Psychiatry*. 2013;13. doi: 10.1186/1471-244X-13-108

21 Fond G, Korchia T, Sunhary de Verville PL, *et al.* Major depression, sleep, hostility and body mass index are associated with impaired quality of life in schizophrenia. Results from the FACE-SZ cohort. *J Affect Disord*. 2020;274:617–23.

22 Lieberman JA, Stroup TS, McEvoy JP, *et al.* Effectiveness of antipsychotic drugs in patients with chronic schizophrenia. *N Engl J Med*. 2005;353:1209–23.

23 Weiden PJ, Mackell JA, McDonnell DD. Obesity as a risk factor for antipsychotic noncompliance. *Schizophr Res*. 2004;66:51–7.

24 Cooper SJ, Reynolds GP, Barnes TRE, *et al.* BAP guidelines on the management of weight gain, metabolic disturbances and cardiovascular risk associated with psychosis and antipsychotic drug treatment. *J Psychopharmacol*. Published Online First: 2016. doi: 10.1177/0269881116645254

25 Holt RIG, Gossage-Worrall R, Hind D, *et al.* Structured lifestyle education for people with schizophrenia, schizoaffective disorder and first-episode psychosis (STEPWISE): Randomised controlled trial. *Br J Psychiatry*. 2019;214:63–73.

26 Zheng W, Li X-B, Tang Y-L, *et al.* Metformin for Weight Gain and Metabolic Abnormalities Associated With Antipsychotic Treatment: Meta-Analysis of Randomized Placebo-Controlled Trials. *J Clin Psychopharmacol*. 2015;35:499–509.

For peer review only - http://bmjopen.bmj.com/site/about/guidelines.xhtml

27 Larsen JR, Vedtofte L, Jakobsen MSL, *et al.* Effect of liraglutide treatment on prediabetes and overweight or obesity in clozapine- or olanzapine-treated patients with schizophrenia spectrum disorder: A randomized clinical trial. *JAMA Psychiatry*. Published Online First: 2017. doi: 10.1001/jamapsychiatry.2017.1220

28 Siskind D, Russell A, Gamble C, *et al.* RCT of exenatide for clozapine-associated obesity. *Schizophr Bull*. 2017.

29 Kouidrat Y, Amad A. GLP-1 agonists for metabolic disorders in schizophrenia. Schizophr. Res. 2019. https://doi.org/10.1016/j.schres.2018.09.019

30 D. S, M. H, C.U. C, *et al.* Glucagon-like peptide-1 receptor agonists for antipsychotic-associated cardio-metabolic risk factors: A systematic review and individual participant data meta-analysis. *Diabetes, Obes Metab*. 2019.

31 Rubino D, Abrahamsson N, Davies M, *et al.* Effect of Continued Weekly Subcutaneous Semaglutide vs Placebo on Weight Loss Maintenance in Adults with Overweight or Obesity: The STEP 4 Randomized Clinical Trial. *JAMA - J Am Med Assoc*. 2021;325:1414–25.

32 Fernández SG. Adherence to antipsychotic medication in bipolar disorder and schizophrenia patients: a systematic review. *Eur Neuropsychopharmacol*. Published Online First: 2016. doi: 10.1016/s0924-977x(16)31412-2

33 Muzurović E, Yumuk VD, Rizzo M. GLP-1 and dual GIP/GLP-1 receptor agonists in overweight/obese patients for atherosclerotic cardiovascular disease prevention: Where are we now? *J Diabetes Complications*. 2023;37:2023–5.

34 Lincoff AM, Brown-Frandsen K, Colhoun HM, *et al.* Semaglutide and Cardiovascular Outcomes in Obesity without Diabetes. *N Engl J Med*. 2023;389:2221–32.

35 El Abdellati K, De Picker L, Morrens M. Antipsychotic Treatment Failure: A Systematic Review on Risk Factors and Interventions for Treatment Adherence in Psychosis. *Front Neurosci*. 2020;14. doi:

For peer review only

For peer review only - http://bmjopen.bmj.com/site/about/guidelines.xhtml

10.3389/fnins.2020.531763

36 Kay SR, Fiszbein A, Opler LA. The positive and negative syndrome scale (PANSS) for schizophrenia. *Schizophr Bull*. Published Online First: 1987. doi: 10.1093/schbul/13.2.261

37 Østergaard SD, Foldager L, Mors O, *et al.* The Validity and Sensitivity of PANSS-6 in the Clinical Antipsychotic Trials of Intervention Effectiveness (CATIE) Study. *Schizophr Bull*. 2018;44:453–62.

38 Kølbæk P, Blicher AB, Buus CW, *et al.* Inter-rater reliability of ratings on the six-item Positive and Negative Syndrome Scale (PANSS-6) obtained using the Simplified Negative and Positive Symptoms Interview (SNAPSI). *Nord J Psychiatry*. 2018;72:431–6.

39 Ware JEJ, Sherbourne CD. The MOS 36-item short-form health survey (SF-36). I. Conceptual framework and item selection. *Med Care*. 1992;30:473–83.

40 Serhiyenko VA, Serhiyenko AA. Cardiac autonomic neuropathy: Risk factors, diagnosis and treatment. *World J Diabetes*. Published Online First: 2018. doi: 10.4239/wjd.v9.i1.1

41 Herder C, Roden M, Ziegler D. Novel Insights into Sensorimotor and Cardiovascular Autonomic Neuropathy from Recent-Onset Diabetes and Population-Based Cohorts. Trends Endocrinol. Metab. 2019. https://doi.org/10.1016/j.tem.2019.02.007

42 Poirier P, Hernandez TL, Weil KM, *et al.* Impact of diet-induced weight loss on the cardiac autonomic nervous system in severe obesity. *Obes Res*. Published Online First: 2003. doi: 10.1038/oby.2003.143

43 Moghbel M, Al-Zaghal A, Werner TJ, *et al.* The Role of PET in Evaluating Atherosclerosis: A Critical Review. Semin. Nucl. Med. 2018. https://doi.org/10.1053/j.semnuclmed.2018.07.001

44 McKenney-Drake ML, Moghbel MC, Paydary K, *et al.* 18F-NaF and 18F-FDG as molecular probes in the evaluation of atherosclerosis. Eur. J. Nucl. Med. Mol. Imaging. 2018. https://doi.org/10.1007/s00259-018-4078-0

For peer review only

For peer review only - http://bmjopen.bmj.com/site/about/guidelines.xhtml

45 Høilund-Carlsen PF, Moghbel MC, Gerke O, *et al.* Evolving Role of PET in Detecting and Characterizing Atherosclerosis. PET Clin. 2019. https://doi.org/10.1016/j.cpet.2018.12.001

46 Kolotkin RL, Crosby RD, Corey-Lisle PK, *et al.* Performance of a weight-related measure of Quality of Life in a psychiatric sample. *Qual life Res an Int J Qual life Asp Treat care Rehabil*. 2006;15:587–96.

47 Thompson K, Kulkarni J, Sergejew AA. Reliability and validity of a new Medication Adherence Rating Scale (MARS) for the psychoses. *Schizophr Res*. Published Online First: 2000. doi: 10.1016/S0920-9964(99)00130-9

48 Fialko L, Garety PA, Kuipers E, *et al.* A large-scale validation study of the Medication Adherence Rating Scale (MARS). *Schizophr Res*. 2008;100:53–9.

49 Rosenbaum S, Morell R, Abdel-Baki A, *et al.* Assessing physical activity in people with mental illness: 23-country reliability and validity of the simple physical activity questionnaire (SIMPAQ). *BMC Psychiatry*. 2020;20:108.

50 Rabin R, Gudex C, Selai C, *et al.* From translation to version management: a history and review of methods for the cultural adaptation of the EuroQol five-dimensional questionnaire. *Value Heal J Int Soc Pharmacoeconomics Outcomes Res*. 2014;17:70–6.

51 Sorli C, Harashima S ichi, Tsoukas GM, *et al.* Efficacy and safety of once-weekly semaglutide monotherapy versus placebo in patients with type 2 diabetes (SUSTAIN 1): a double-blind, randomised, placebo-controlled, parallel-group, multinational, multicentre phase 3a trial. *Lancet Diabetes Endocrinol*. Published Online First: 2017. doi: 10.1016/S2213-8587(17)30013-X

52 Larsen JR, Vedtofte L, Jakobsen MSL, *et al.* Effect of liraglutide treatment on prediabetes and overweight or obesity in clozapine- or olanzapine-treated patients with schizophrenia spectrum disorder: A randomized clinical trial. *JAMA Psychiatry*. 2017;74:719–28.

53 Speyer H, Christian Brix Nørgaard H, Birk M, *et al.* The CHANGE trial: No superiority of lifestyle coaching plus care coordination plus treatment as usual compared to treatment as usual alone in

For peer review only

For peer review only - http://bmjopen.bmj.com/site/about/guidelines.xhtml

616 reducing risk of cardiovascular disease in adults with schizophrenia spectrum disorders and
617 abdominal obesity. *World Psychiatry*. 2016;15:155–65.
618 54 Verma S, Al-Omran M, Leiter LA, *et al.* Cardiovascular efficacy of liraglutide and semaglutide in
619 individuals with diabetes and peripheral artery disease. *Diabetes, Obes Metab*. 2022;24:1288–99.
620 55 Marso SP, Bain SC, Consoli A, *et al.* Semaglutide and cardiovascular outcomes in patients with type 2
621 diabetes. *N Engl J Med*. Published Online First: 2016. doi: 10.1056/NEJMoa1607141

622

**Table 1**
**Inclusion criteria**
• Diagnosed with schizophrenia spectrum disorder (ICD10 codes DF20, DF21 or DF25)
• Age between 18 and 60 years (both included)
• Approved contraception for female participants
• Treated by one of the community mental health centers and general practitioners in the Region of Southern Denmark or Zealand
• Antipsychotic SGA treatment for at least 6 months
• Stable co-medication for at least 1 month
• HbA1c between 39-47 mmol/mol (both included). Two measurements with ≥3 month interval are required to confirm prediabetes. The first measurement is identified and obtained from patient journals, the second prior to enrolment

For peer review only - http://bmjopen.bmj.com/site/about/guidelines.xhtml

• BMI ≥27 kg/m². Two weights with ≥3 month interval are required to confirm obesity
• Capable of providing informed oral and written consent
**Exclusion criteria**
• Diagnosis of diabetes (T1D or T2D) or a HbA1c >47 mmol/mol
• Active malignant disease within the last 5 years
• Pregnancy or breast feeding
• A reported high consumption of alcohol or severe substance abuse
• Unwillingness to allow home visits by a study nurse
• Significant somatic disease: 1) end-stage renal failure (eGFR <15 ml/min); 2) elevated liver function tests (liver transaminases >2 times upper normal limit); 3) history of acute or chronic pancreatitis; 4) heart failure (NYHA class IV) or unstable angina pectoris or myocardial infarction with the last 6 months; 5) uncontrolled hypertension (systolic blood pressure >180 mm Hg, diastolic blood pressure >100 mm Hg)
• Previous treatment with study drug or use of other weight reducing drugs within last 6 month
• Participation in other drug trials
• Treatment with drugs approved for overt diabetes type 2 except metformin.
• Circumstances that the investigator believes will interfere with the trial

For peer review only - http://bmjopen.bmj.com/site/about/guidelines.xhtml

623

For peer review only

For peer review only - http://bmjopen.bmj.com/site/about/guidelines.xhtml

Figure Legend:

Figure 1 – Flow chart

For peer review only

For peer review only - http://bmjopen.bmj.com/site/about/guidelines.xhtml

For peer review only

For peer review only - http://bmjopen.bmj.com/site/about/guidelines.xhtml

CONSORT Flow Diagram

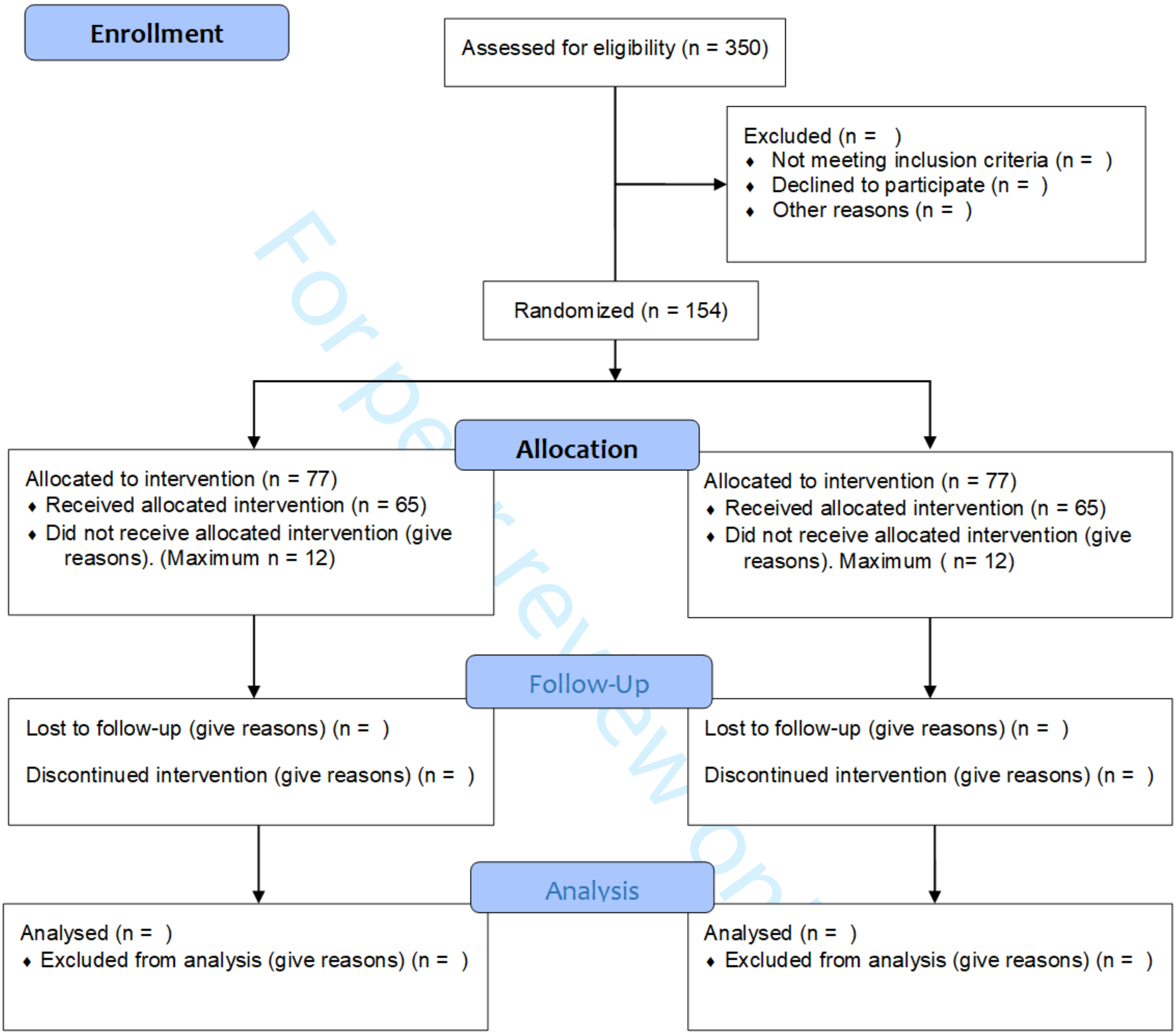

For peer review only - http://bmjopen.bmj.com/site/about/guidelines.xhtml

Supplementary Table 1

HISTORI	Investigator inclusion	Patient acceptance to participate	Week	Week	Week	Week	Week	Week	Week	Week	Week
			1	5	9	15	19	23	28	30	36
	Physical appearance of patients at hospitals	X				X				X	
			Weekly injection of Semaglutide / placebo								
Consent approval	X										
Baseline number		X									
Evaluation of medical history	X	X									
In- and Exclusion criteria	X	X									
Randomization		X									
Physical examination		X									
Blood pressure	X		X			X				X	
Weight/BMI	X		X			X				X	
Full biochemical screening		X								X	
Safety blood test	X					X					
PET CT scan		Scheduled							Scheduled		
PANNS-6, SF-36v2, IWQOL-Lite		X				X				X	
MARS, SIMPAQ, and EQ-5D		X	X	X	X	X	X	X	X	X	
Cardiac autonomic neuropathy (VAGUS®)		X				X				X	

**Adjustment of Semaglutide/placebo doses**				X	X						
**Semaglutide 0.25 mg**			X								
**Semaglutide 0.50 mg**				X							
**Semaglutide 1.00 mg per week**					X	X	X	X	X	X	
**End of study evaluation**											X

For peer review only

Reporting checklist for protocol of a clinical trial.

Based on the SPIRIT guidelines.

Instructions to authors

Complete this checklist by entering the page numbers from your manuscript where readers will find each of the items listed below.

Your article may not currently address all the items on the checklist. Please modify your text to include the missing information. If you are certain that an item does not apply, please write "n/a" and provide a short explanation.

Upload your completed checklist as an extra file when you submit to a journal.

In your methods section, say that you used the SPIRITreporting guidelines, and cite them as:

Chan A-W, Tetzlaff JM, Gøtzsche PC, Altman DG, Mann H, Berlin J, Dickersin K, Hróbjartsson A, Schulz KF, Parulekar WR, Krleža-Jerić K, Laupacis A, Moher D. SPIRIT 2013 Explanation and Elaboration: Guidance for protocols of clinical trials. BMJ. 2013;346:e7586

For peer review only

		Reporting Item	Page Number
**Administrative information**			
Title	#1	Descriptive title identifying the study design, population, interventions, and, if applicable, trial acronym	1

Trial registration	#2a	Trial identifier and registry name. If not yet registered, name of intended registry	3
Trial registration: data set	#2b	All items from the World Health Organization Trial Registration Data Set	3
Protocol version	#3	Date and version identifier	3
Funding	#4	Sources and types of financial, material, and other support	17-18
Roles and responsibilities: contributorship	#5a	Names, affiliations, and roles of protocol contributors	18
Roles and responsibilities: sponsor contact information	#5b	Name and contact information for the trial sponsor	18
Roles and responsibilities: sponsor and funder	#5c	Role of study sponsor and funders, if any, in study design; collection, management, analysis, and interpretation of data; writing of the report; and the decision to submit the report for publication, including whether they will have ultimate authority over any of these activities	18
Roles and responsibilities: committees	#5d	Composition, roles, and responsibilities of the coordinating centre, steering committee, endpoint adjudication committee, data management team, and	18-20

For peer review only

		other individuals or groups overseeing the trial, if applicable (see Item 21a for data monitoring committee)	
**Introduction**			
Background and rationale	#6a	Description of research question and justification for undertaking the trial, including summary of relevant studies (published and unpublished) examining benefits and harms for each intervention	4
Background and rationale: choice of comparators	#6b	Explanation for choice of comparators	4-6
Objectives	#7	Specific objectives or hypotheses	6
Trial design	#8	Description of trial design including type of trial (eg, parallel group, crossover, factorial, single group), allocation ratio, and framework (eg, superiority, equivalence, non-inferiority, exploratory)	7
**Methods: Participants, interventions, and outcomes**			
Study setting	#9	Description of study settings (eg, community clinic, academic hospital) and list of countries where data will be collected. Reference to where list of study sites can be obtained	7

For peer review only

Eligibility criteria	#10	Inclusion and exclusion criteria for participants. If applicable, eligibility criteria for study centres and individuals who will perform the interventions (eg, surgeons, psychotherapists)	7
Interventions: description	#11a	Interventions for each group with sufficient detail to allow replication, including how and when they will be administered	7-9
Interventions: modifications	#11b	Criteria for discontinuing or modifying allocated interventions for a given trial participant (eg, drug dose change in response to harms, participant request, or improving / worsening disease)	11-12
Interventions: adherance	#11c	Strategies to improve adherence to intervention protocols, and any procedures for monitoring adherence (eg, drug tablet return; laboratory tests)	8
Interventions: concomitant care	#11d	Relevant concomitant care and interventions that are permitted or prohibited during the trial	9
Outcomes	#12	Primary, secondary, and other outcomes, including the specific measurement variable (eg, systolic blood pressure), analysis metric (eg, change from baseline, final value, time to event), method of aggregation (eg, median, proportion), and time point for each outcome. Explanation of the clinical relevance of chosen efficacy and harm outcomes is strongly recommended	9-11

For peer review only

Participant timeline	#13	Time schedule of enrolment, interventions (including any run-ins and washouts), assessments, and visits for participants. A schematic diagram is highly recommended (see Figure)	Table 1.2
Sample size	#14	Estimated number of participants needed to achieve study objectives and how it was determined, including clinical and statistical assumptions supporting any sample size calculations	12
Recruitment	#15	Strategies for achieving adequate participant enrolment to reach target sample size	7
**Methods: Assignment of interventions (for controlled trials)**			
Allocation: sequence generation	#16a	Method of generating the allocation sequence (eg, computer-generated random numbers), and list of any factors for stratification. To reduce predictability of a random sequence, details of any planned restriction (eg, blocking) should be provided in a separate document that is unavailable to those who enrol participants or assign interventions	7
Allocation concealment mechanism	#16b	Mechanism of implementing the allocation sequence (eg, central telephone; sequentially numbered, opaque,	8

For peer review only

		sealed envelopes), describing any steps to conceal the sequence until interventions are assigned	
Allocation: implementation	#16c	Who will generate the allocation sequence, who will enrol participants, and who will assign participants to interventions	8
Blinding (masking)	#17a	Who will be blinded after assignment to interventions (eg, trial participants, care providers, outcome assessors, data analysts), and how	7
Blinding (masking): emergency unblinding	#17b	If blinded, circumstances under which unblinding is permissible, and procedure for revealing a participant's allocated intervention during the trial	12
**Methods: Data collection, management, and analysis**			
Data collection plan	#18a	Plans for assessment and collection of outcome, baseline, and other trial data, including any related processes to promote data quality (eg, duplicate measurements, training of assessors) and a description of study instruments (eg, questionnaires, laboratory tests) along with their reliability and validity, if known. Reference to where data collection forms can be found, if not in the protocol	9

For peer review only

Data collection plan: retention	#18b	Plans to promote participant retention and complete follow-up, including list of any outcome data to be collected for participants who discontinue or deviate from intervention protocols	table 1.2
Data management	#19	Plans for data entry, coding, security, and storage, including any related processes to promote data quality (eg, double data entry; range checks for data values). Reference to where details of data management procedures can be found, if not in the protocol	7
Statistics: outcomes	#20a	Statistical methods for analysing primary and secondary outcomes. Reference to where other details of the statistical analysis plan can be found, if not in the protocol	12
Statistics: additional analyses	#20b	Methods for any additional analyses (eg, subgroup and adjusted analyses)	12
Statistics: analysis population and missing data	#20c	Definition of analysis population relating to protocol non-adherence (eg, as randomised analysis), and any statistical methods to handle missing data (eg, multiple imputation)	12-14

Methods: Monitoring

Data monitoring: formal committee	#21a	Composition of data monitoring committee (DMC); summary of its role and reporting structure; statement of whether it is independent from the sponsor and competing interests; and reference to where further	14

For peer review only

		details about its charter can be found, if not in the protocol. Alternatively, an explanation of why a DMC is not needed	
Data monitoring: interim analysis	#21b	Description of any interim analyses and stopping guidelines, including who will have access to these interim results and make the final decision to terminate the trial	13-14
Harms	#22	Plans for collecting, assessing, reporting, and managing solicited and spontaneously reported adverse events and other unintended effects of trial interventions or trial conduct	11
Auditing	#23	Frequency and procedures for auditing trial conduct, if any, and whether the process will be independent from investigators and the sponsor	11
**Ethics and dissemination**			
Research ethics approval	#24	Plans for seeking research ethics committee / institutional review board (REC / IRB) approval	14
Protocol amendments	#25	Plans for communicating important protocol modifications (eg, changes to eligibility criteria, outcomes, analyses) to relevant parties (eg, investigators, REC / IRBs, trial participants, trial registries, journals, regulators)	14

For peer review only

Consent or assent	#26a	Who will obtain informed consent or assent from potential trial participants or authorised surrogates, and how (see Item 32)	7
Consent or assent: ancillary studies	#26b	Additional consent provisions for collection and use of participant data and biological specimens in ancillary studies, if applicable	7
Confidentiality	#27	How personal information about potential and enrolled participants will be collected, shared, and maintained in order to protect confidentiality before, during, and after the trial	15
Declaration of interests	#28	Financial and other competing interests for principal investigators for the overall trial and each study site	17
Data access	#29	Statement of who will have access to the final trial dataset, and disclosure of contractual agreements that limit such access for investigators	19
Ancillary and post trial care	#30	Provisions, if any, for ancillary and post-trial care, and for compensation to those who suffer harm from trial participation	19
Dissemination policy: trial results	#31a	Plans for investigators and sponsor to communicate trial results to participants, healthcare professionals, the public, and other relevant groups (eg, via publication, reporting in results databases, or other data sharing arrangements), including any publication restrictions	18

For peer review only

Dissemination policy: authorship	#31b	Authorship eligibility guidelines and any intended use of professional writers	18
Dissemination policy: reproducible research	#31c	Plans, if any, for granting public access to the full protocol, participant-level dataset, and statistical code	15

Appendices

Informed consent materials	#32	Model consent form and other related documentation given to participants and authorised surrogates	1
Biological specimens	#33	Plans for collection, laboratory evaluation, and storage of biological specimens for genetic or molecular analysis in the current trial and for future use in ancillary studies, if applicable	n/a

The SPIRIT Explanation and Elaboration paper is distributed under the terms of the Creative Commons Attribution License CC-BY-NC. This checklist was completed on 07. June 2023 using https://www.goodreports.org/, a tool made by the EQUATOR Network in collaboration with Penelope.ai

For peer review only
